# Vitamin K3 Analog Phthiocol Protects Against High Phosphate-Induced Vascular Calcification in Chronic Kidney Disease

**DOI:** 10.3390/antiox14111328

**Published:** 2025-11-04

**Authors:** Tsung-Jui Wu, Yi-Cheng Wang, Chia-Wen Lu, Chung-Jen Lee, Bang-Gee Hsu

**Affiliations:** 1 Institute of Medical Science, Tzu Chi University, Hualien 970374, Taiwan; int28@h805.mnd.gov.tw (T.-J.W.);; 2 Division of Nephrology, Department of Medicine, Hualien Armed Forces General Hospital, National Defense Medical Center, Hualien 971051, Taiwan; 3 Division of Nephrology, Hualien Tzu Chi Hospital, Buddhist Tzu Chi Medical Foundation, Hualien 970473, Taiwan; 4 Department of Nursing, Tzu Chi University, Hualien 970374, Taiwan

**Keywords:** chronic kidney disease, high-phosphorus diet, Nrf2/HO-1 antioxidation, PI3K/Akt pathway, phthiocol, vascular calcification

## Abstract

Vascular calcification (VC) is a multifactorial pathological deposition of calcium in the vasculature and is associated with severe cardiovascular outcomes, particularly in patients with chronic kidney disease (CKD). Various vitamin K analogs have been found to influence the development of VC. We utilized a high-phosphate-induced VC model in mouse vascular smooth muscle cells (VSMCs) and developed an in vivo VC model using ApoE^−/−^ mice subjected to 5/6 nephrectomy and fed an oral high-phosphorus diet to evaluate the effect of the vitamin K3 analog phthiocol. Transdermal glomerular filtration rate measurement, pulse wave velocity for aortic stiffness assessment, blood biochemical analysis, and pathological examinations were conducted. Phthiocol suppressed reactive oxygen species production and reduced subsequent cell death and calcification in a dose-dependent manner. It inhibited osteogenic trans-differentiation by restoring the PI3K/Akt pathway, activating Nrf2/HO-1 antioxidation signaling, and downregulating IL-1β and TNF-α. The high-phosphate diet in ApoE^−/−^ CKD mice significantly induced dyslipidemia, renal impairment, hyperphosphatemia, aortic stiffness, and calcium deposition in aortic tissue compared to the control group. Phthiocol treatment markedly improved dyslipidemia, hyperphosphatemia, and aortic stiffness. The vitamin K3 analog phthiocol ameliorates phosphate-induced osteogenic trans-differentiation of VSMCs and subsequent VC by restoring the PI3K/Akt pathway and enhancing Nrf2/HO-1 antioxidant activity.

## 1. Introduction

Vascular calcification (VC) is an old but common pathological process characterized by the deposition of calcium phosphate particles in the vasculature [1,2]. Calcification of the medial layer of blood vessels (Monckeberg’s medial sclerosis) is prevalent in aging, type 2 diabetes, and chronic kidney disease (CKD) [3,4]. Rather than a previously presumed degenerative deposition process, increasing evidence suggests an active, vascular smooth muscle cell (VSMC)-mediated mechanism occurring in response to an imbalance between pro-calcifying agonists (excess calcium, phosphate, advanced glycation end products, inflammation, oxidative stress) and inhibitors (matrix Gla protein, fetuin A) [5,6]. Transcription factors indicating osteo-chondrogenic trans-differentiation, including runt-related transcription factor 2 (Runx2) and msh homeobox 2 (MSX-2), have been identified in human arterial calcification in both the general population and in vitro [5,7]. In medial calcification, the stiffened vascular wall and reduced vessel compliance together lead to elevated pulse wave velocity (PWV), increased cardiac afterload, and higher pulse pressure, which consequently impair coronary artery perfusion and contribute to left ventricular hypertrophy [8]. Along with commonly coexisting atherosclerosis and suboptimal cardiovascular health indicators (metabolic syndrome, poor diet, physical inactivity, or smoking), VC progresses from clinically silent to a significant cardiovascular disease risk [9]. Current treatments focus on reducing calcium and phosphate burden in the CKD population. Alkaline loading, pyrophosphate, and bisphosphonates have been found to mitigate calcification, but these effects remain limited to animal studies [10,11]. The effectiveness of antioxidant treatment in reducing arterial stiffness has yet to be demonstrated in a comprehensive translational study [12].

Vitamin K (VK) comprises a group of fat-soluble molecules sharing a common 2-methyl-1,4-naphthoquinone nucleus (menadione) and a variable polyisoprenoid side chain in both length and saturation at the 3-position. VK1, found mainly in green leafy vegetables, is involved in synthesizing coagulation factors in the liver by activating VK-dependent proteins, while VK2 consists of bacterial forms also known as menaquinones (MKs) [13]. Beyond coagulation, the role of VK in cardiovascular disease progression and mortality is increasingly recognized [14], particularly through its influence on extrahepatic VK-dependent proteins (matrix Gla protein [MGP], osteocalcin) and their impact on VC [15,16]. Moreover, VK and the VK2 analog menaquinone-4 have been shown to exert anti-inflammatory effects by inhibiting the nuclear factor-κB (NF-κB) and interleukin-6 (IL-6) pathways induced by endotoxin in cell models [17,18,19]. However, a systematic review of clinical trials concluded that neither VK1 nor VK2 sufficiently mitigates VC in CKD patients [20]. To enhance the antioxidant activity of VK analogs, the VK3 analog phthiocol (2-hydroxy-3-methylnaphthalene-1,4-dione) was synthesized following Borovkov’s method [21]. These candidate compounds mostly demonstrated lower IC50 values in tumor cells, while IC50 values in normal human cell lines were generally >10 µM. In this study, we investigated the effects of phthiocol on high phosphate-induced VC using both a cell model and a CKD mouse model.

## 2. Materials and Methods

### 2.1. Materials and Reagent

Antibodies against Runx2 protein were purchased from Abcam (Abcam, Cambridge, UK); phosphoinositide 3-kinases (PI3Ks) and phosphorylated (p)-PI3K were obtained from Cell Signaling Technology (CST, Danvers, MA, USA); serine–threonine kinase (Akt) and p-Akt from Invitrogen (Carlsbad, CA, USA); and nuclear factor erythroid-2-related factor 2 (Nrf2), p-Nrf2, heme oxygenase 1 (HO-1), tumor necrosis factor alpha (TNF-α), interleukin (IL)-1β, and IL-6 proteins from ABclonal (ABclonal, Woburn, MA, USA). Inhibition of PI3K/Akt-dependent signaling pathways was performed by treating MOVAS cells with PI3K inhibitors LY294002 and wortmannin, the Nrf2 inhibitor ML385, and the HO-1 inhibitor zinc protoporphyrin-9 (ZnPP9, SC-200329), all purchased from Sigma-Aldrich (St. Louis, MO, USA). The Bio-Rad protein assay reagent was obtained from Bio-Rad (Bio-Rad Laboratories, Hertfordshire, UK); Alizarin Red S stain from PanReac AppliChem (PanReac AppliChem, Monza, Italy); Dulbecco Modified Eagle’s Medium (DMEM) and Gibco fetal bovine serum (FBS) from Life Technologies (Life Technologies Inc., Gaithersburg, MD, USA); and antibiotic G418, sodium phosphate monobasic, radioimmunoprecipitation assay (RIPA) lysis buffer, cOmplete^TM^ phosphatase inhibitor cocktail, and other reagents from Sigma-Aldrich (Sigma-Aldrich, St. Louis, MO, USA). All chemicals were prepared and stored according to the manufacturers’ recommendations.

### 2.2. Vascular Calcification Cell Model

We utilized a mouse VSMC line, MOVAS, obtained from the American Type Culture Collection (ATCC; Manassas, VA, USA; CRL-2797). The cells were cultured in high-glucose DMEM supplemented with 10% FBS and 0.2 mg/mL G418. G418, an aminoglycoside antibiotic, was used to maintain selection pressure. We followed the ATCC-recommended concentration (typically 0.2 mg/mL) to minimize cytotoxicity and avoid interference with overall protein expression. For experiments, cells were incubated at 37 °C in a humidified atmosphere with 5% CO_2_ for 24 h and subcultured every second day or when they reached 70–80% confluence. After thorough washing with phosphate-buffered saline, the VSMCs were cultured in normal or high-Pi medium (2.6 mM sodium phosphate monobasic) with Phthiocol at 0, 1.25, 2.5, 5, or 10 μM. [22]. During this period, the cells were subcultured every 2 days. For time-course experiments, the first day of culture in the calcification medium was defined as day 1.

### 2.3. Alizarin Red S Stain

To determine calcification of VSMCs, Alizarin Red S staining was performed after a 10-day incubation. After fixation with 95% ethanol for 30 min at room temperature, the cells were stained with Alizarin Red S (0.1%, pH 4.3) at 37 °C in a humidified atmosphere with 5% CO_2_. Alizarin Red S staining was quantified using a spectrophotometric method as suggested. After staining, the bound dye was eluted using 10% formic acid, and the absorbance of the eluted solution was measured at 420 nm using a microplate reader to assess the extent of calcium deposition. Calcified areas were stained red and photographed using a light microscope, then quantified with ImageJ software version 1.52a. The data were presented as fold changes relative to the control group.

### 2.4. Western Blotting

For each of three independent experiments, total protein from VSMCs was extracted after treatment with RIPA buffer containing a protease inhibitor and phosphatase inhibitor cocktail. After centrifugation at 12,000× *g* for 30 min at 4 °C, the concentration of extracted protein was measured using the Bio-Rad protein assay reagent, with BSA as the standard. Following protein denaturation, equal amounts of protein were separated by sodium dodecyl sulfate–polyacrylamide gel electrophoresis (SDS-PAGE) and transferred to polyvinylidene fluoride (PVDF) membranes. The intact membrane was imaged (with molecular-weight markers visible) and, while applicable, stained with Ponceau S. The membranes were then cut into strips (by molecular weight range) then blocked with 5% nonfat milk. Following the same treatment, each strip was incubated overnight at 4 °C with primary antibodies (Runx2, HO-1, Nrf2, p-Nrf2, IL-1β, TNF-α, PI3K, p-PI3K, AKT, p-AKT), followed by incubation with appropriate horseradish peroxidase-conjugated secondary antibodies and detection using enhanced chemiluminescence (ECL, GE Healthcare, Chicago, IL, USA). Western blot chemiluminescent signals were captured using a BioSpectrum Imaging 810 system (UVP LLC, Upland, CA, USA) and quantified with ImageJ software version 1.52a, normalized to the control.

### 2.5. Reactive Oxidative Species (ROS), Ferroptosis, and Live/Dead Cell Analyses

After various treatments, freshly collected VSMCs were subjected to flow cytometry analysis for reactive oxygen species (ROS) using the cell-permeant 2’,7’-dichlorodihydrofluorescein diacetate (DCFDA, Abcam, Cambridge, UK), and for cell viability using the Zombie NIR™ Fixable Viability Kit (BioLegend, San Diego, CA, USA). Cells were incubated with the respective reagents for 30 min in PBS before analysis.

### 2.6. Vascular Calcification Animal Model

Eight male C57BL/6 mice (6 weeks old, weighing 20–25 gm) from BioLASCO (Taipei, Taiwan) and 16 male Apoe^tm1Unc^/J mice (6 weeks old, weighing 20–25 gm) from Jackson Laboratory (Bar Harbor, ME, USA) were divided into three groups with 8 mice each: C57BL/6 sham surgery mice fed a normal chow diet (control group), ApoE^−/−^ mice with 5/6 nephrectomy (5/6 Nx) fed a high-phosphorus diet (VC group), and ApoE^−/−^ mice with 5/6 nephrectomy fed a high-phosphorus diet plus 5 mg/Kg phthiocol (VCP group). All mice were housed at room temperature (22–25 °C) under a 12:12 h light–dark cycle with ad libitum access to food and water at the Laboratory Animal Center of Tzu Chi University (Hualien, Taiwan). All experimental protocols were approved in advance by the Institutional Animal Care and Use Committee of Hualien Tzu Chi Hospital (approval number 109-10), and all experimental procedures were conducted in accordance with the Animal Research: Reporting of In Vivo Experiments (ARRIVE) guidelines. The 5/6 Nx procedure was performed in two stages [22,23,24]. Mice were anesthetized with inhaled isoflurane (Forane, Baxter, Deerfield, IL, USA) using a vaporizer delivery system (Matrx VIP 3000, Midmark, Dayton, OH, USA). In the first stage, the right kidney was removed. Seven days later, ligation of the upper branch of the left renal artery was performed using an absorbable 6-0 catgut suture. Following 5/6 Nx, VC and VCP mice were fed a high-phosphorus diet (1.5% total phosphorus, product #D09051102, Research Diets, New Brunswick, NJ, USA), without (VC) or with (VCP) phthiocol 5 mg/kg for 8 weeks.

### 2.7. Pulse Wave Velocity and Transdermal Glomerular Filtration Rate Measurement

PWV was assessed by measuring the cardiovascular pulse travel distance (d, in meters) and the transit time (Δt, in seconds). The procedures were performed according to the manufacturer’s protocols using lead II recording (PowerLab/8sp, BIO Amp, ADInstruments, Dunedin, New Zealand), and the pulse wave of the left ankle was detected with a pulse oximeter (MouseOx, Starr Life Sciences, Oakmont, PA, USA). The distance between the supraclavicular notch and the left hindlimb ankle of each mouse was measured in the supine position. Transit time (Δt) was determined as the time difference between the ECG R-wave and the peak of the left ankle pulse wave (LabChart software v7, ADInstruments, New Zealand). PWV was then calculated as follows: PWV = d/Δt [22].

The glomerular filtration rate (GFR) of mice was determined by transcutaneous measurement of fluorescein isothiocyanate (FITC)-labeled sinistrin (FITC-sinistrin) elimination kinetics. Following isoflurane anesthesia as described earlier, the hair on a portion of the back was removed, and the transdermal GFR (tGFR) Monitor (MediBeacon, St. Louis, MO, USA) was attached to this area. FITC-sinistrin (Mannheim Pharma and Diagnostics, Mannheim, Germany) at a dose of 0.15 mg per g body weight was administered into the retro-orbital venous sinus. Measurements were recorded for 1.5 h, and tGFR was calculated from the half-life of FITC-sinistrin using the software (MPDStudio Version RC15, MediBeacon, St. Louis, MO, USA) [22,25].

### 2.8. Tissue Collection, Biochemical Analysis, and Von Kossa Staining

In our experiment, carbon dioxide was used for euthanasia. The small cage had a volume of 8.5 L, and carbon dioxide was delivered at a rate of 3.4 L/min, equivalent to 40% of the chamber volume per minute, to reduce respiratory distress and accelerate loss of consciousness, in accordance with the 2020 AVMA Guidelines for the Euthanasia of Animals. Blood samples were collected after sacrifice and processed by centrifugation at 10,000× *g* for 10 min at 4 °C. Serum biochemical measurements of total cholesterol, triglycerides, high-density lipoprotein cholesterol (HDL-C), glucose, blood urea nitrogen (BUN), creatinine, calcium, and phosphorus were performed using a biochemistry analyzer (Spotchem SP-4430, Arkray, Minneapolis, MN, USA). Sections of the descending aorta were fixed in 4% buffered formaldehyde and subjected to von Kossa staining. After deparaffinization, rehydration, and incubation in 5% silver nitrate solution for 60 min under a 100-watt incandescent lamp, the tissue specimens were treated with 5% sodium thiosulfate solution for 2 min and then incubated in nuclear fast red solution for 5 min, with distilled water rinses before and after incubation. The slides were subsequently dehydrated, mounted, and examined under light microscopy. Calcium deposition was evaluated based on the integrated optical density of positive reactions using Image-Pro Plus 6.0 software.

### 2.9. Statistical Analysis

Data were presented as the mean ± standard error of the mean (SEM). For comparisons for experiments involving three or more groups, one-way analysis of variance (ANOVA) was performed to assess overall statistical significance, followed by Tukey Honest Significant Difference (HSD) post hoc test to control to identify multiple pairwise differences. A *p* < 0.05 was considered statistically significant. Statistical analysis was conducted using the Statistical Package for the Social Sciences, version 19.0 (IBM Corp., Armonk, NY, USA).

## 3. Results

The dose range of phthiocol (1.25–10 μM) used in this study was selected based on prior reports of structurally related vitamin K derivatives. Cui et al. demonstrated that menaquinone-4 (MK-4), a vitamin K2 analog, showed no significant cytotoxicity in VSMCs at concentrations between 10 and 50 μM [26]. Therefore, we applied a range of 1.25–10 μM for phthiocol in the present study. The absence of cytotoxicity was further confirmed using WST-1 assays before proceeding with downstream mechanistic experiments (Supplementary Figure S1).

**Figure 1:**
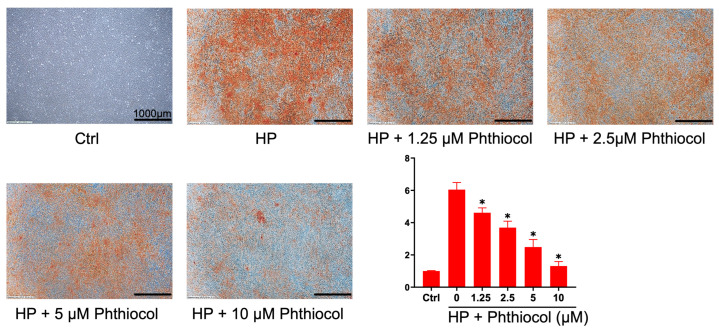
Alizarin Red S staining of mouse vascular smooth muscle cells (VSMCs) cultured in normal medium (control), high Pi (HP) medium, and HP with different concentrations (0, 1.25, 2.5, 5, 10 μM) of phthiocol. The staining intensity correlates with the degree of calcification. Results are expressed as mean ± SEM, and HP treatment groups (1.25–10 μM phthiocol) are compared with the HP (0 μM phthiocol) group using one-way ANOVA followed by Tukey HSD post hoc test (* *p* < 0.05); the control group is not included in the comparisons.

**Figure 2:**
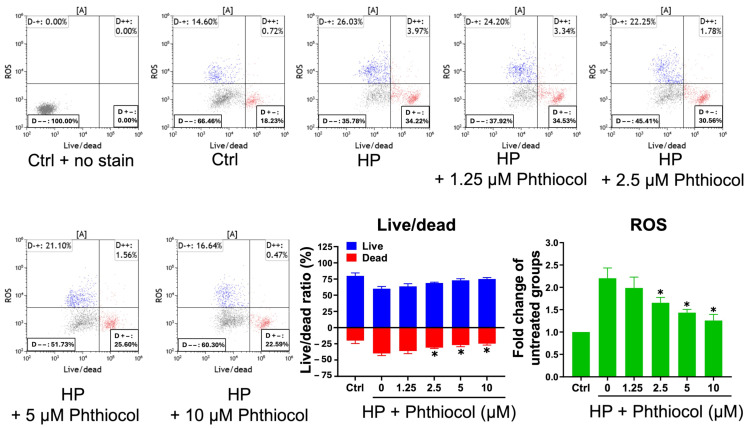
Reactive oxygen species production determined by flow cytometry of VSMCs cultured in blank, high Pi (HP) medium, and different concentrations (0, 1.25, 2.5, 5, 10 μM) of phthiocol. Quantification of live and dead cells, expressed as a percentage of total cells. Quantification fold change from blank of ROS levels was presented as mean fluorescence intensity ± SEM from three independent experiments. Results are expressed as mean ± SEM, and HP treatment groups (1.25–10 μM phthiocol) are compared with the HP (0 μM phthiocol) group using one-way ANOVA followed by Tukey HSD post hoc test (* *p* < 0.05); the control group is not included in the comparisons.

We first performed Alizarin Red S staining to examine whether the VK3 analog phthiocol could protect VSMCs from inorganic phosphate (Pi)-induced calcification. Calcium deposition was induced by high-Pi medium and was reduced by co-treatment with phthiocol in a dose-dependent manner (Figure 1). Flow cytometry further showed that high-Pi-induced cell damage and ROS production (Figure 2) were attenuated by phthiocol, also in a dose-dependent fashion.

**Figure 3:**
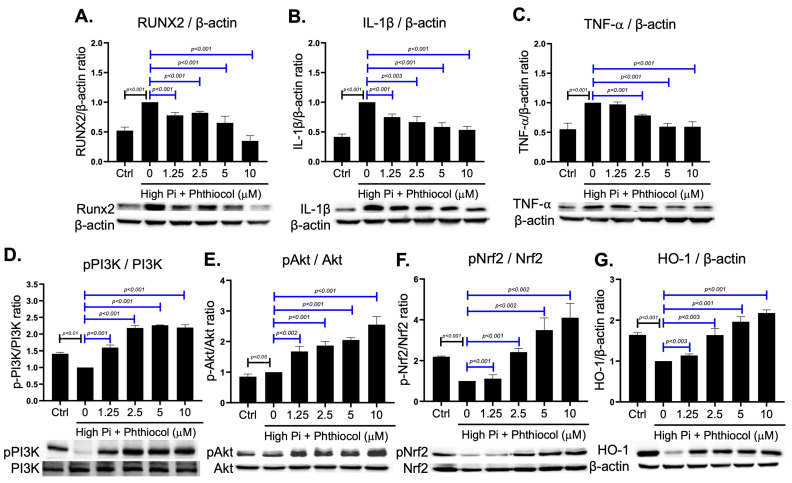
Representative Western blots and the relative protein expression levels of Runx2 (**A**), IL-1β (**B**), TNF-α (**C**), HO-1 (**G**) versus home keeping protein β-actin, and phosphorylated form versus total form of PI3K (**D**), Akt (**E**), and Nrf2 (**F**) in VSMC cultured in blank, high Pi (HP) medium, and HP with different concentrations of phthiocol. Data are presented as mean ± SEM from three biologically independent experiments and used for subsequent statistical analysis. Results are expressed as mean ± SEM, and HP treatment groups (1.25–10 μM phthiocol) are compared with the HP (0 μM phthiocol) group using one-way ANOVA followed by Tukey HSD post hoc test.

In Western blotting, we demonstrated that Pi acted as a strong inducer of inflammatory cytokine production and Runx2-mediated osteo-chondrogenic trans-differentiation. Phthiocol reduced the protein levels of Runx2, IL-1β, and TNF-α (Figure 3A–C) by restoring the expression of phosphorylated (p)-PI3K, p-Akt, p-Nrf2, and HO-1, suggesting activation of an anti-oxidative mechanism (Figure 3D–G).

**Figure 4:**
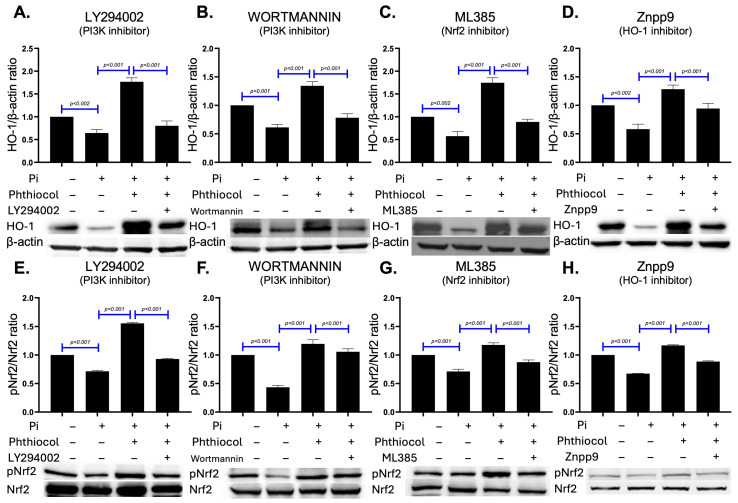
Representative Western blots and the relative protein expression level of HO-1 (**A**–**D**) versus β-actin and phosphorylated form versus total form of Nrf2 (**E**–**H**) in VSMC cultured in blank, high Pi (HP) medium, phthiocol and PI3K (LY294022, Wormannin), Nrf2 (ML385), or HO-1 (Znpp9) inhibitors. Results are expressed as mean ± SEM from three biologically independent experiments, and the Ctrl group or HP treatment groups (1.25–10 μM phthiocol) were compared with the HP (0 μM phthiocol) group using one-way ANOVA followed by Tukey HSD post hoc test.

We next tested whether the antioxidant protection of phthiocol was mediated through the PI3K/Akt pathway using chemical inhibition. Upon PI3K inhibition, the phthiocol-induced restoration of both HO-1 (Figure 4A–D) and phosphorylated Nrf2 (Figure 4E–H) against Pi-induced cell damage was markedly diminished. These findings support a critical role of the PI3K/Akt pathway in mediating the protective effects of phthiocol against phosphate-induced oxidative stress and cell death in VSMCs.

**Figure 5:**
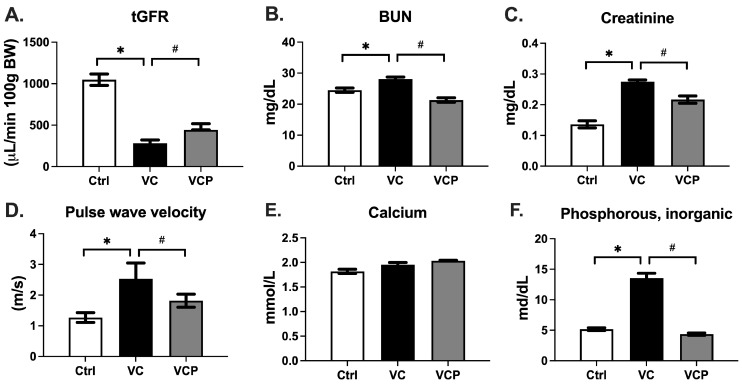

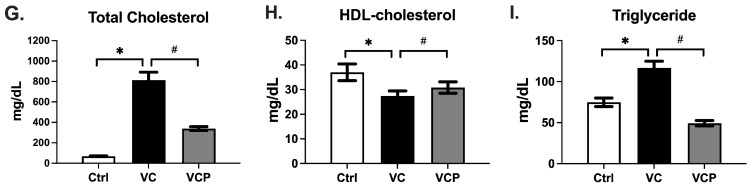
Transcutaneous glomerular filtration rate (tGFR) measurements (**A**), pulse wave velocity (PWV, (**D**)), and blood biochemistry parameters (**B**,**C**,**E**–**I**) in C57BL6 mice (Ctrl, *n* = 8) and CKD vascular calcification (VC) mice without phthiocol (VC, *n* = 8) or with phthiocol (VCP, *n* = 8). Data were expressed as mean ± SEM. *p* < 0.05 defines significant difference between the VC group and Ctrl group (*) or VC group and VCP group (#) using one-way ANOVA followed by Tukey HSD post hoc test.

We then examined whether the protective effects of phthiocol could be translated into the CKD mouse VC model. Compared with the control group, reduced tGFR and elevated PWV in the VC group indicated that CKD mice fed a high-Pi diet developed significant aortic stiffness. Aortic stiffness, measured by PWV, was ameliorated by phthiocol treatment in the VCP group (Figure 5A,D). In related biochemical analyses, renal impairment (BUN, creatinine) (Figure 5B,C), hyperphosphatemia (Figure 5F), and dyslipidemia (elevated total cholesterol, elevated triglycerides, and reduced HDL-C) (Figure 5G–I) induced by CKD with a high-Pi diet were also improved with phthiocol treatment. Serum calcium levels did not differ significantly between groups (Figure 5E). This supports our model that manipulating serum phosphate, rather than calcium, activates type III sodium-dependent phosphate cotransporter (PiT-1) in VSMCs, increases phosphate uptake, upregulates Runx2, and promotes vascular calcification [27].

**Figure 6:**
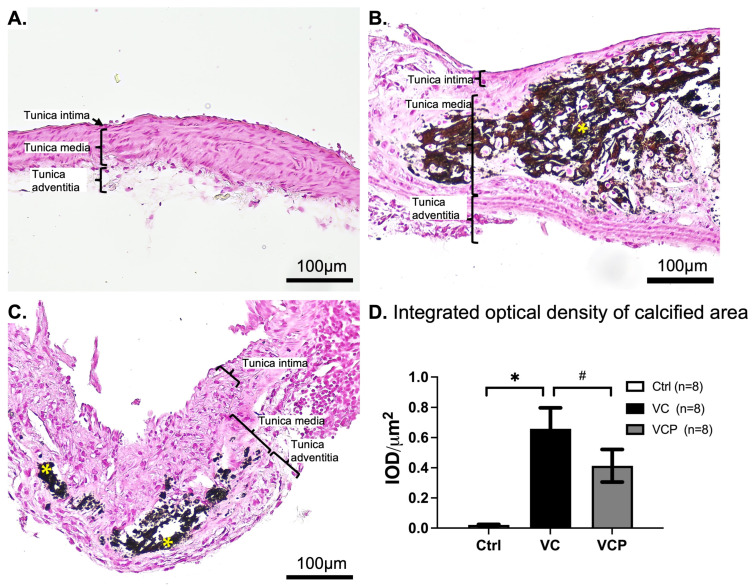
Representative images of transverse sections of the mouse aorta by Von Kossa staining from the control group (Ctrl, (**A**), *n* = 8), CKD vascular calcification mice without phthiocol (VC, (**B**), *n* = 8), and with phthiocol (VCP, (**C**), *n* = 8). Each panel (**A**–**C**) displays the three distinct layers of the aortic wall: tunica intima, tunica media, and tunica adventitia. Asterisk (*) indicates calcium deposition in the tunica media. Quantification results (**D**) of the von Kossa stain were assessed based on the averaged optical density [integrated optical density per square micrometers, (IOD/µm^2^)] of positive reactions using Image-Pro Plus 6.0 software. Data were expressed as mean ± SEM. *p* < 0.05 defines significant difference between the VC group and Ctrl group (✱) or VC group and VCP group (#) using one-way ANOVA followed by Tukey HSD post hoc test.

Transverse aortic sections from all experimental groups were subjected to von Kossa staining to assess calcium deposition. The VC and VCP groups showed thickened intima layer, but no foam cells or atheroma were observed in the VCP group. Positive staining (black or brown-black) was abundant in sections from the VC group but markedly reduced in those from the VCP group. Quantification with Image-Pro software showed that calcification scores were significantly increased by CKD with a high-Pi diet and decreased by phthiocol treatment (Figure 6).

## 4. Discussion

This study investigated the protective effects of the VK3 analog phthiocol against high-phosphate-induced VC in cultured cells and CKD mice. By restoring the PI3K/Akt pathway, phthiocol enhanced the Nrf2-mediated antioxidant response, thereby reducing cell damage and subsequent calcium deposition. In CKD mice, phthiocol improved aortic stiffness, dyslipidemia, hyperphosphatemia, and renal impairment, while also reducing aortic calcification. To the best of our knowledge, this is the first study to demonstrate the effectiveness of a vitamin K3 analog against VC.

Similar to VK2, which is intestinally converted from dietary phylloquinone, phthiocol has been detected in human lung cells infected with Mycobacteria [28]. With evidence of its natural occurrence in vivo, phthiocol was also shown to bind the aryl hydrocarbon receptor (AhR), a ligand-dependent transcription factor involved in detoxification and central to the defense against acute and chronic infection [29]. Upon binding AhR, phthiocol activated innate pulmonary defense mechanisms by upregulating downstream genes such as CYP1B1 and AHRR. In addition, phthiocol competitively bound the Pseudomonas quinolone signal (PQS) receptor, reducing the production of the virulence factor pyocyanin and inhibiting biofilm formation during Pseudomonas aeruginosa infection [30]. In an autoimmune uveitis murine model, AhR was shown to regulate immune responses through STAT/NF-κB signaling and the subsequent production of pro-inflammatory cytokines, including TNF-α, IL-6, and IL-1β. These studies support our findings that phthiocol protects against high-phosphate-induced VC.

The mechanistic role of PI3k/Akt in Nrf2 nuclear translocation has been demonstrated in response to oxidative stress through the antioxidant response element (ARE), intracellular calcium regulation, and transactivation of CCAAT/enhancer binding protein-beta (C/EBPβ) and peroxisome proliferator-activated receptor-gamma/retinoid X receptor (PPAR*γ*–RXR) [31]. Consistent with our findings, PI3K/Akt-mediated Nrf2 activation has been reported in various cell types exposed to stressors such as hyperoxia or inflammation [32,33,34].

There are, however, several limitations and opportunities for improvement. First, instead of directly conducting a comprehensive flow cytometry to examine the dose range, we relied on previously published results in which phthiocol was synthesized from vitamin K [21,35] and a simple cell viability assay using WST-1. Second, although AhR is thought to be highly conserved, molecular docking of phthiocol onto AhR and validation of its role in our CKD mouse VC model were not performed. Third, because vitamin K is lipophilic and shares transporters with cholesterol, it has been suggested that vitamin K may influence cholesterol regulation through effects on transport or metabolism. In advanced CKD, altered transport and tissue uptake of vitamin K2 may diminish the cardiovascular benefits of dietary supplementation [36]. While we observed a significant reduction in total cholesterol and a modest increase in HDL-C, serum and tissue vitamin K levels were not measured after vitamin K3 administration, limiting our ability to link vitamin K status to VC severity. Fourth, a comprehensive transcriptomic or proteomic analysis of the molecular interplay would provide a stronger basis for future experiments to clarify the protective role of phthiocol. With deeper mechanistic insights, this vitamin K3 analog may warrant further translational research before application in human VC disease.

## 5. Conclusions

Our research demonstrated that the vitamin K3 analog phthiocol reduced VSMC damage by restoring the PI3K/Akt pathway and activating the Nrf2/HO-1 antioxidant response to high-phosphate-induced oxidative stress and osteo-chondrogenic trans-differentiation. In the CKD animal model, phthiocol also alleviated high-phosphate-diet-induced aortic stiffness and calcification while improving renal impairment, dyslipidemia, and hyperphosphatemia.

## Data Availability

Data is contained within the article or supplementary material.

## Supplementary Materials

The following supporting information can be downloaded at: https://www.mdpi.com/article/10.3390/antiox14111328/s1, Figure S1: Phthiocol is non-cytotoxic at 1.25–10 μM by WST-1 assay; the comparisons between figure and original Western blots.

## Abbreviations

The following abbreviations are used in this manuscript:

AhR	Aryl hydrocarbon receptor
Akt	Serine/Threonine Kinase
ApoE	Apolipoprotein E
ARE	Antioxidant response element
BUN	Blood urea nitrogen
CKD	Chronic kidney disease
HDL-C	High-density lipoprotein cholesterol
HO-1	Heme Oxygenase-1
IL-1β	Interleukin-1 beta
MK-4	Menaquinone-4
MGP	matrix Gla protein
MSX-2	Msh homeobox 2
NF-κB	Nuclear factor-κB
Nrf2	Nuclear factor erythroid 2-related factor 2
Nx	Nephrectomy
PWV	Pulse wave velocity
PI3K	Phosphoinositide 3-kinase
Pi	Inorganic phosphate
PQS	Pseudomonas quinolone signal
ROS	Reactive oxygen species
Runx2	Runt-related transcription factor 2
tGFR	Transdermal glomerular filtration rate measurement
TNF-*α*	Tumor Necrosis Factor alpha
VC	Vascular calcification
VK	Vitamin K
VSMC	Vascular smooth muscle cell
