# Supplementary material for: Vitamin K3 Analog Phthiocol Protects Against High Phosphate-Induced Vascular Calcification in Chronic Kidney Disease
Source: Antioxidants (Basel). 2025 Nov 4;14(11):1328. doi: 10.3390/antiox14111328 (PMC12649243; doi:10.3390/antiox14111328)

Supplementary Files
for

Title:
Vitamin K3 analog phthiocol protects against high phosphate-induced vascular calcification in chronic kidney disease

Authors:
Tsung-Jui Wu [1,2,†], Yi-Cheng Wang [3,†], Chia-Wen Lu [1,2], Chung-Jen Lee [4], and Bang-Gee Hsu [1,3,*]

Includes:
1. Supplementary Figure S1
2. Raw Western blots and comparisons between the figure

Supplementary Figure S1. Phthiocol is non-cytotoxic at 1.25–10 μM by WST-1 assay

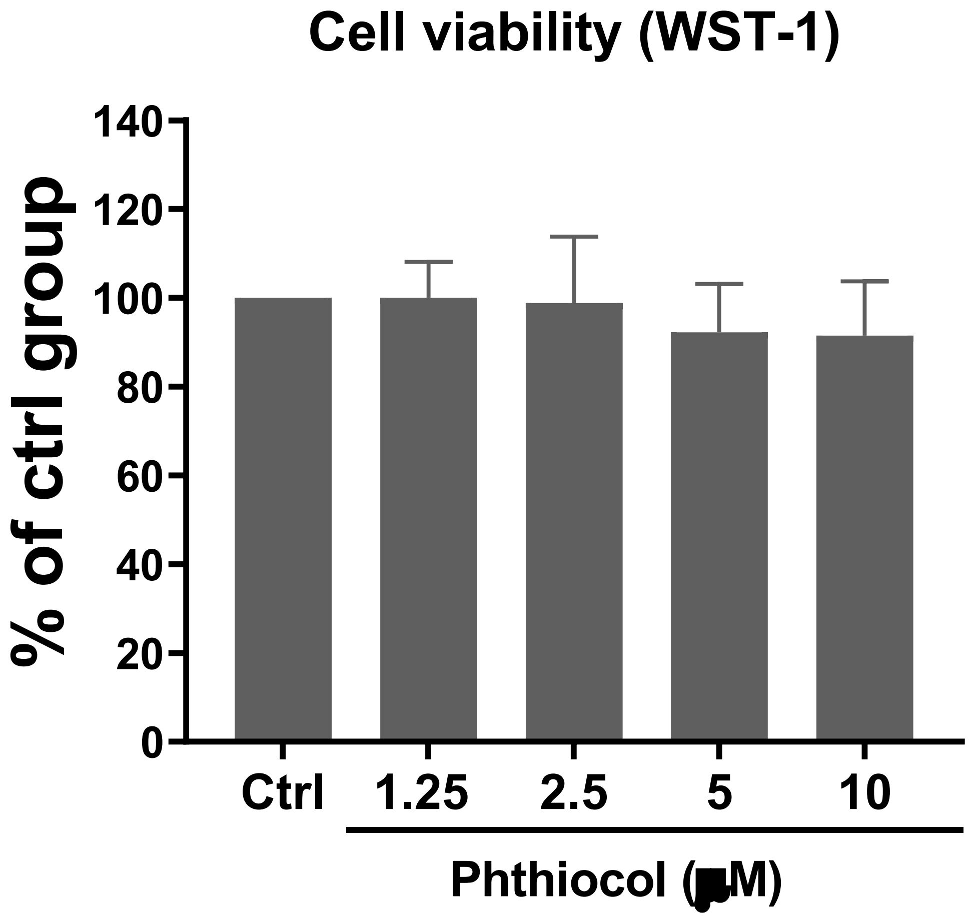

The comparisons between Raw Western blots and the Figures

Figure 3A

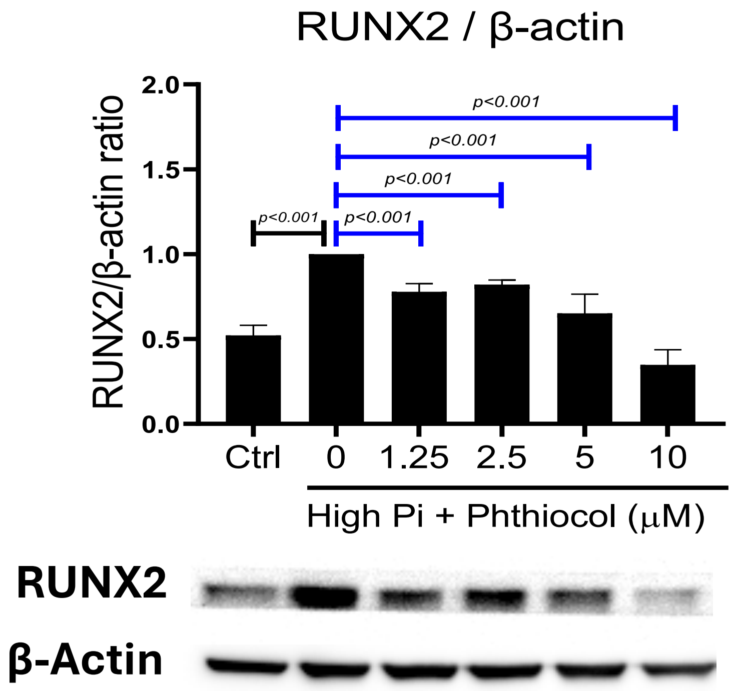
RUNX2 / β-actin
RUNX2/β-actin ratio
2.0
1.5
1.0
0.5
0.0
p<0.001
p<0.001
p<0.001
p<0.001
p<0.001
Ctrl
0
1.25
2.5
5
10
High Pi + Phthiocol (μM)
RUNX2
β-Actin

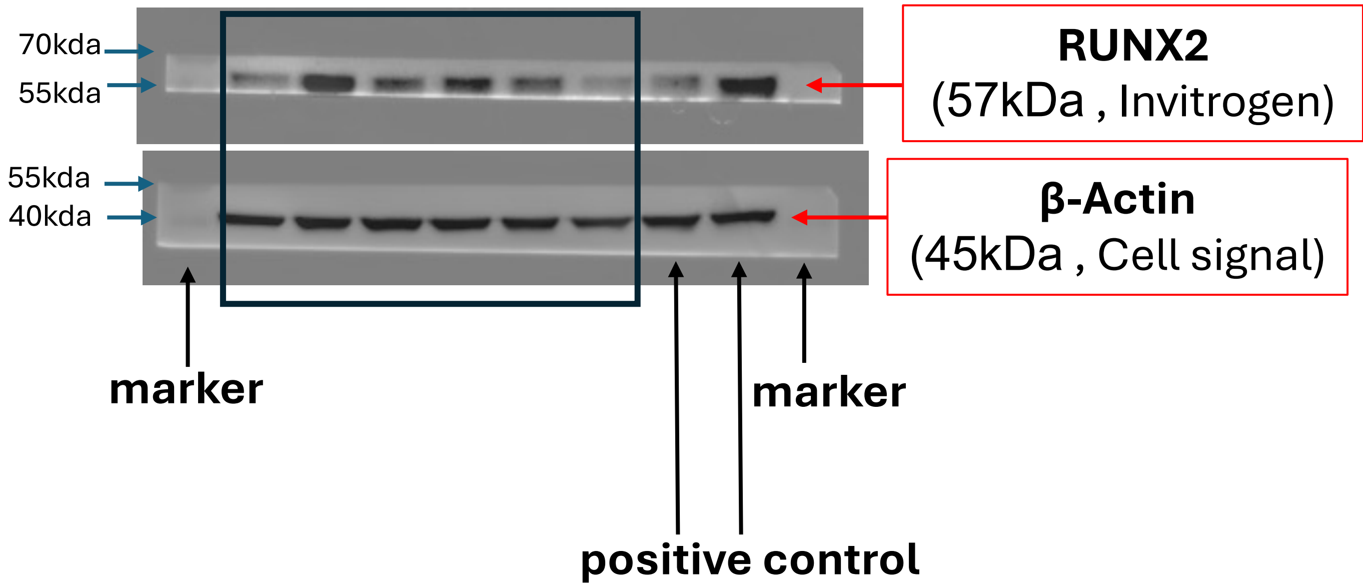
70kda
55kda
55kda
40kda
RUNX2
(57kDa , Invitrogen)
β-Actin
(45kDa , Cell signal)
marker
marker
positive control

raw data for Fig 3A

RUNX2
(57kDa , Invitrogen)

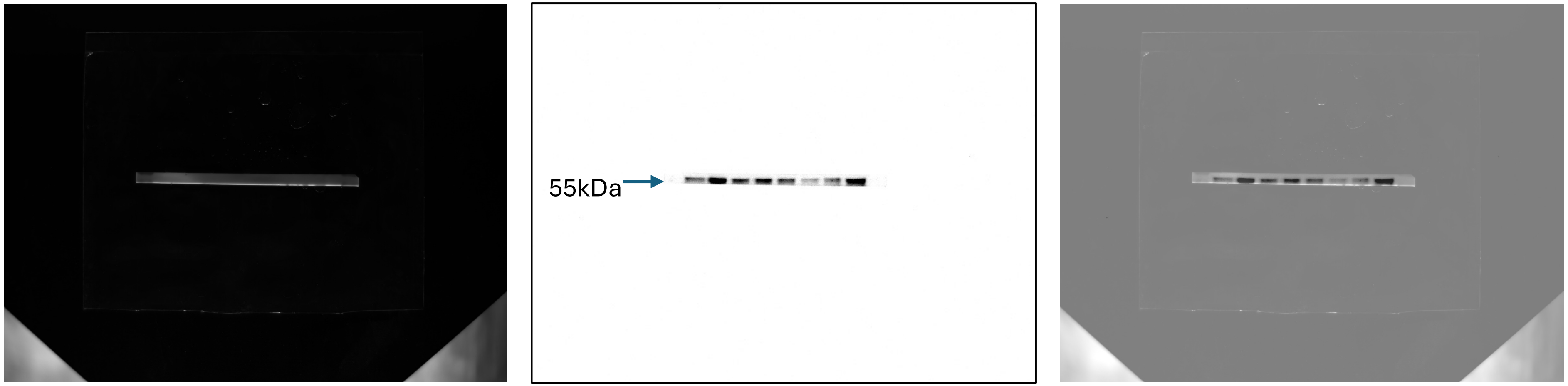

β-Actin
(45kDa , Cell signal)

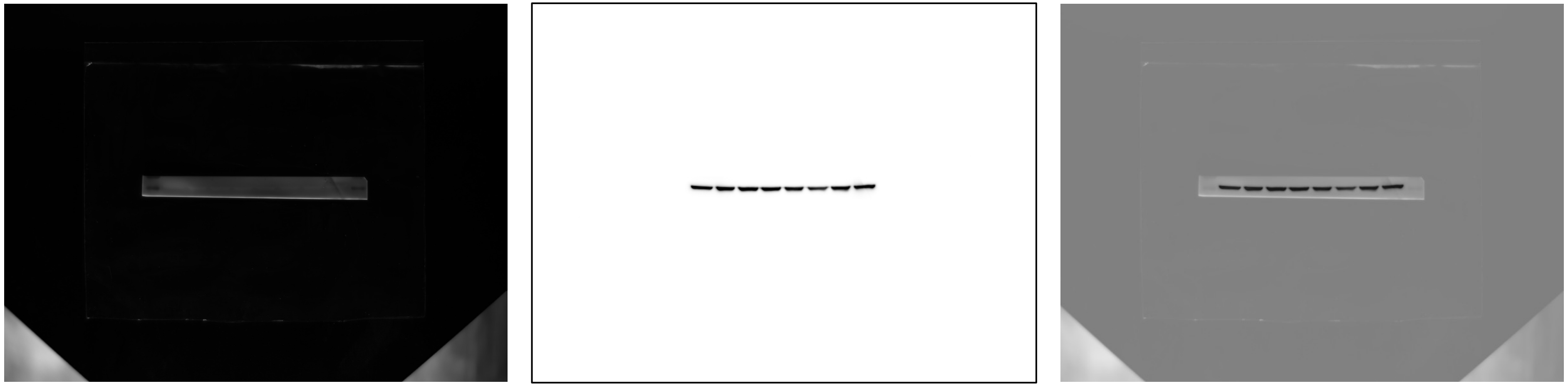

raw data for Fig 3A

RUNX2
(57kDa , Invitrogen)

β-Actin
(45kDa , Cell signal)

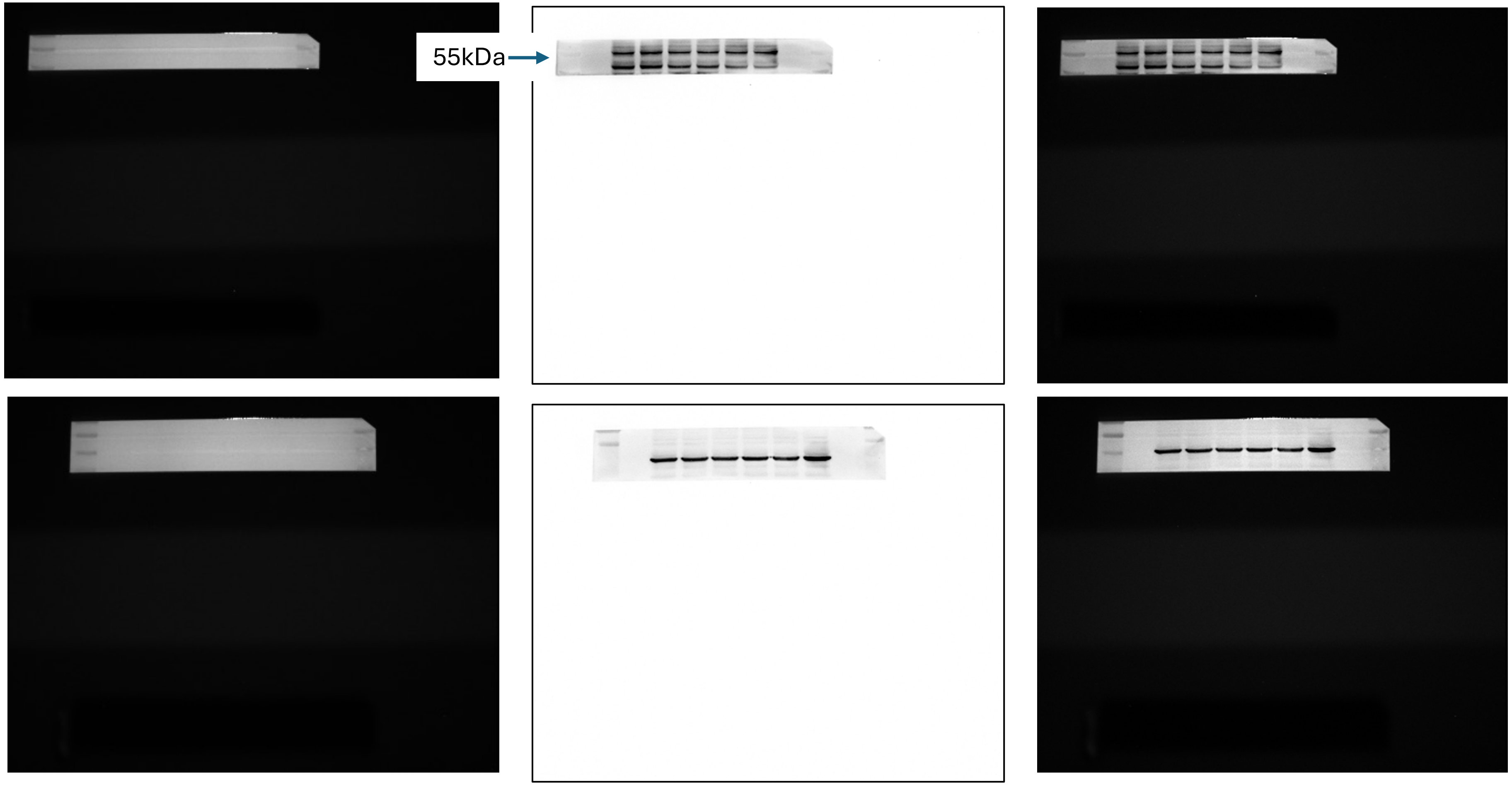

raw data for Fig 3A

RUNX2
(57kDa , Invitrogen)

β-Actin
(45kDa , Cell signal)

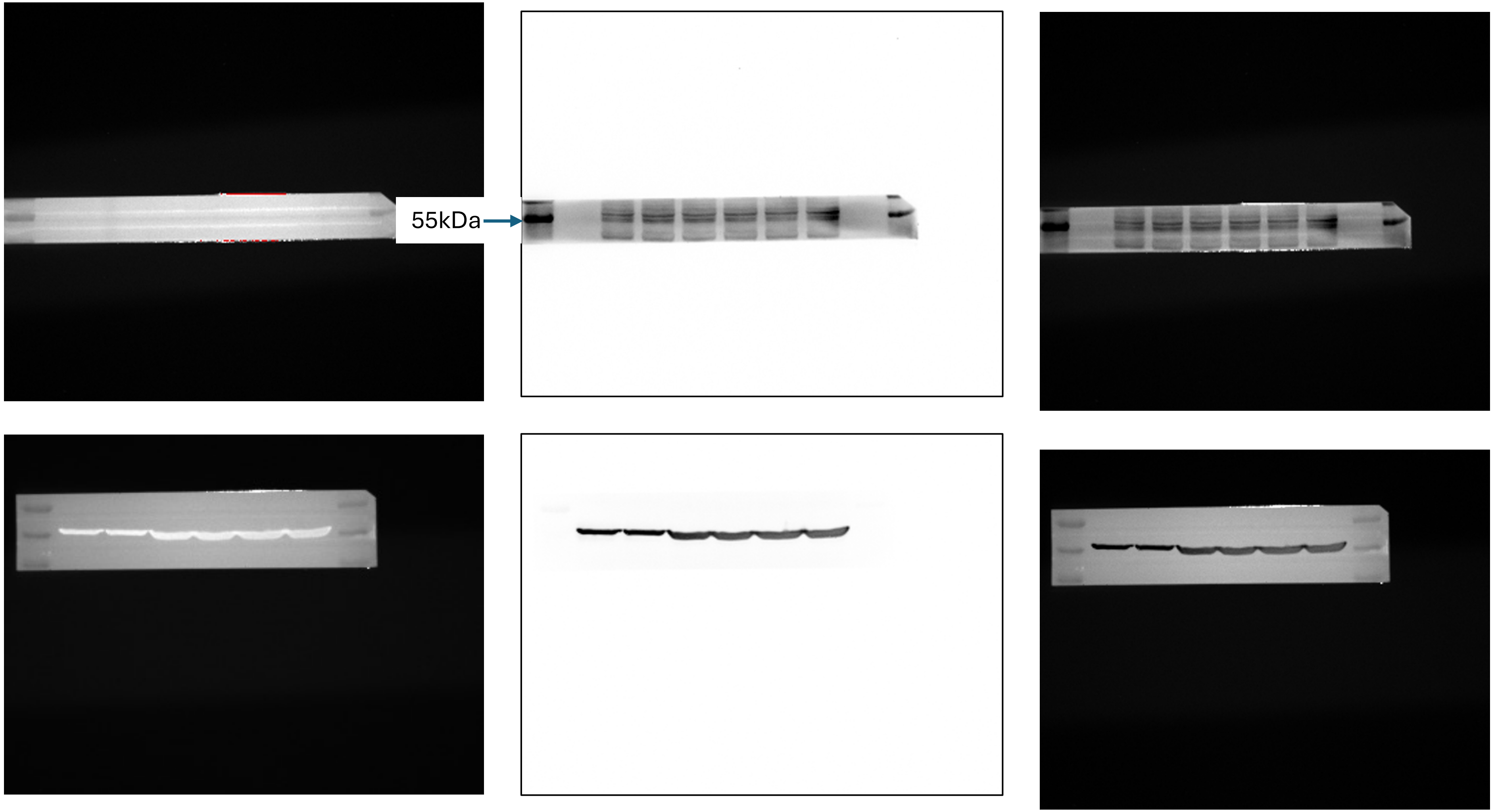

Figure 3B

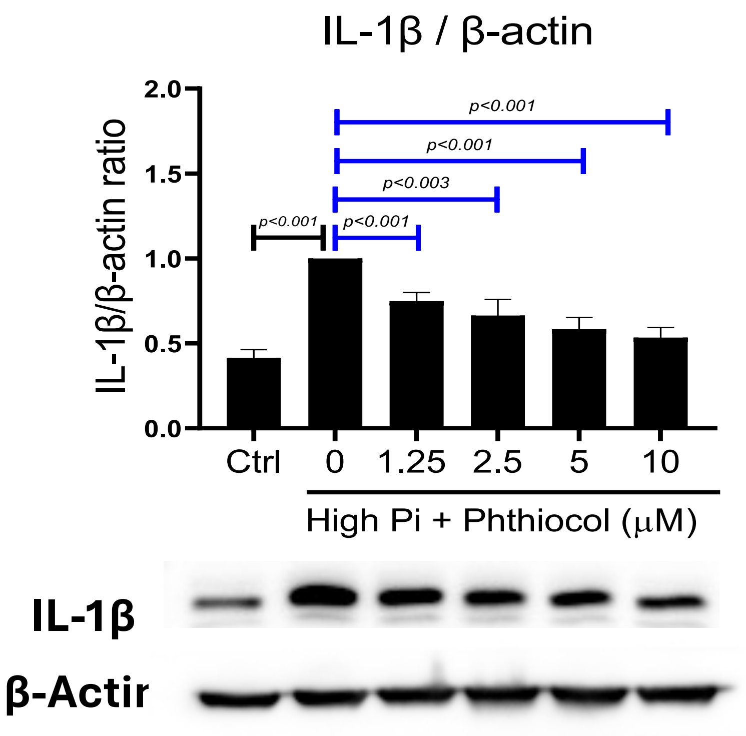
IL-1β / β-actin
IL-1β/β-actin ratio
2.0
1.5
1.0
0.5
0.0
p<0.001
p<0.001
p<0.003
p<0.001
p<0.001
Ctrl
0
1.25
2.5
5
10
High Pi + Phthiocol (μM)
IL-1β
β-Actir

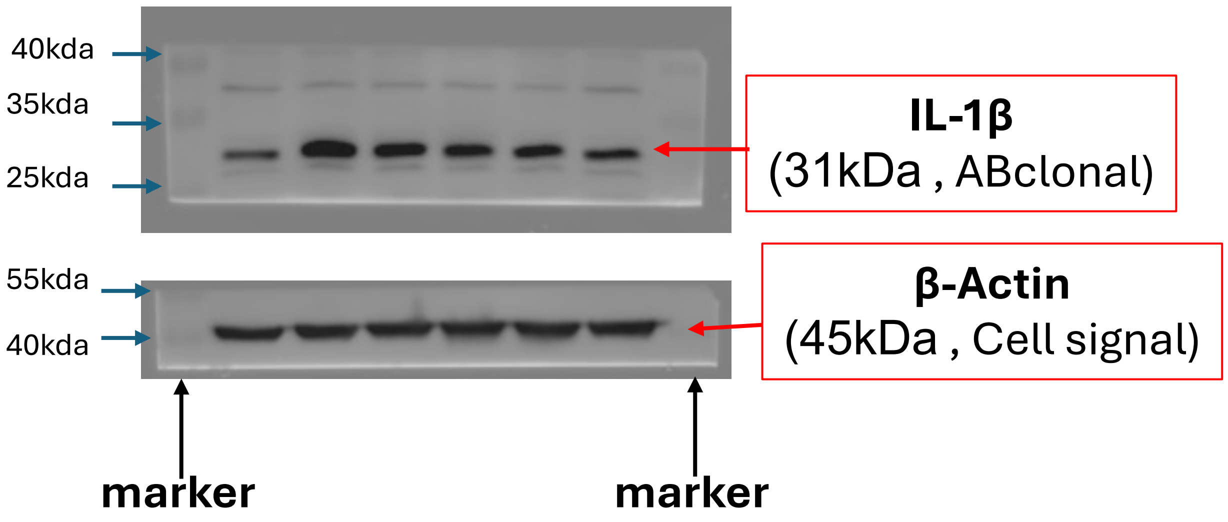
40kda
35kda
25kda
55kda
40kda
IL-1β
(31kDa , ABclonal)
β-Actin
(45kDa , Cell signal)
marker
marker

raw data for Figure 3B

IL-1β
(31kDa , ABclonal)

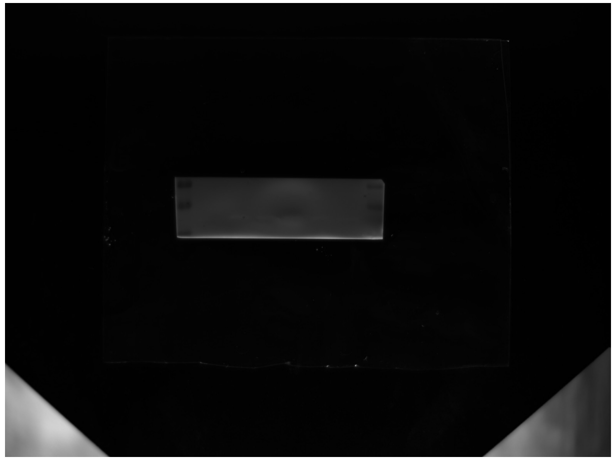

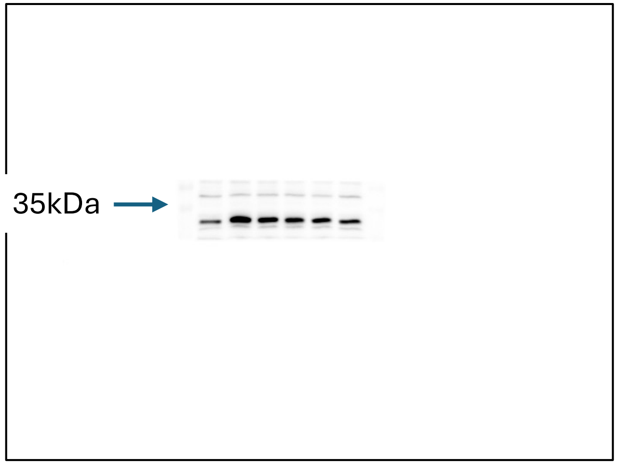

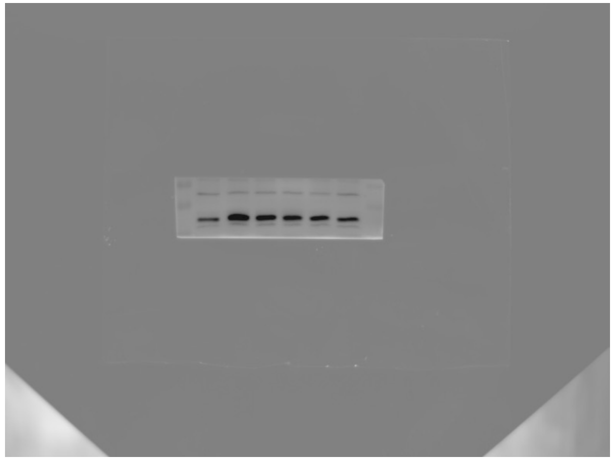

β-Actin
(45kDa , Cell signal)

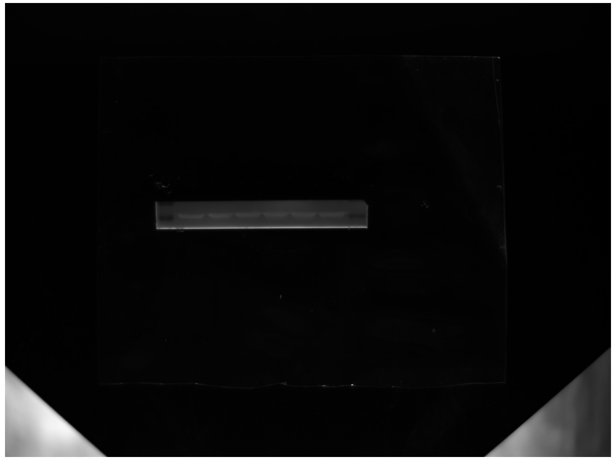

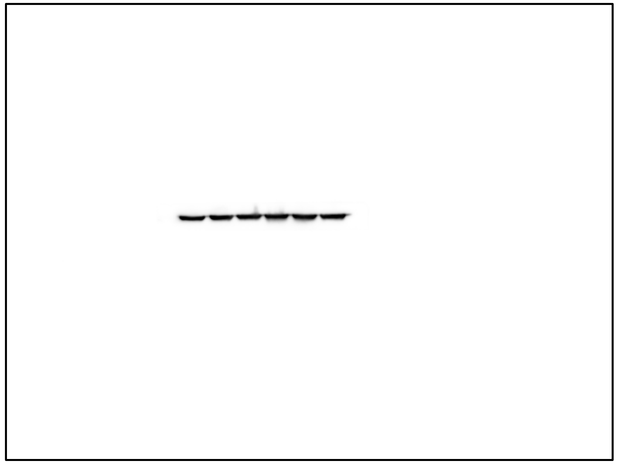

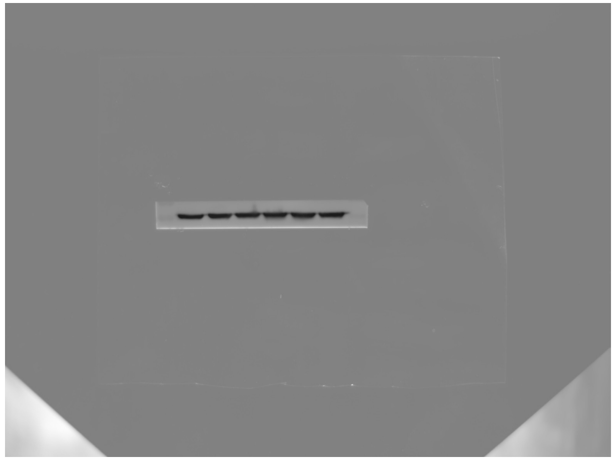

raw data for Figure 3B

IL-1β
(31kDa , ABclonal)

β-Actin
(45kDa , Cell signal)

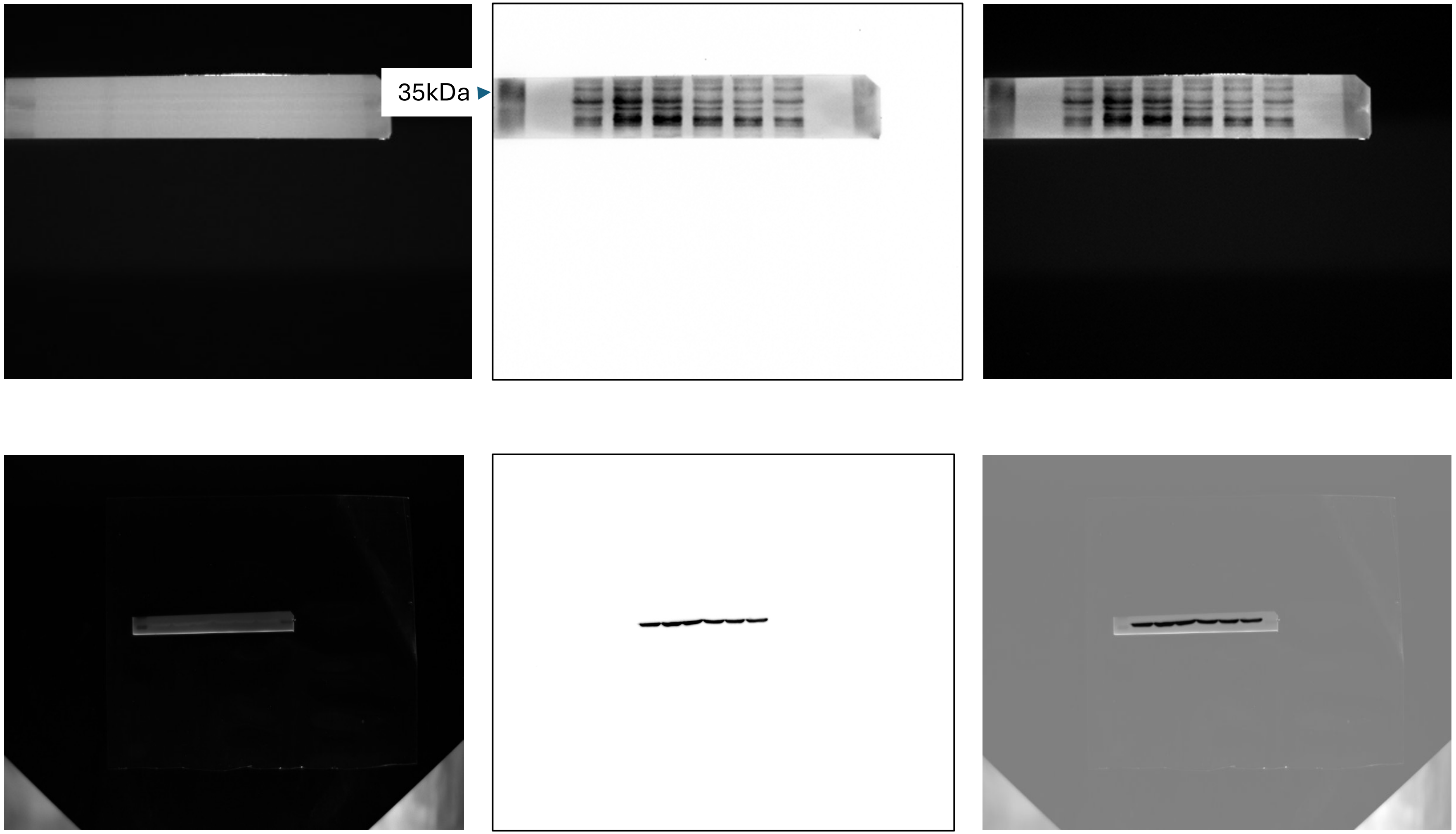

raw data for Figure 3B

IL-1β
(31kDa , ABclonal)

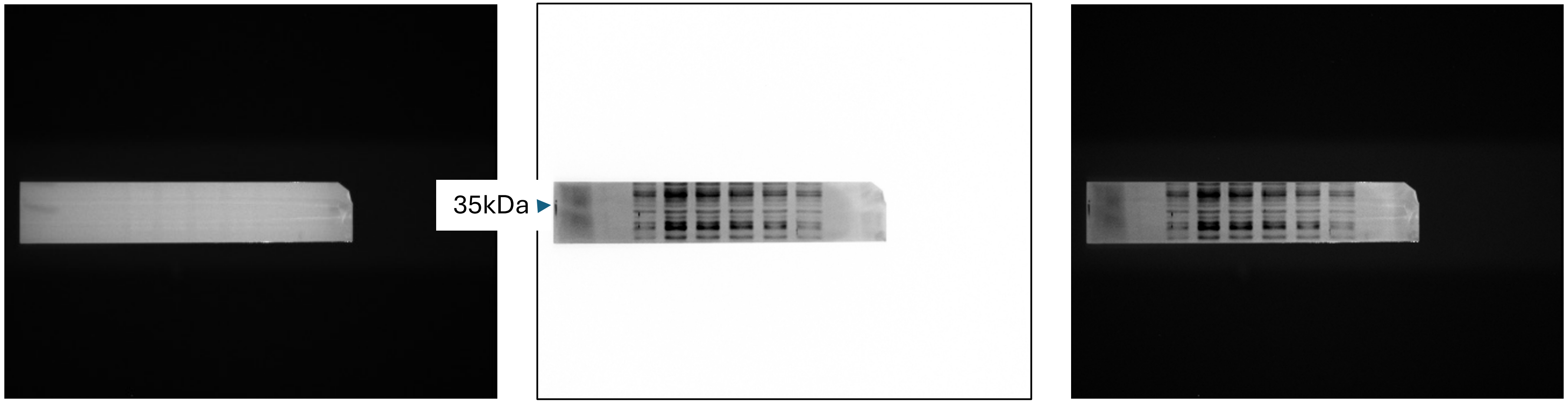

β-Actin
(45kDa , Cell signal)

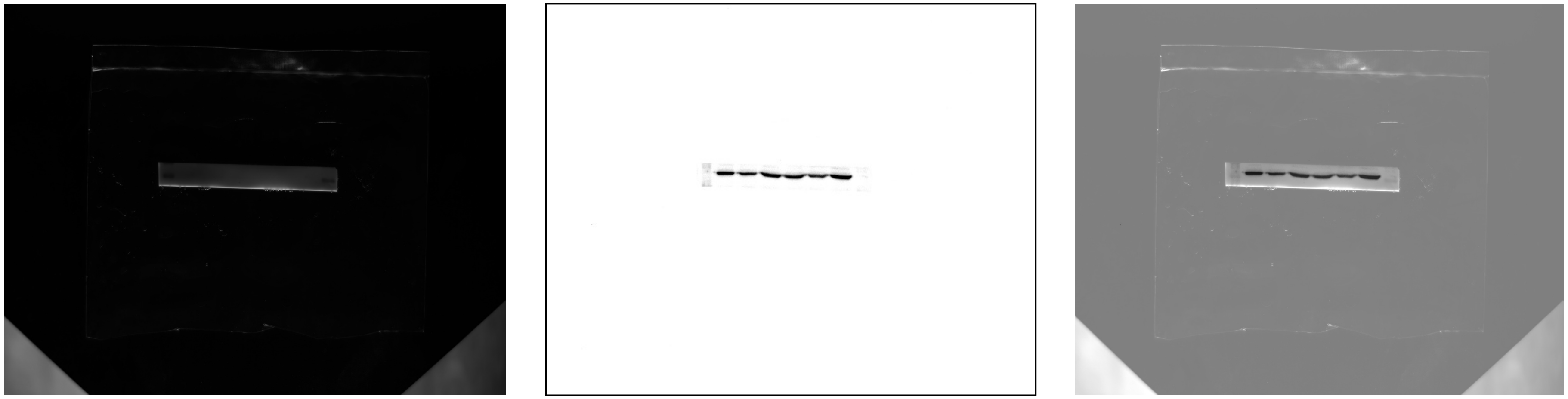

Figure 3C

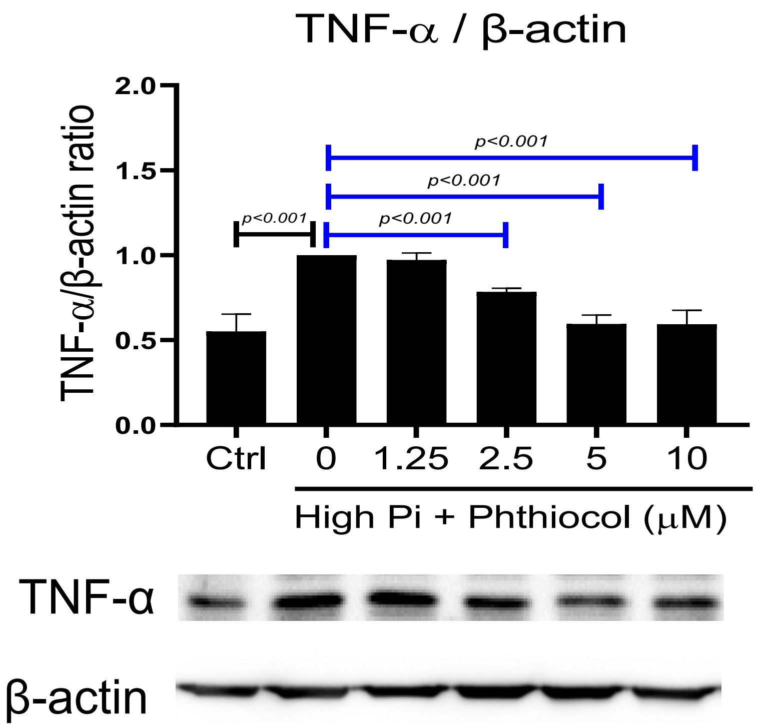
TNF-α / β-actin
TNF-α/β-actin ratio
2.0
1.5
1.0
0.5
0.0
p<0.001
p<0.001
p<0.001
p<0.001
Ctrl
0
1.25
2.5
5
10
High Pi + Phthiocol (μM)
TNF-α
β-actin

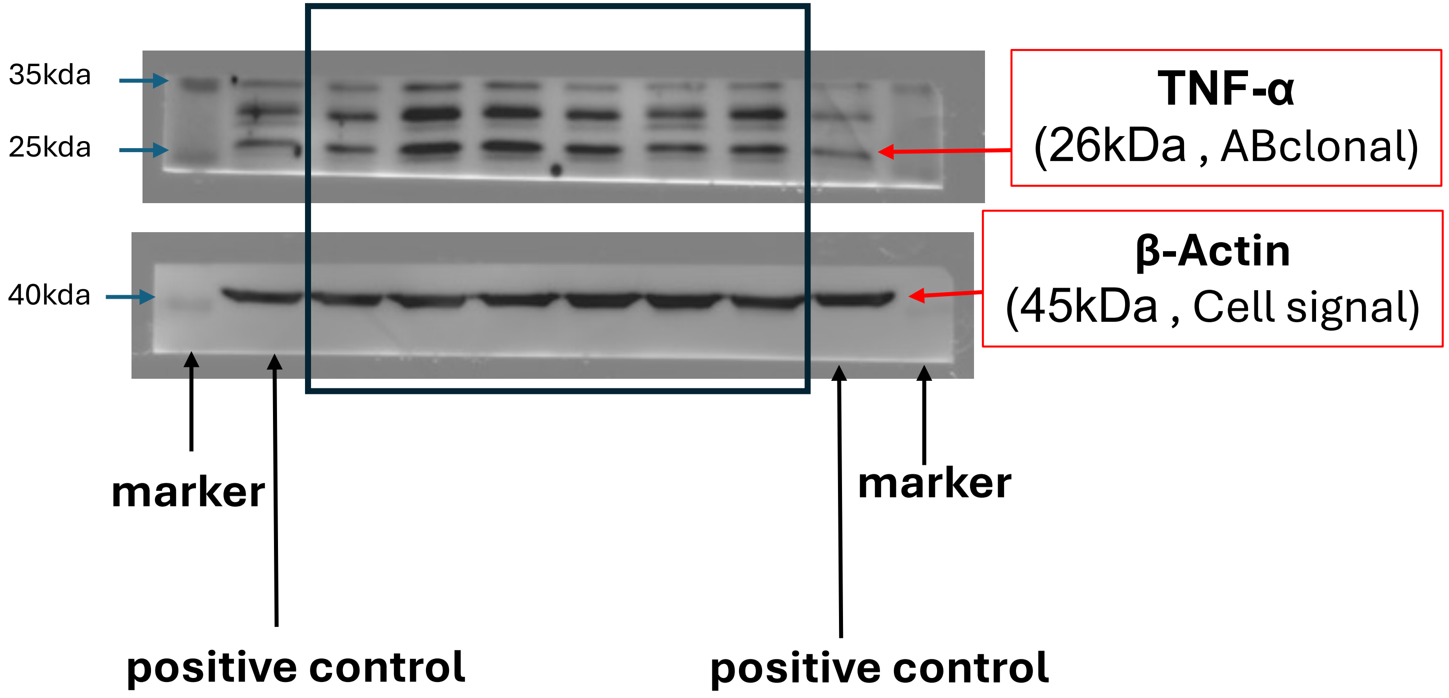
35kda
25kda
40kda
TNF-α
(26kDa , ABclonal)
β-Actin
(45kDa , Cell signal)
marker
marker
positive control
positive control

raw data for Figure 3C

TNF-α
(26kDa , ABclonal)

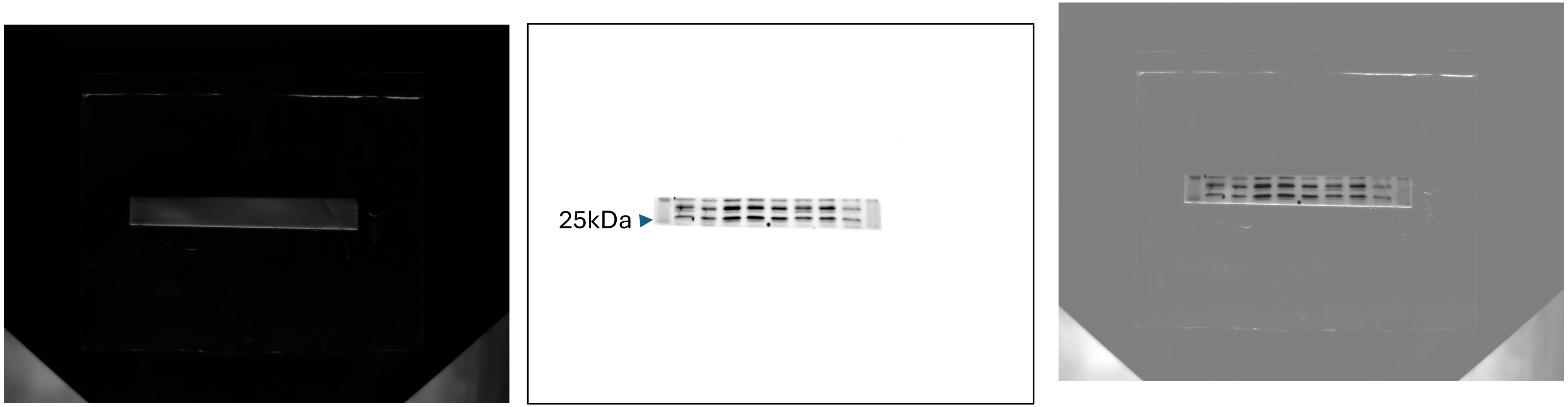

β-Actin
(45kDa , Cell signal)

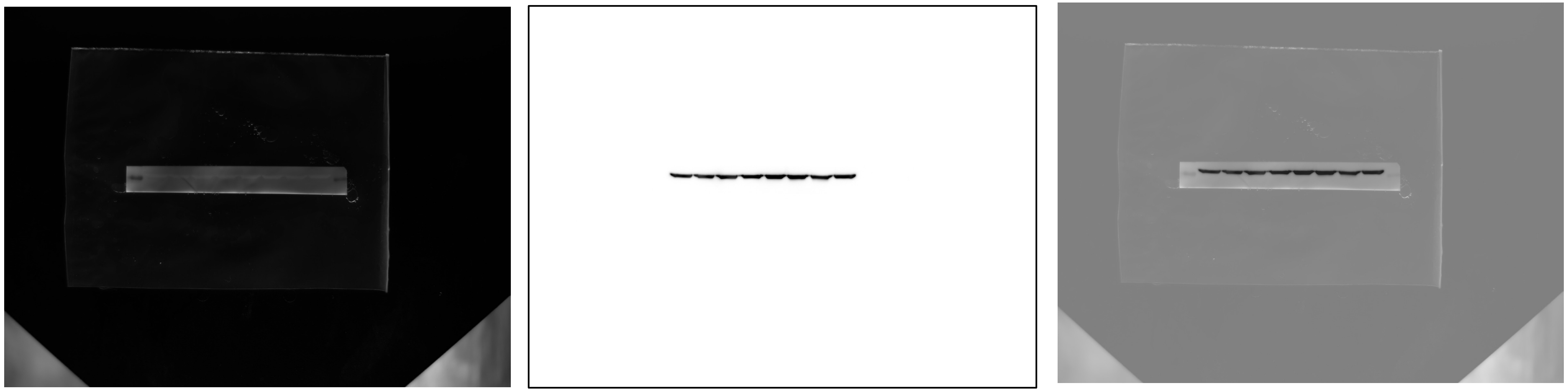

raw data for Figure 3C

TNF-α
(26kDa , ABclonal)

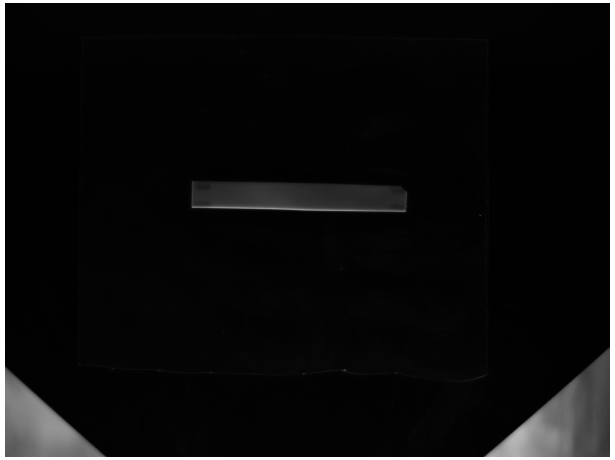

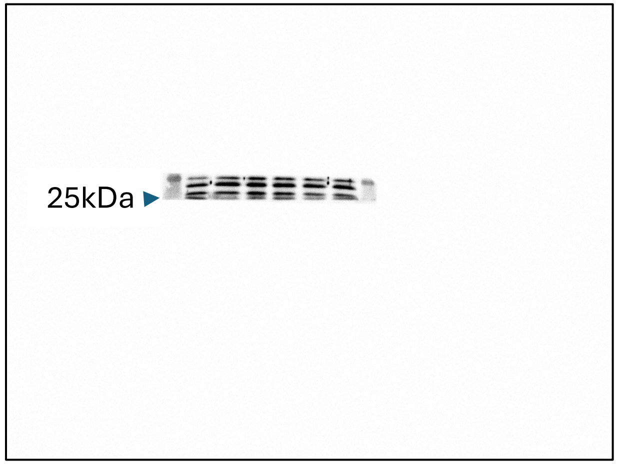

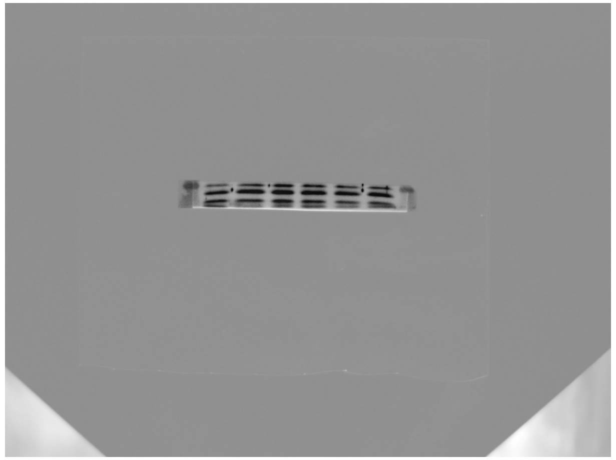

β-Actin
(45kDa , Cell signal)

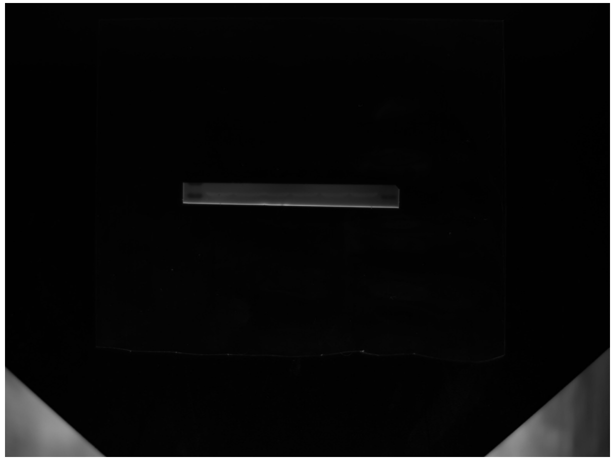

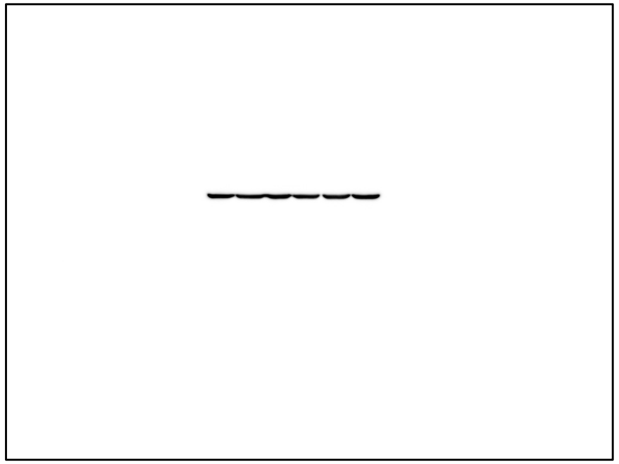

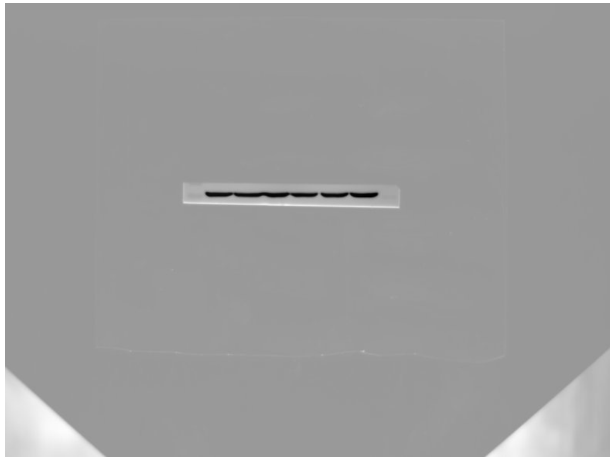

raw data for Figure 3C

TNF-α
(26kDa , ABclonal)

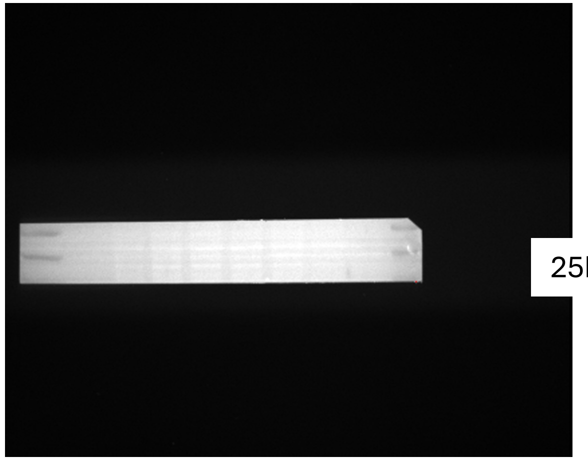

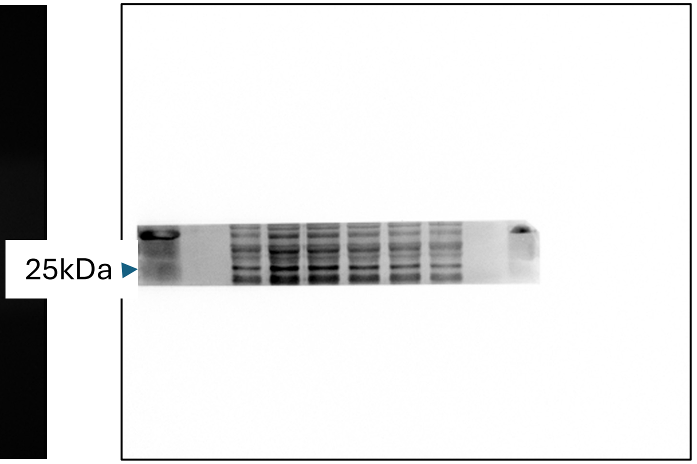

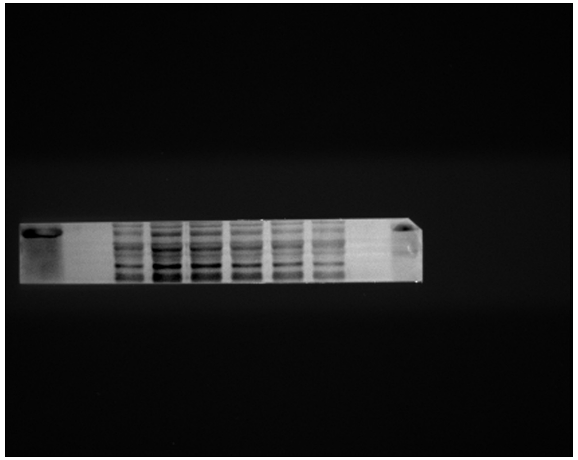

β-Actin
(45kDa , Cell signal)

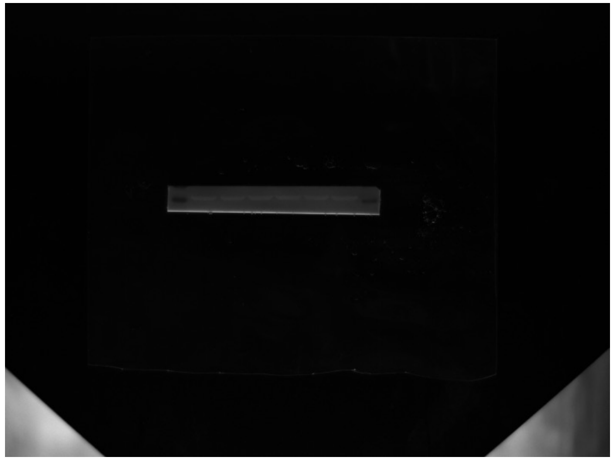

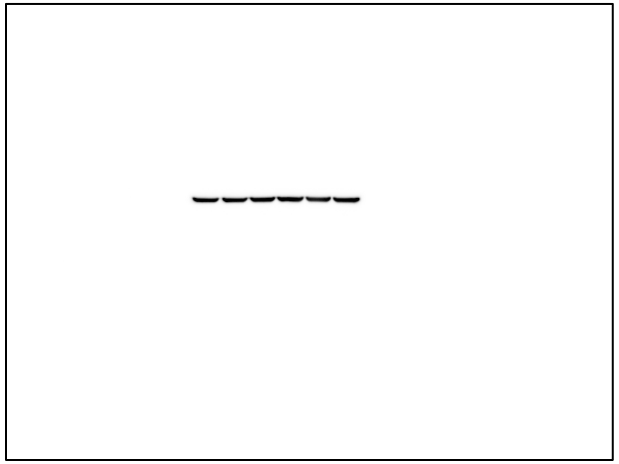

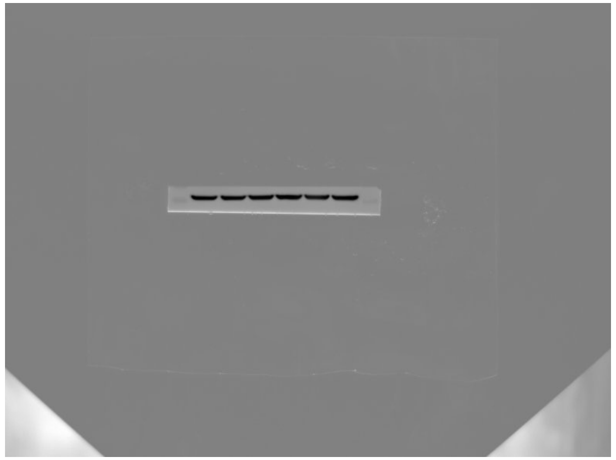

Figure 3D

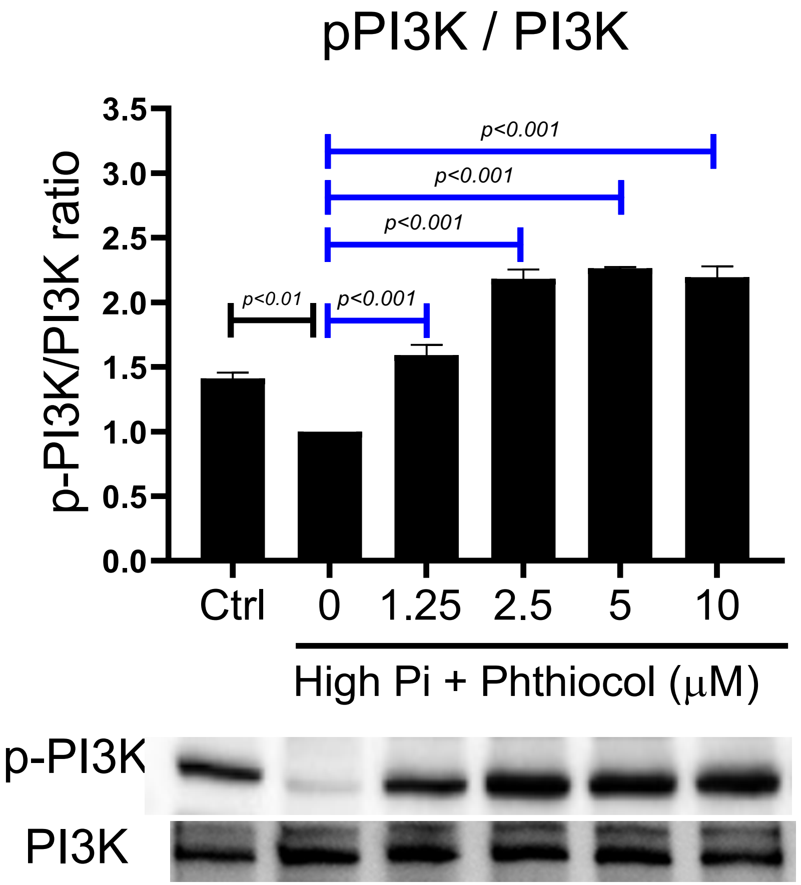

pPI3K / PI3K
p-PI3K/PI3K ratio
3.5
3.0
2.5
2.0
1.5
1.0
0.5
0.0
p<0.001
p<0.001
p<0.001
p<0.01
p<0.001
Ctrl
0
1.25
2.5
5
10
High Pi + Phthiocol (µM)
p-PI3K
PI3K

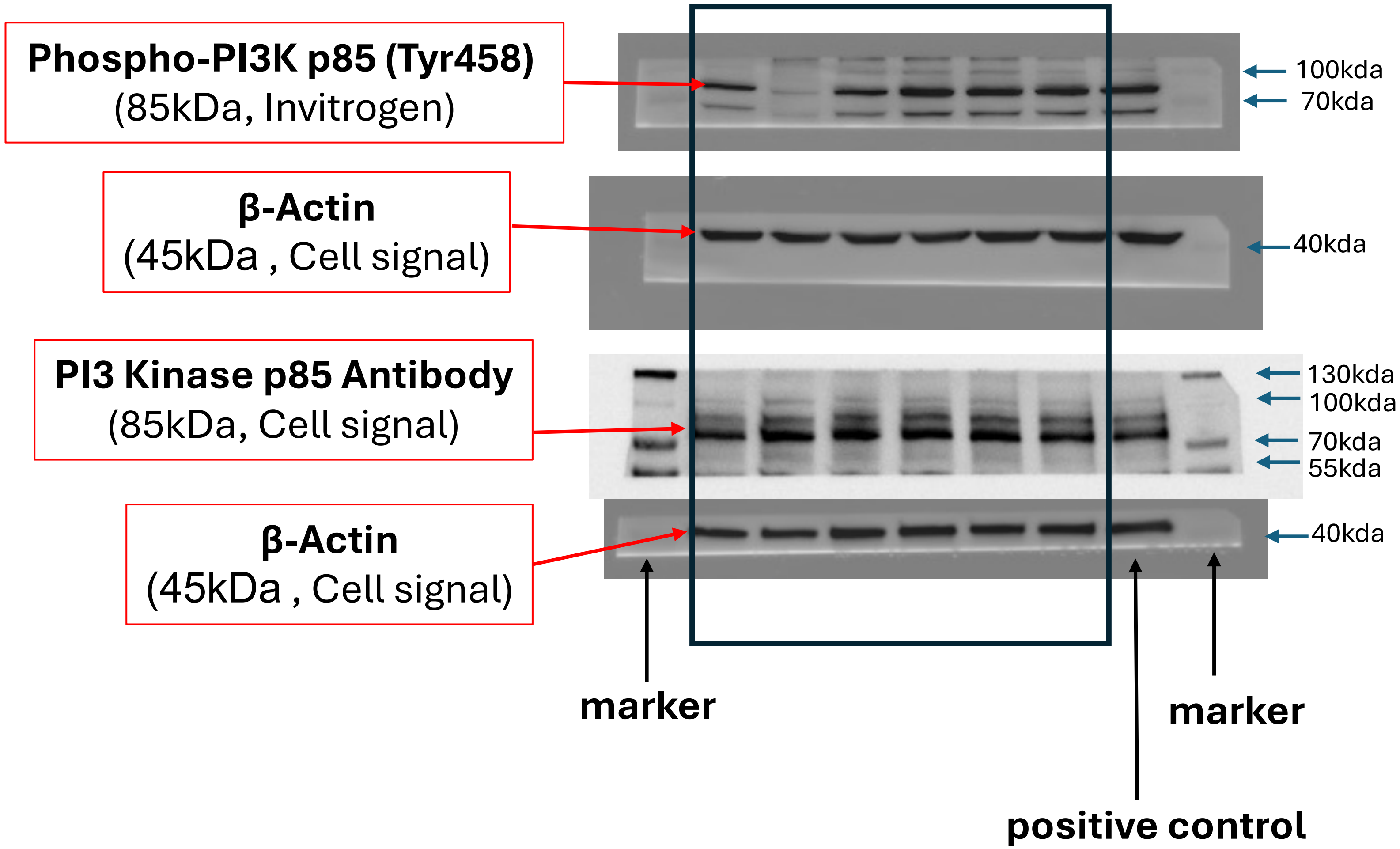

Phospho-PI3K p85 (Tyr458)
(85kDa, Invitrogen)
β-Actin
(45kDa , Cell signal)
PI3 Kinase p85 Antibody
(85kDa, Cell signal)
β-Actin
(45kDa , Cell signal)
100kda
70kda
40kda
130kda
100kda
70kda
55kda
40kda
marker
marker
positive control

raw data for Figure 3D

Phospho-PI3K p85 (Tyr458)
(85kDa, Invitrogen)

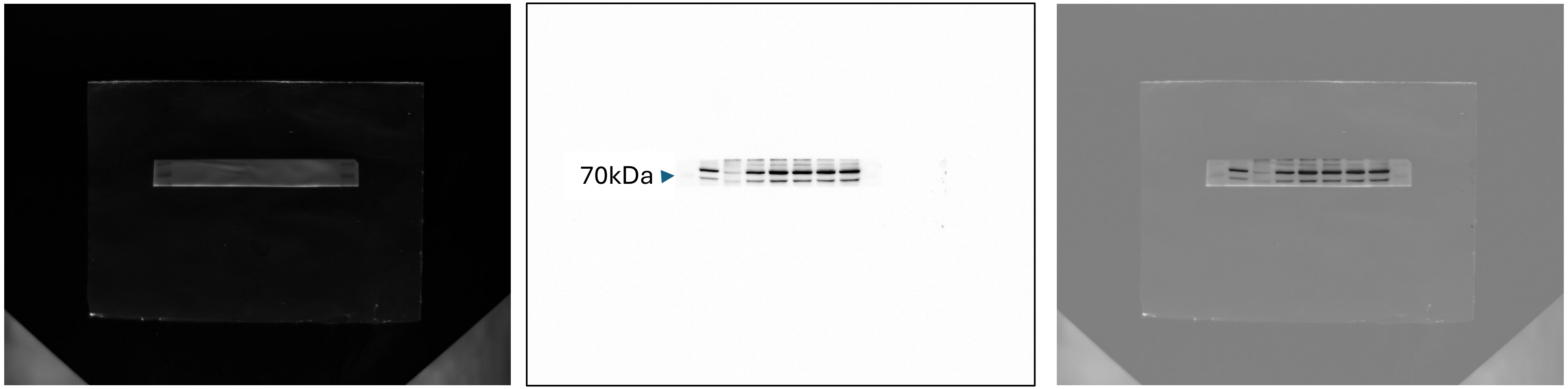

β-Actin
(45kDa , Cell signal)

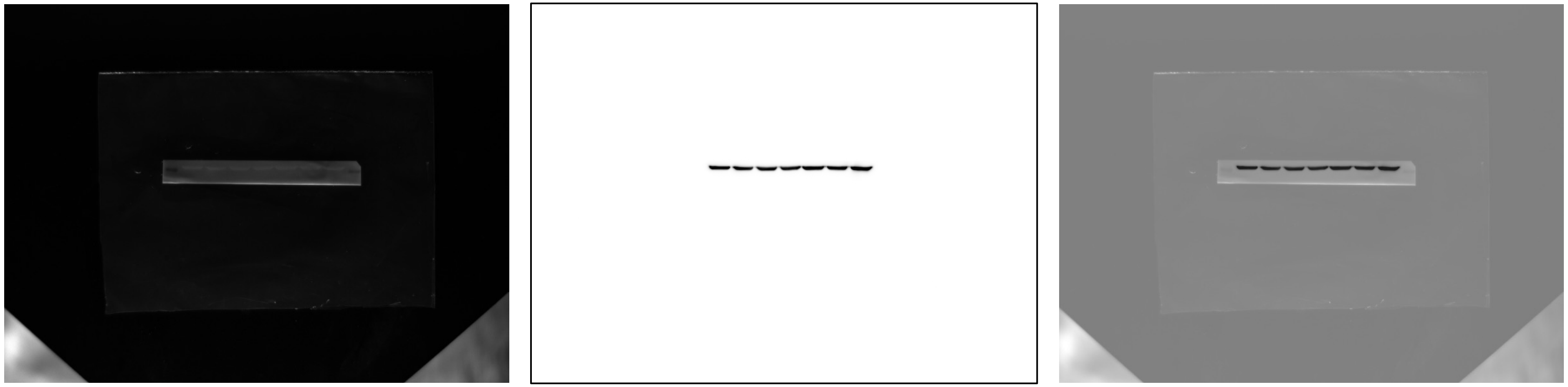

raw data for Figure 3D

PI3 Kinase p85 Antibody
(85kDa, Cell signal)

β-Actin
(45kDa , Cell signal)

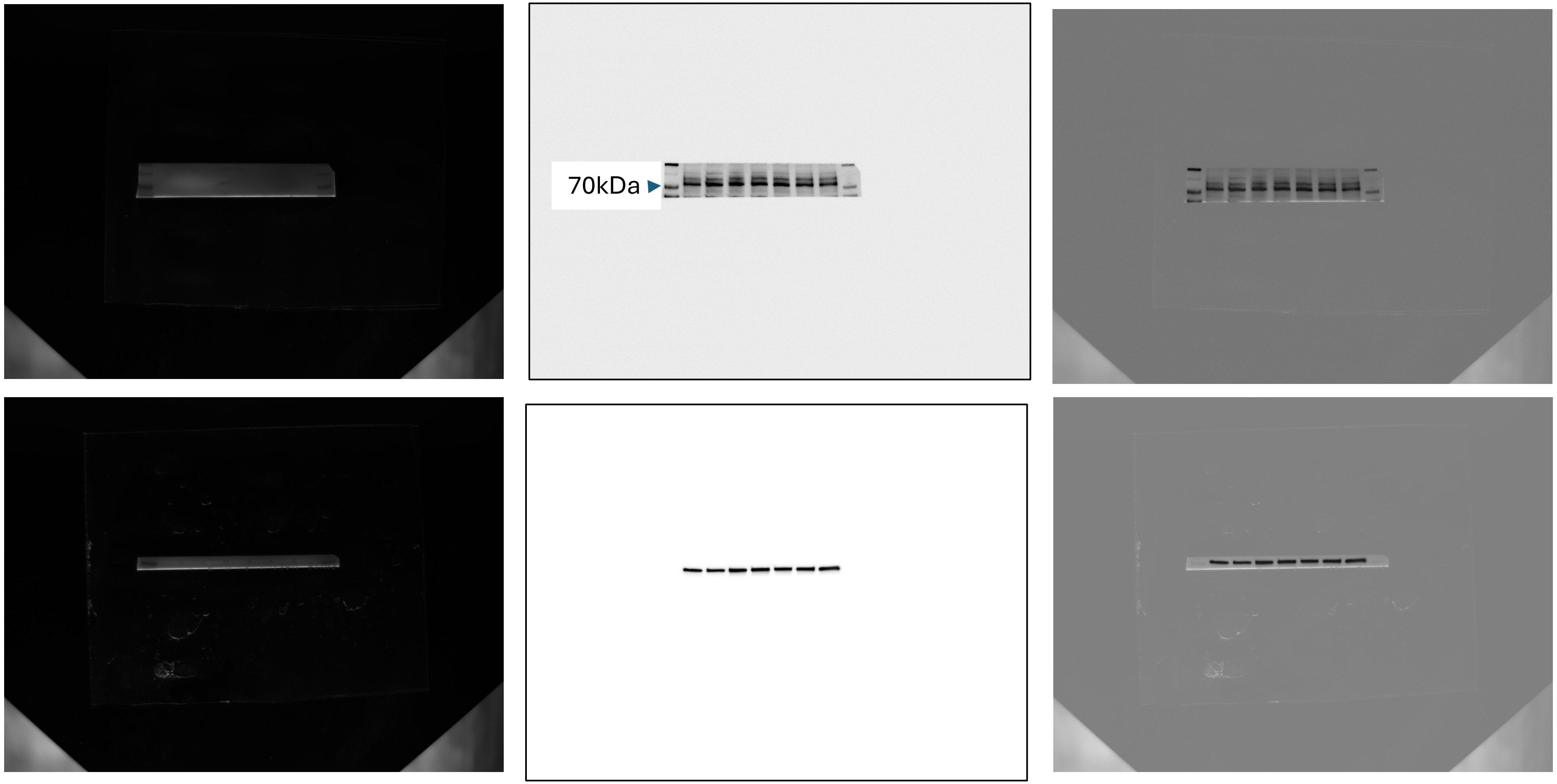

raw data for Figure 3D

Phospho-PI3K p85 (Tyr458)
(85kDa, Invitrogen)

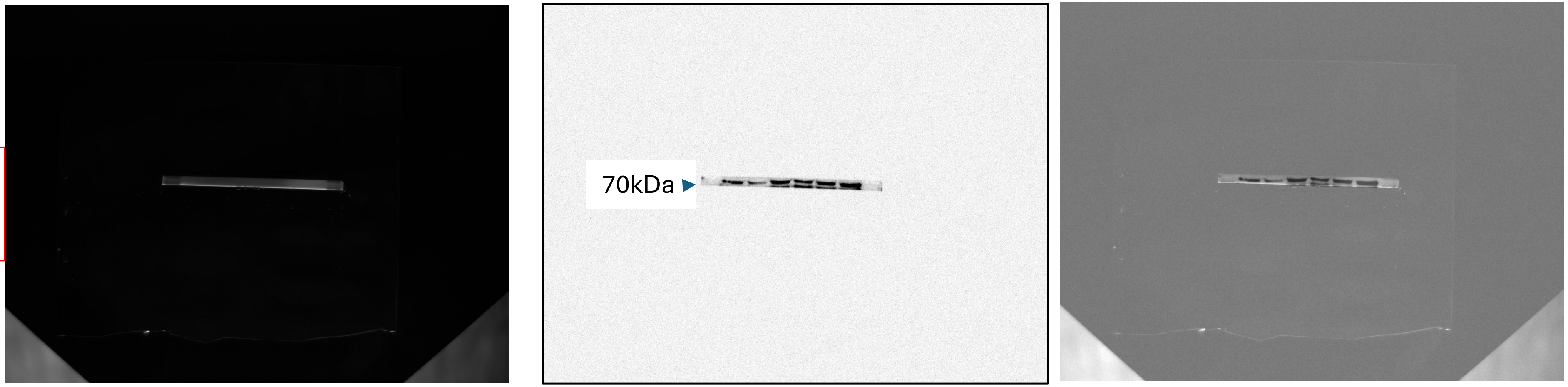

β-Actin
(45kDa , Cell signal)

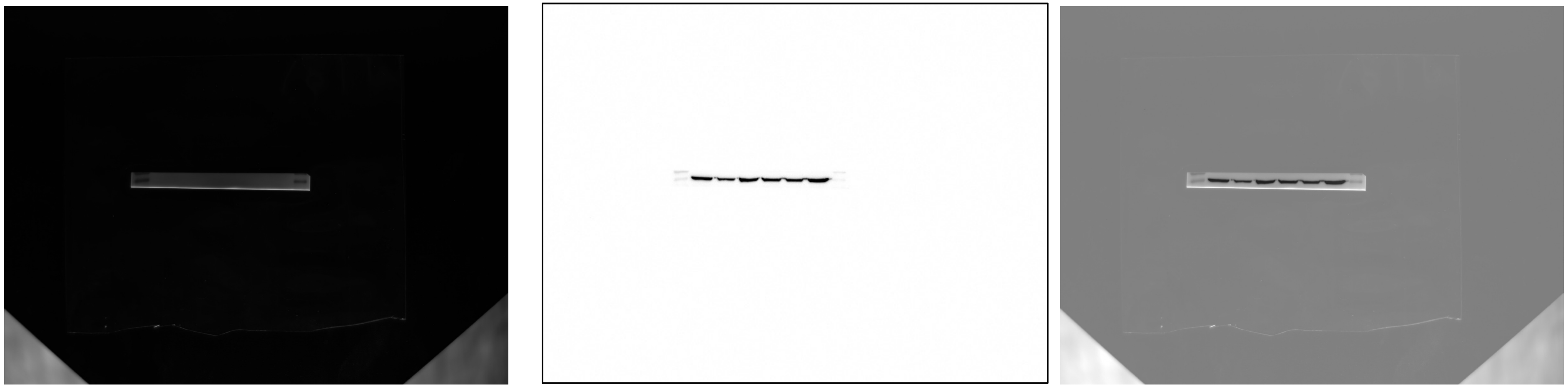

raw data for Figure 3D

Phospho-PI3K p85 (Tyr458)
(85kDa, Invitrogen)

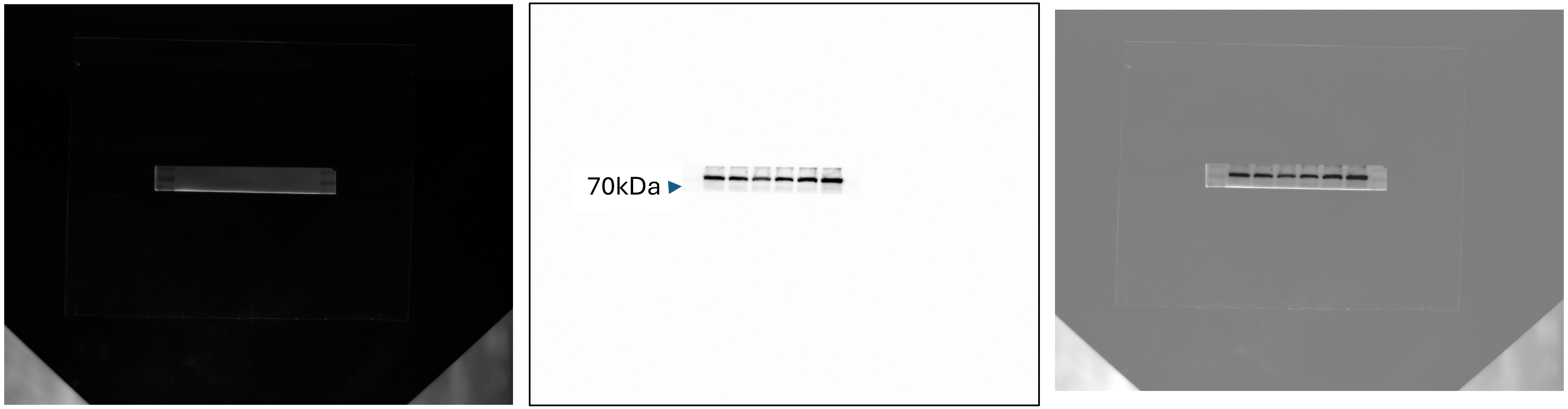

β-Actin
(45kDa , Cell signal)

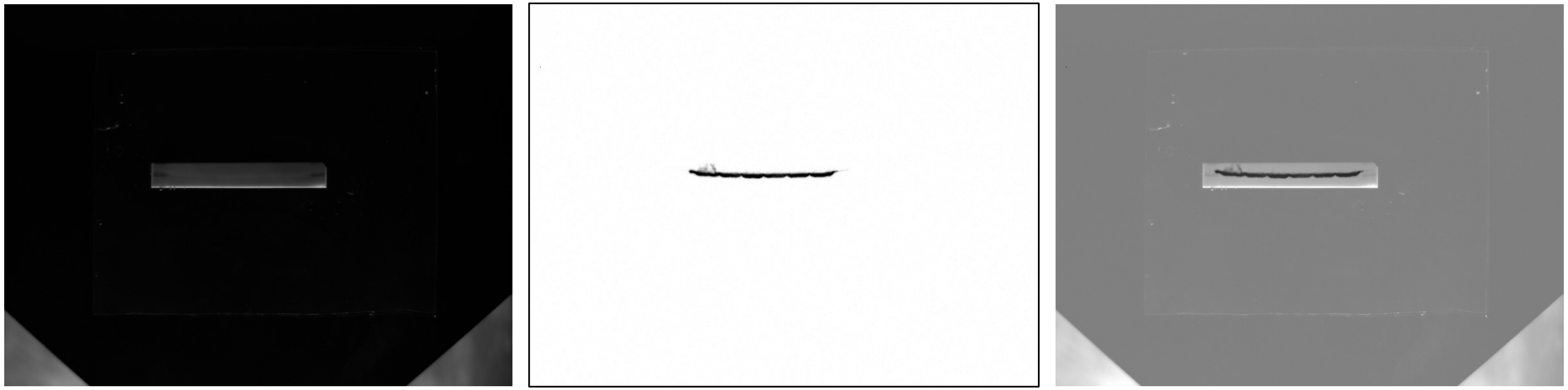

raw data for Figure 3D

PI3 Kinase p85 Antibody
(85kDa, Cell signal)

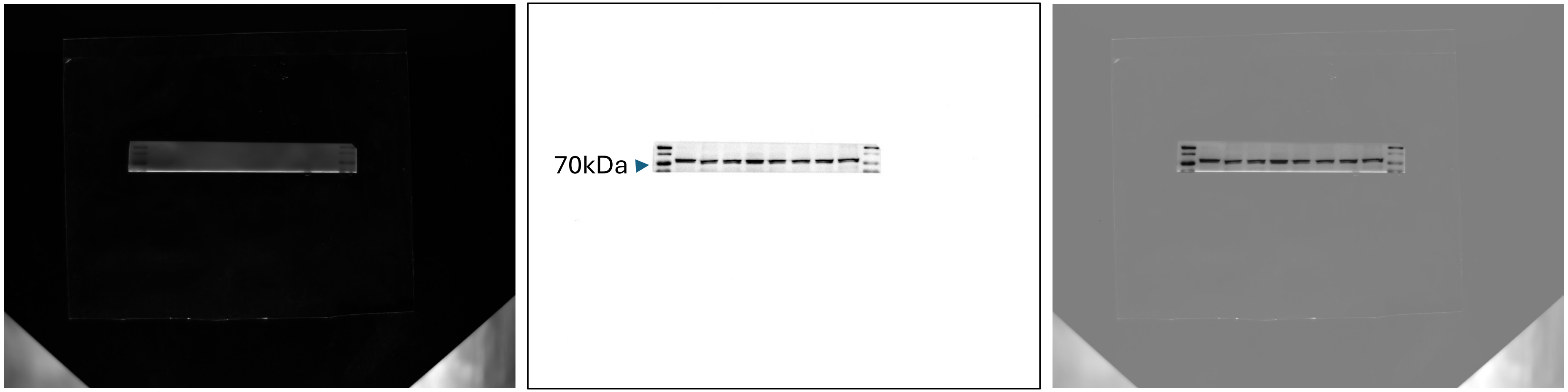

β-Actin
(45kDa , Cell signal)

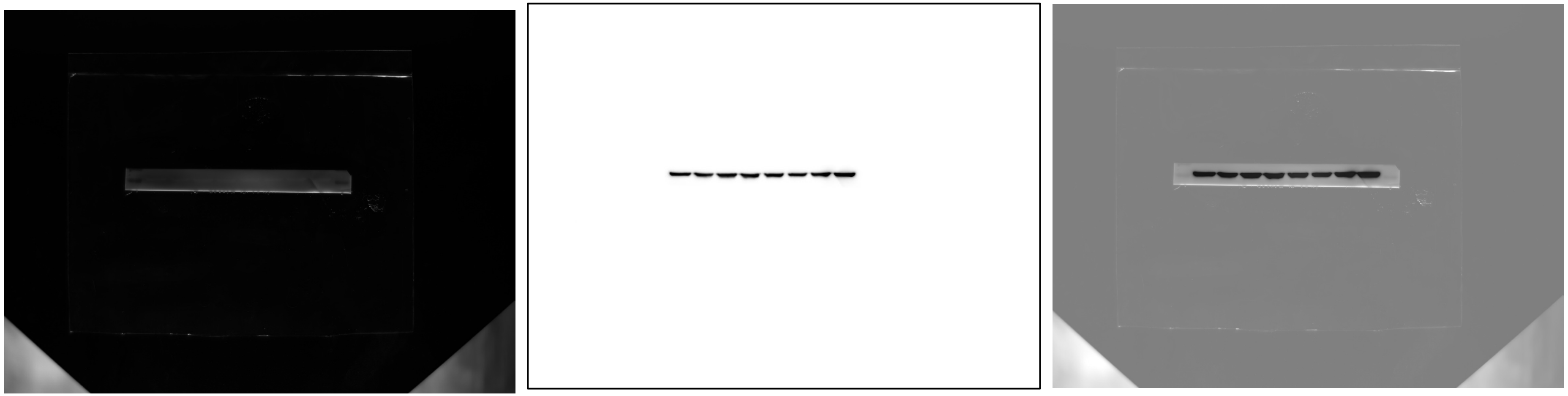

raw data for Figure 3D

PI3 Kinase p85 Antibody
(85kDa, Cell signal)

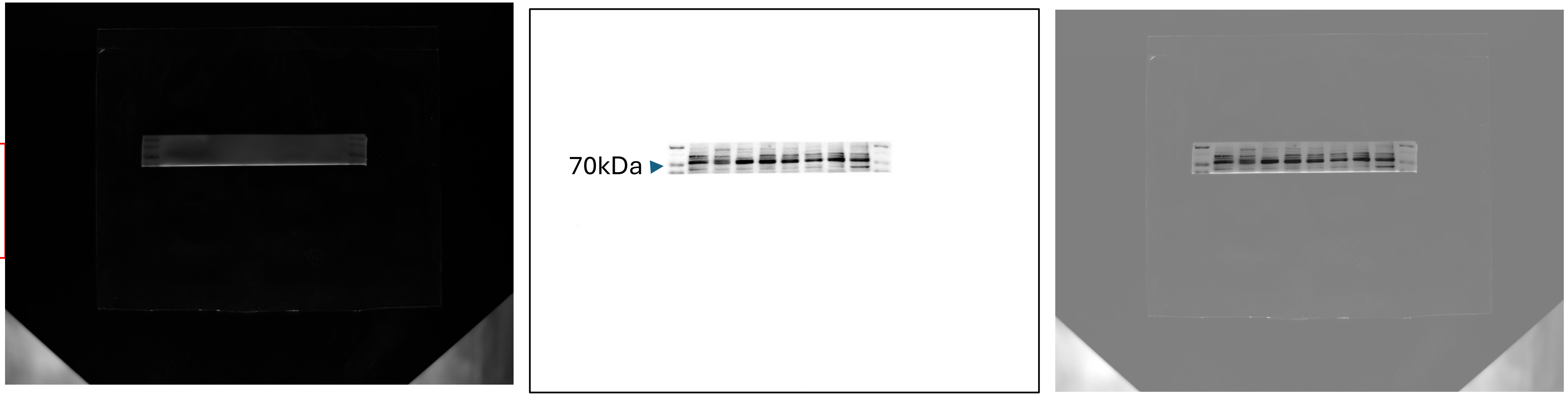

β-Actin
(45kDa , Cell signal)

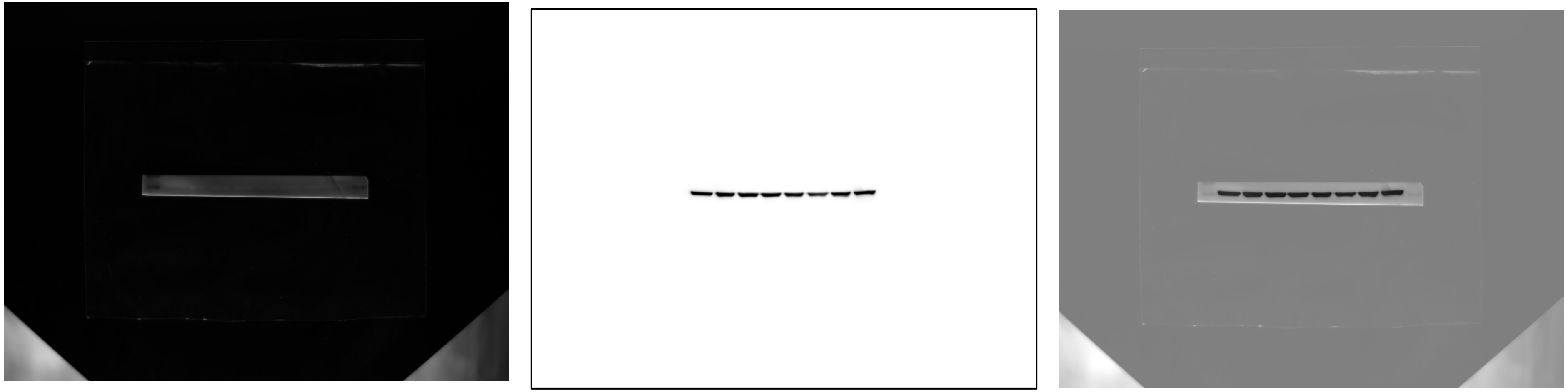

Figure 3E

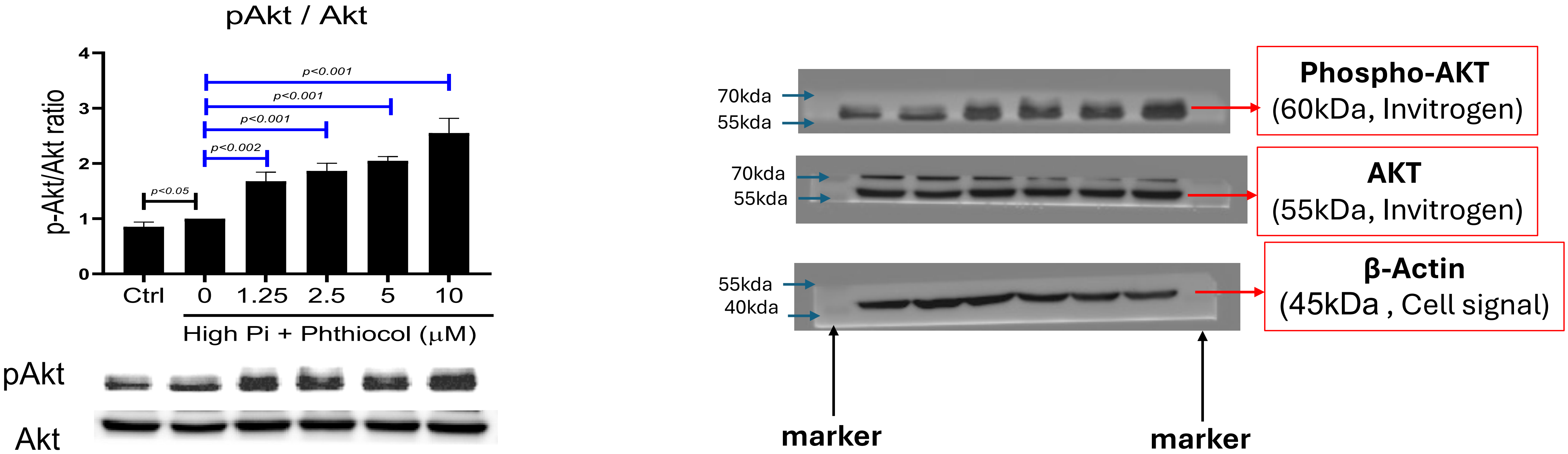
pAkt / Akt
p-Akt/Akt ratio
0
1
2
3
4
p<0.05
p<0.002
p<0.001
p<0.001
p<0.001
Ctrl
0
1.25
2.5
5
10
High Pi + Phthiocol (μM)
pAkt
Akt
70kda
55kda
Phospho-AKT
(60kDa, Invitrogen)
70kda
55kda
AKT
(55kDa, Invitrogen)
55kda
40kda
β-Actin
(45kDa , Cell signal)
marker
marker

raw data for Figure 3E

Phospho-AKT
(60kDa, Invitrogen)

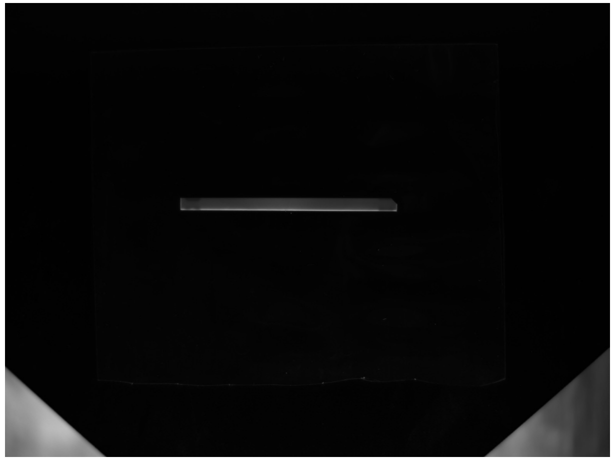

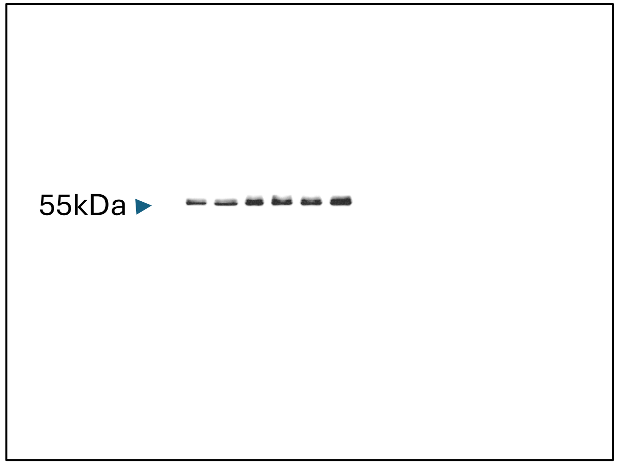

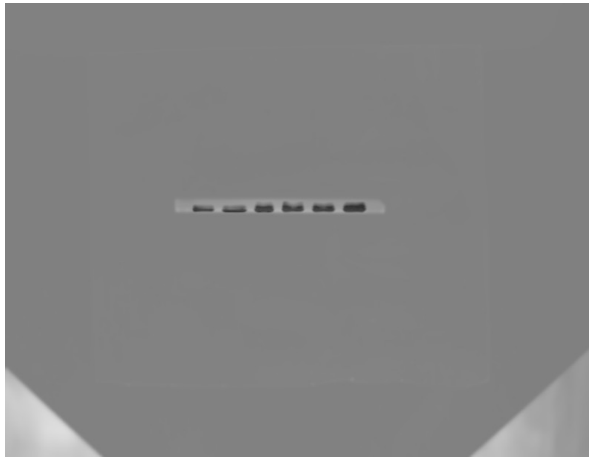

β-Actin
(45kDa , Cell signal)

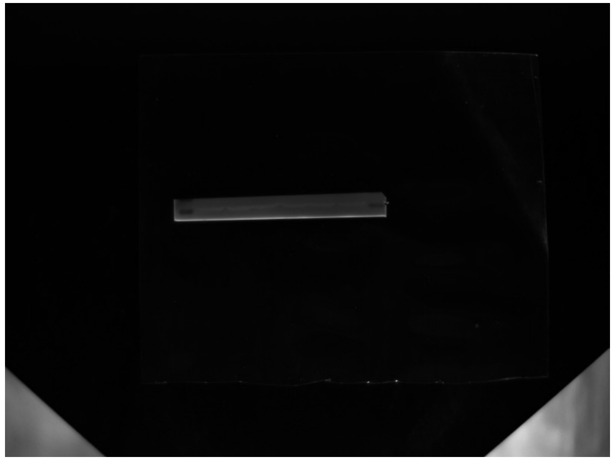

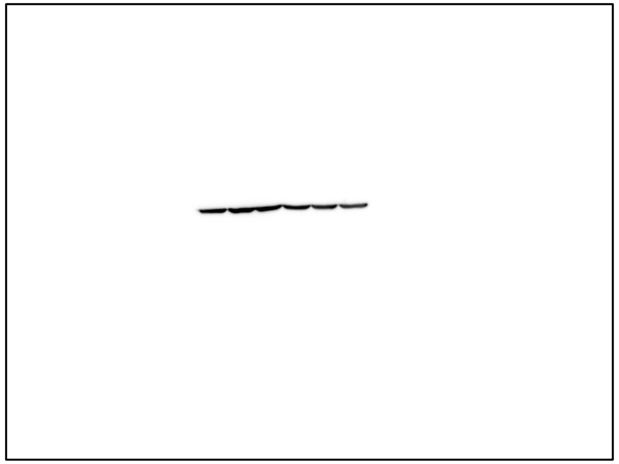

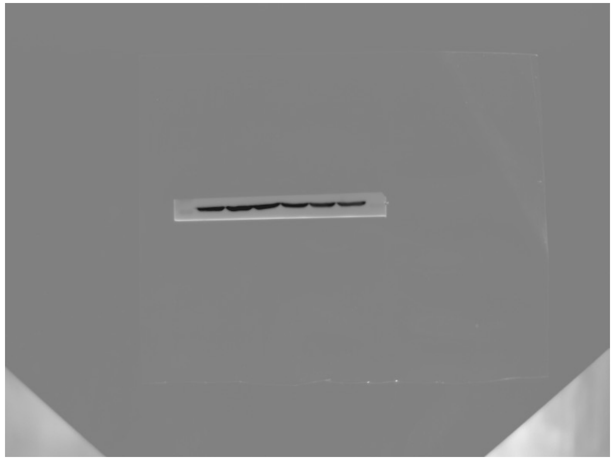

raw data for Figure 3E

AKT
(55kDa, Invitrogen)

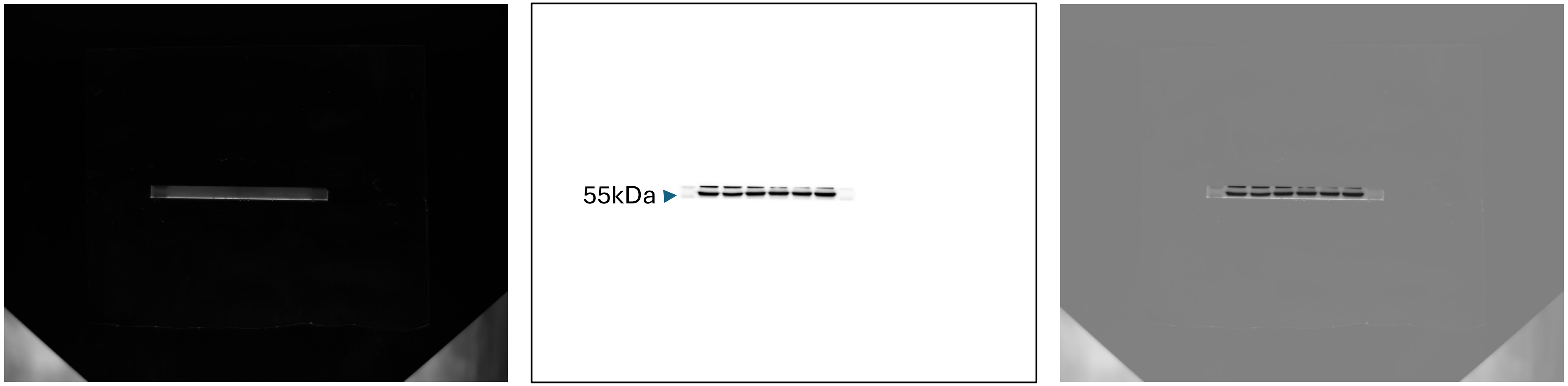

β-Actin
(45kDa , Cell signal)

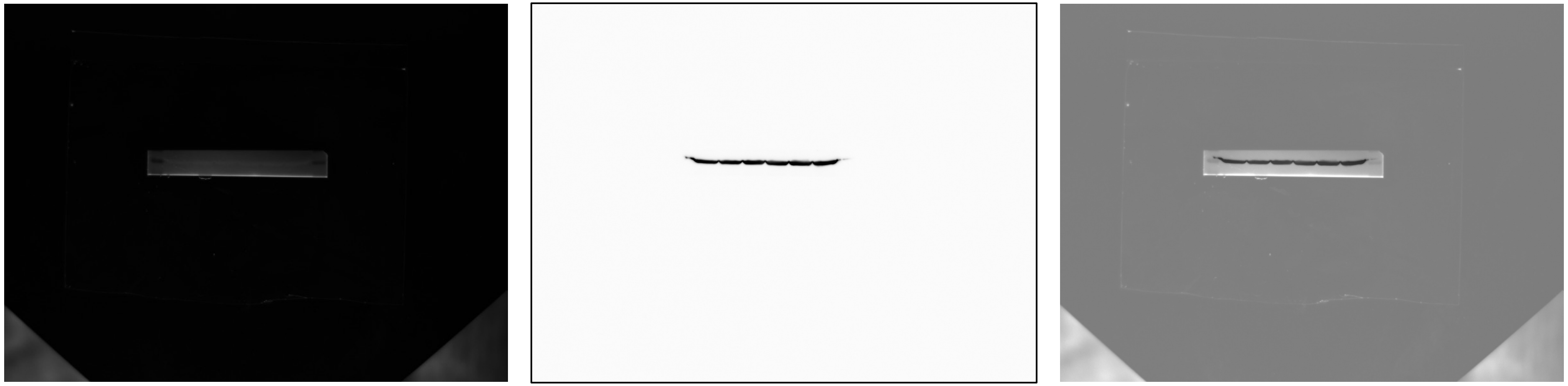

raw data for Figure 3E

Phospho-AKT
(60kDa, Invitrogen)

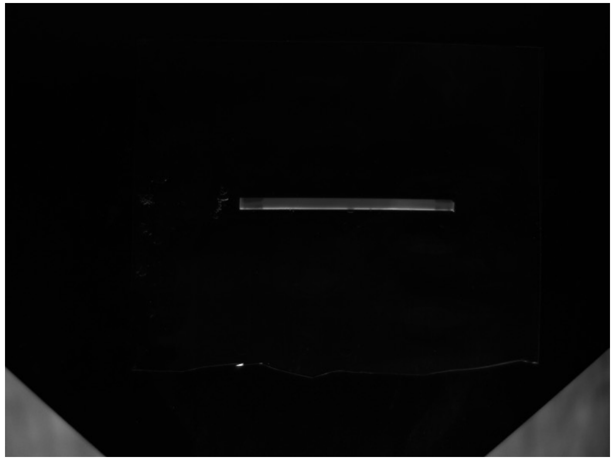

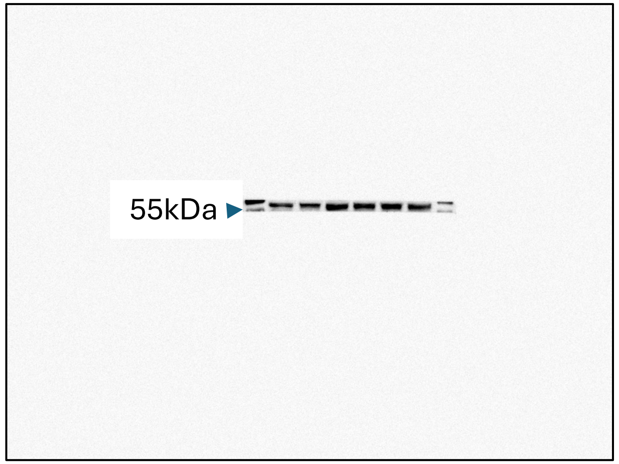

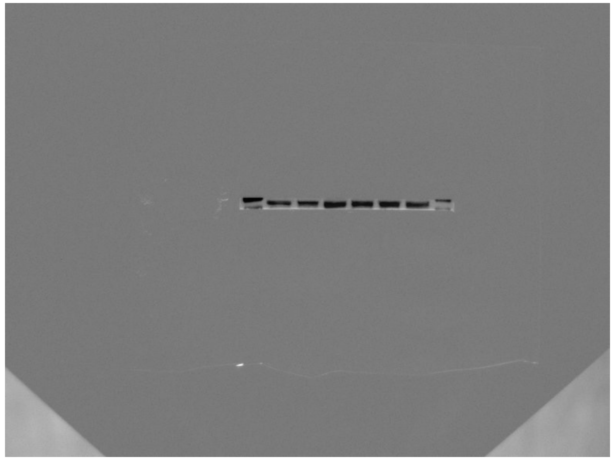

β-Actin
(45kDa , Cell signal)

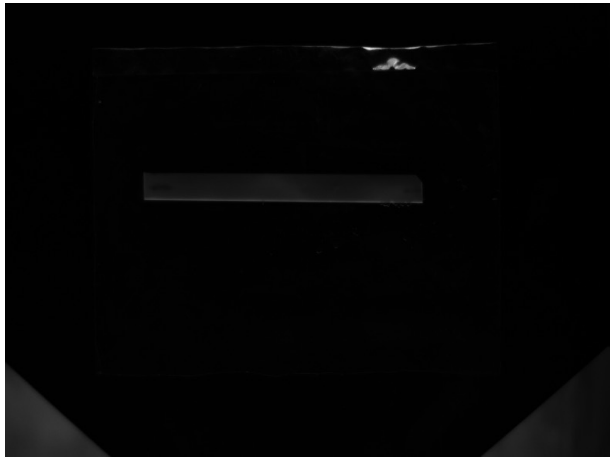

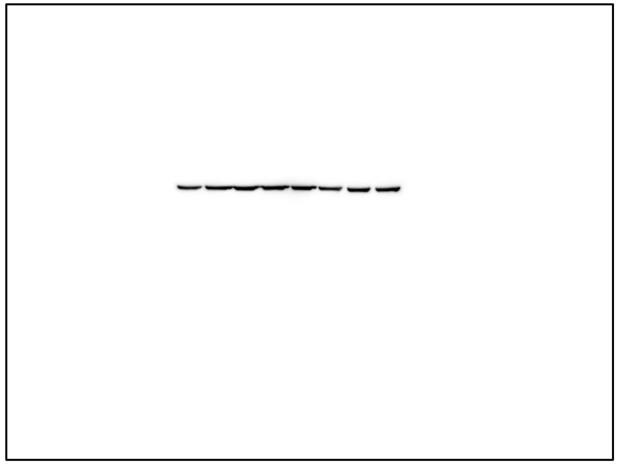

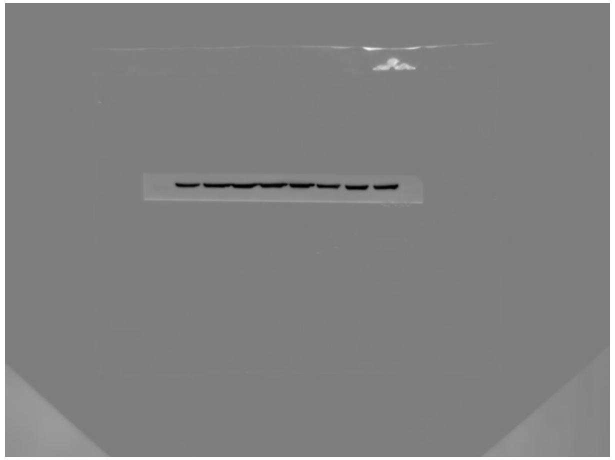

raw data for Figure 3E

AKT
(55kDa, Invitrogen)

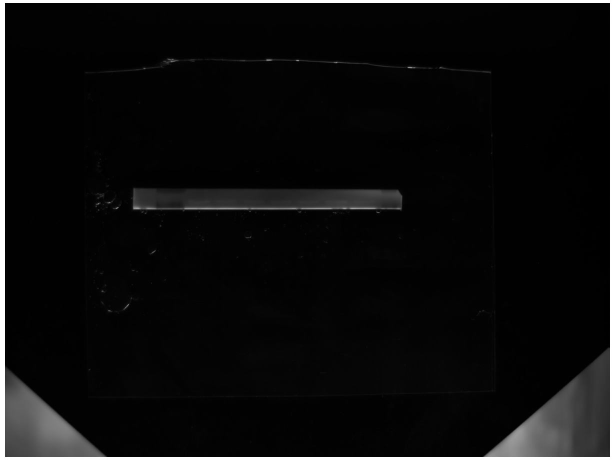

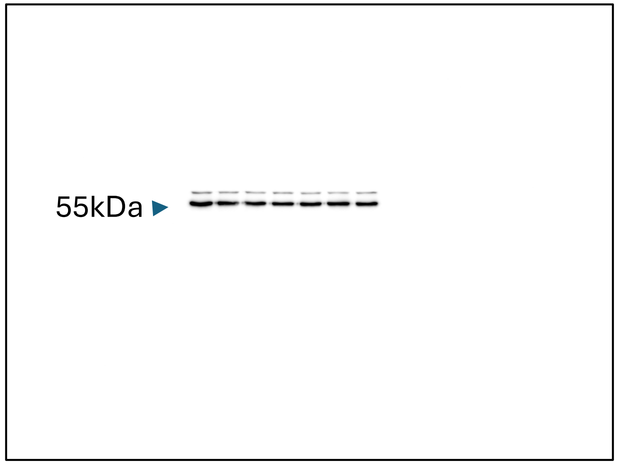

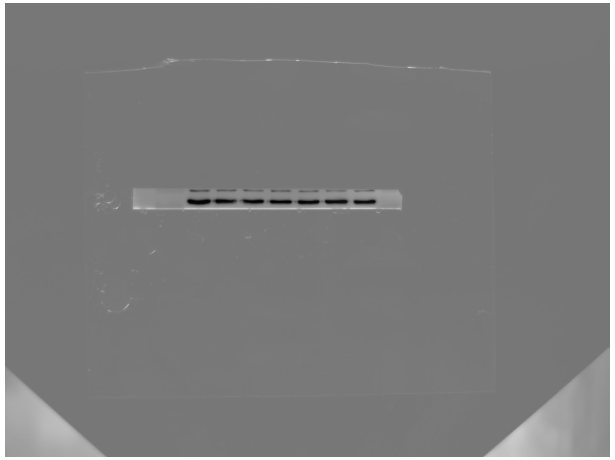

β-Actin
(45kDa , Cell signal)

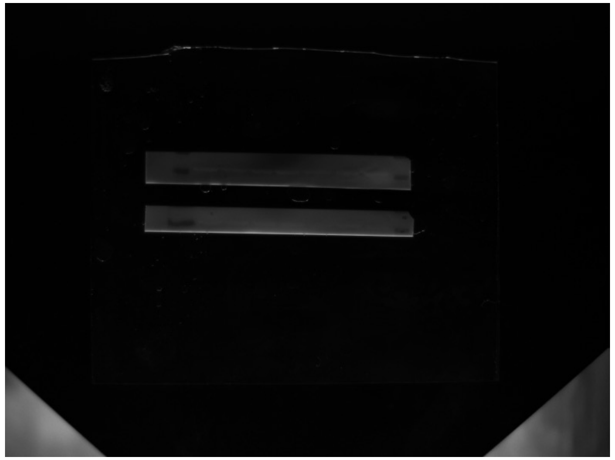

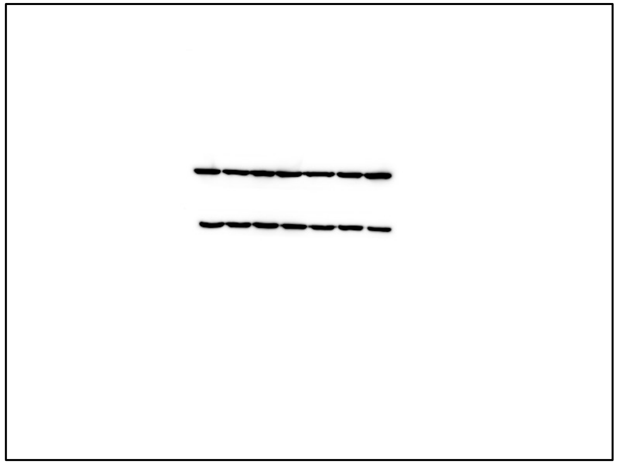

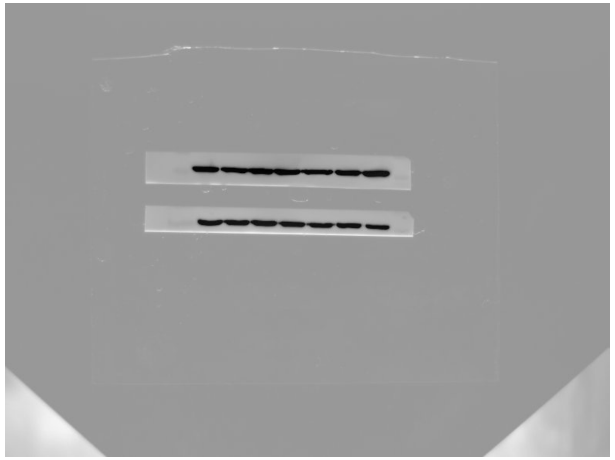

raw data for Figure 3E

Phospho-AKT
(60kDa, Invitrogen)

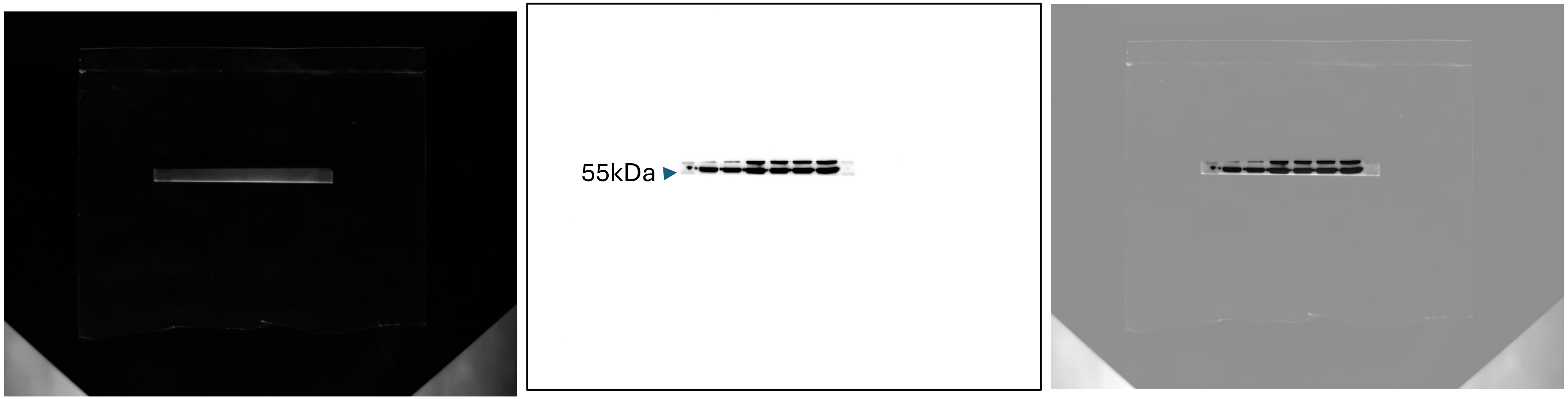

β-Actin
(45kDa , Cell signal)

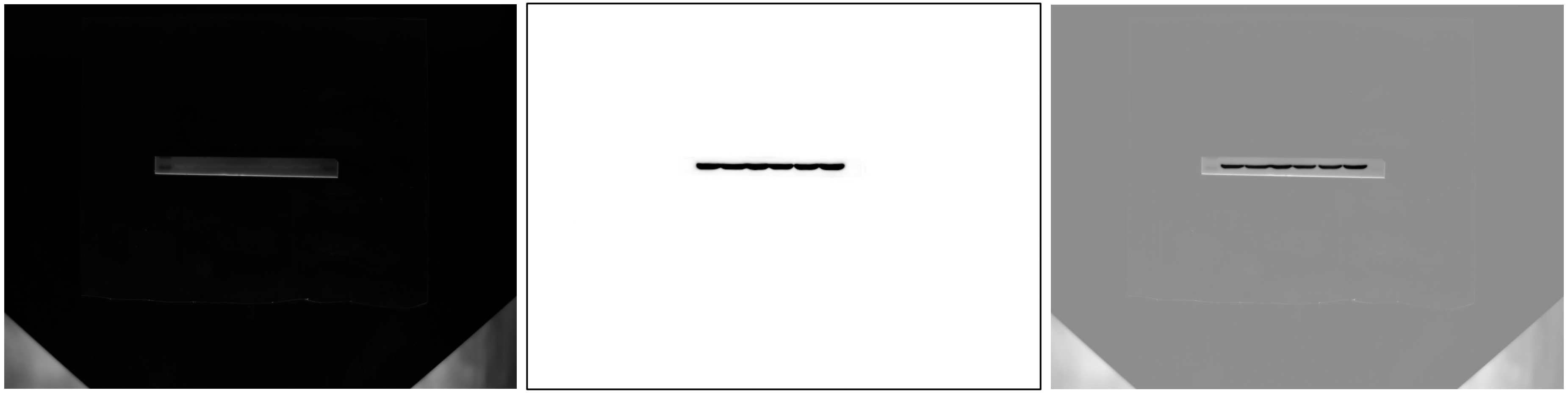

raw data for Figure 3E

AKT
(55kDa, Invitrogen)

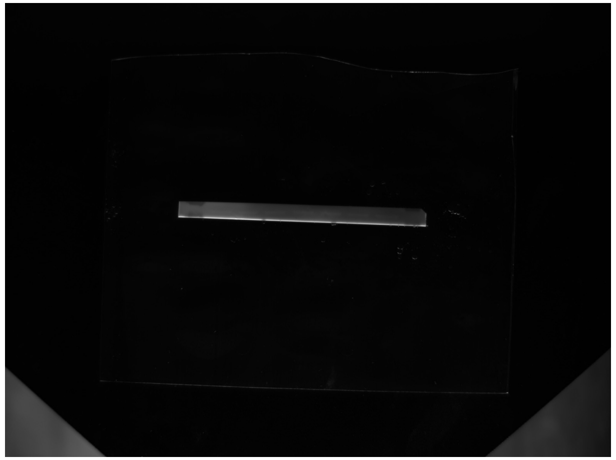

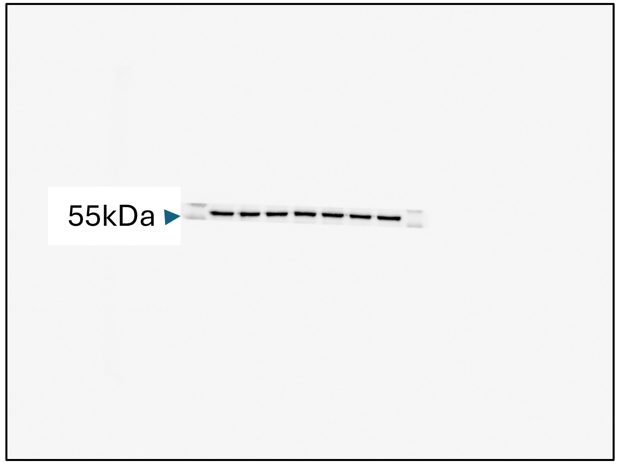

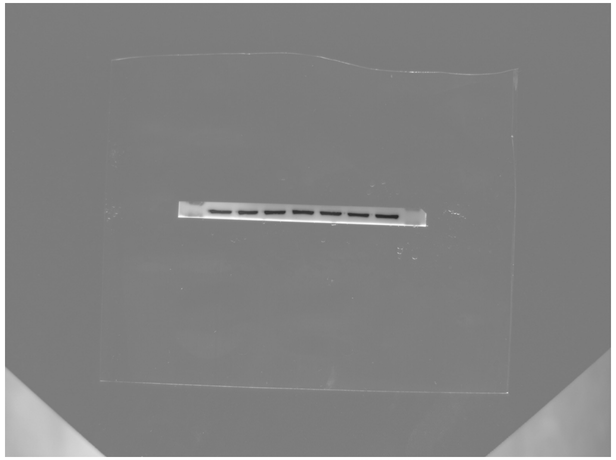

β-Actin
(45kDa , Cell signal)

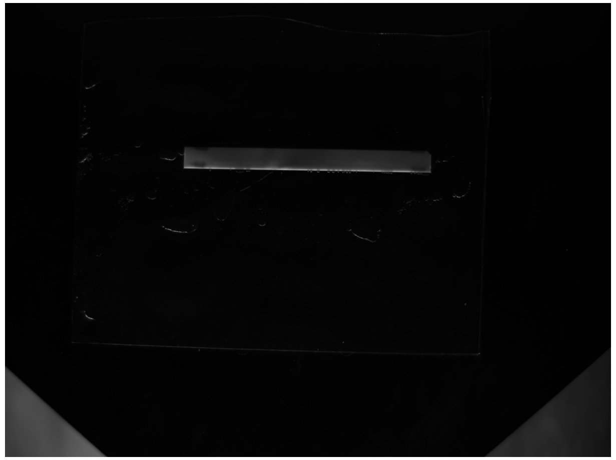

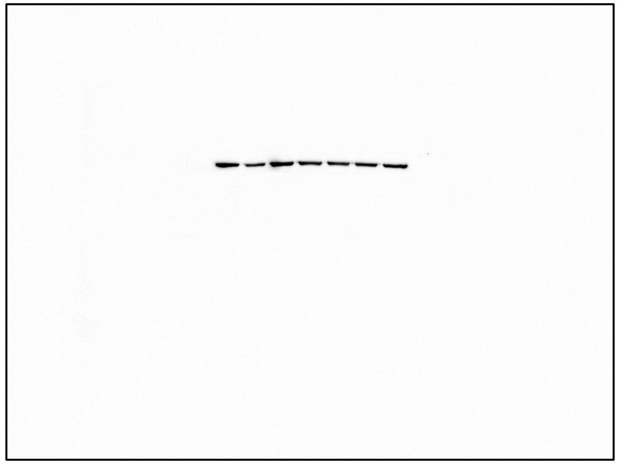

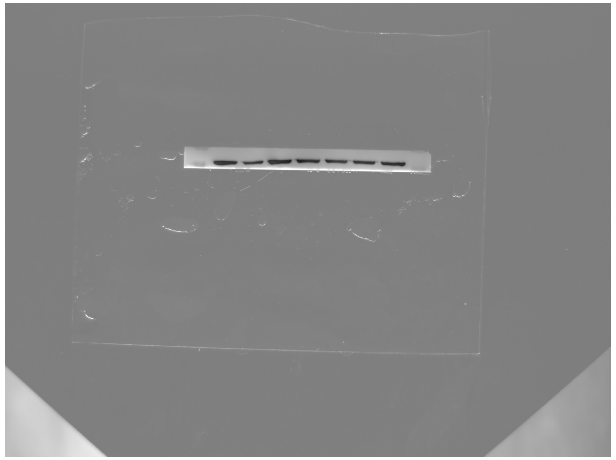

Figure 3F

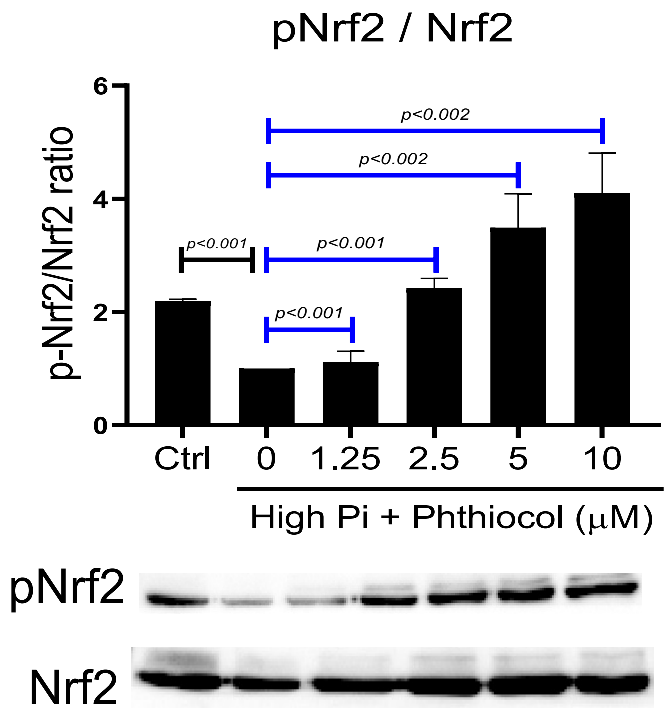
pNrf2 / Nrf2
p-Nrf2/Nrf2 ratio
6
4
2
0
p<0.001
p<0.001
p<0.001
p<0.002
p<0.002
Ctrl
0
1.25
2.5
5
10
High Pi + Phthiocol (μM)
pNrf2
Nrf2

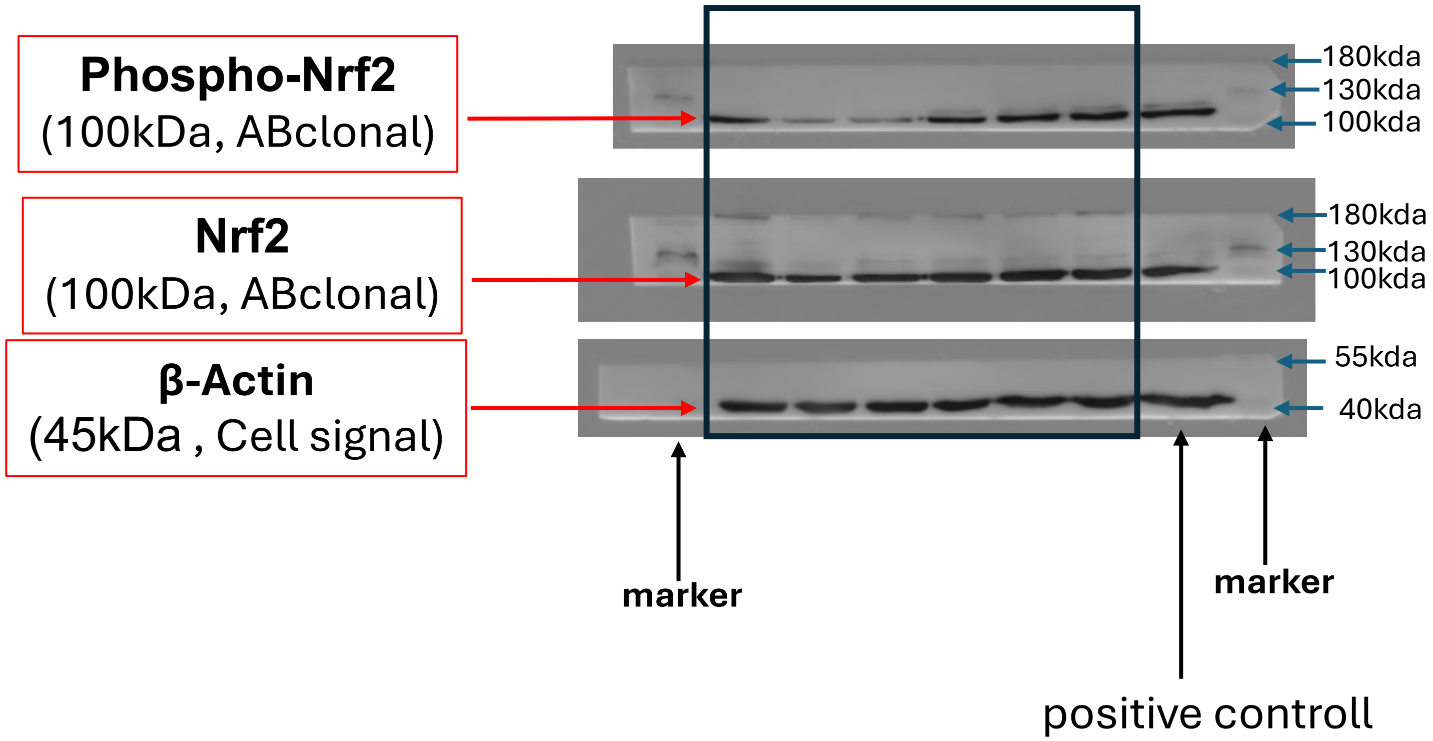
Phospho-Nrf2
(100kDa, ABclonal)
Nrf2
(100kDa, ABclonal)
β-Actin
(45kDa , Cell signal)
180kda
130kda
100kda
180kda
130kda
100kda
55kda
40kda
marker
marker
positive controll

raw data for Figure 3F

Phospho-Nrf2
(100kDa, ABclonal)

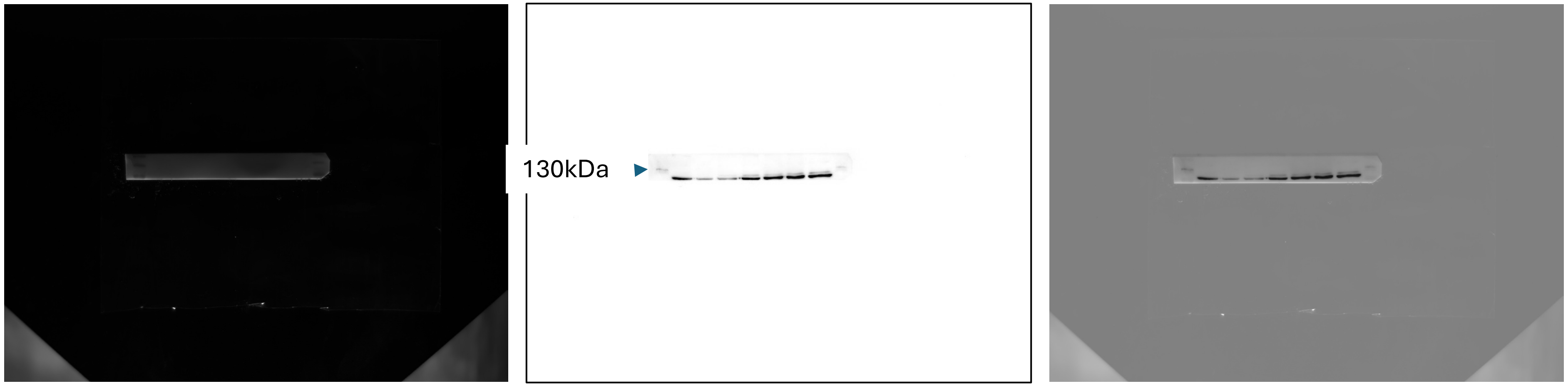

β-Actin
(45kDa , Cell signal)

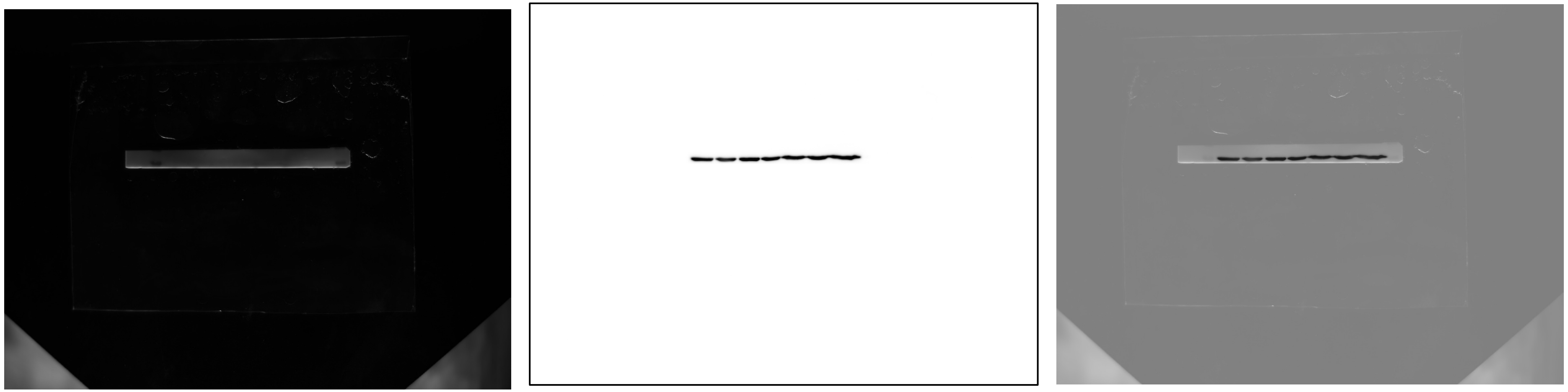

raw data for Figure 3F

Nrf2
(100kDa, ABclonal)

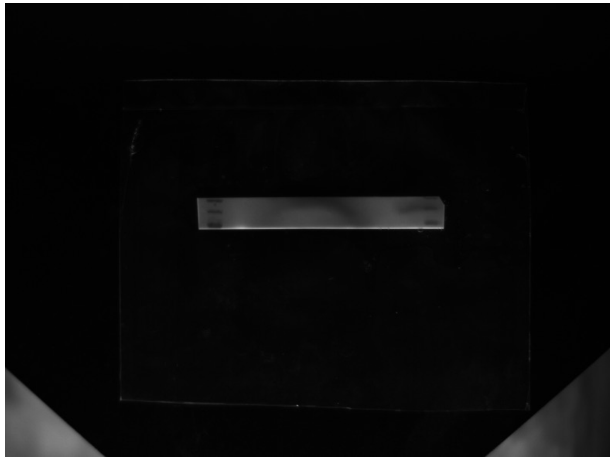

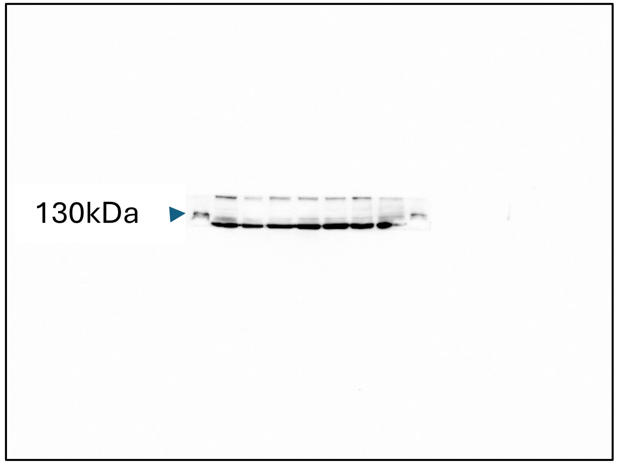

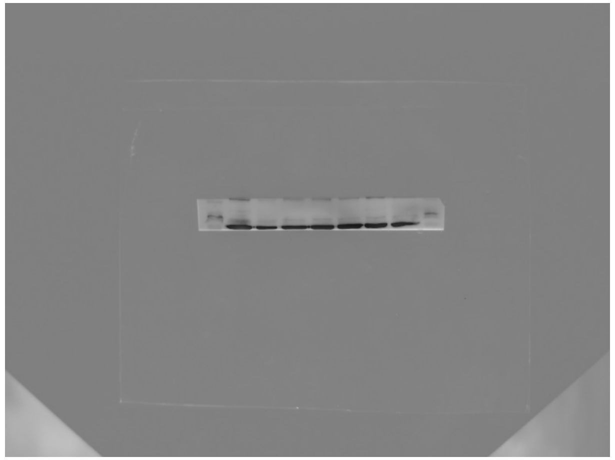

β-Actin
(45kDa , Cell signal)

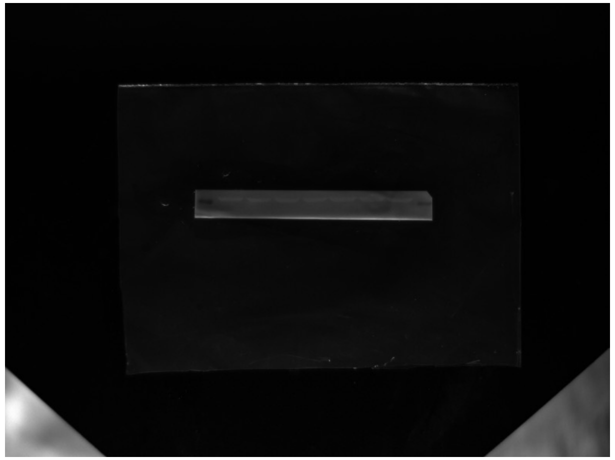

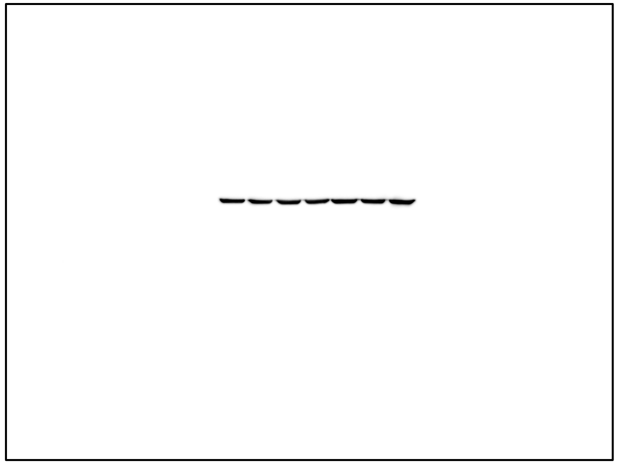

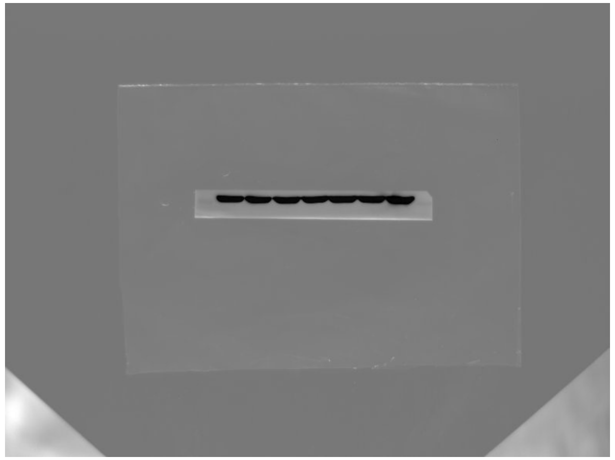

raw data for Figure 3F

Phospho-Nrf2
(100kDa, ABclonal)

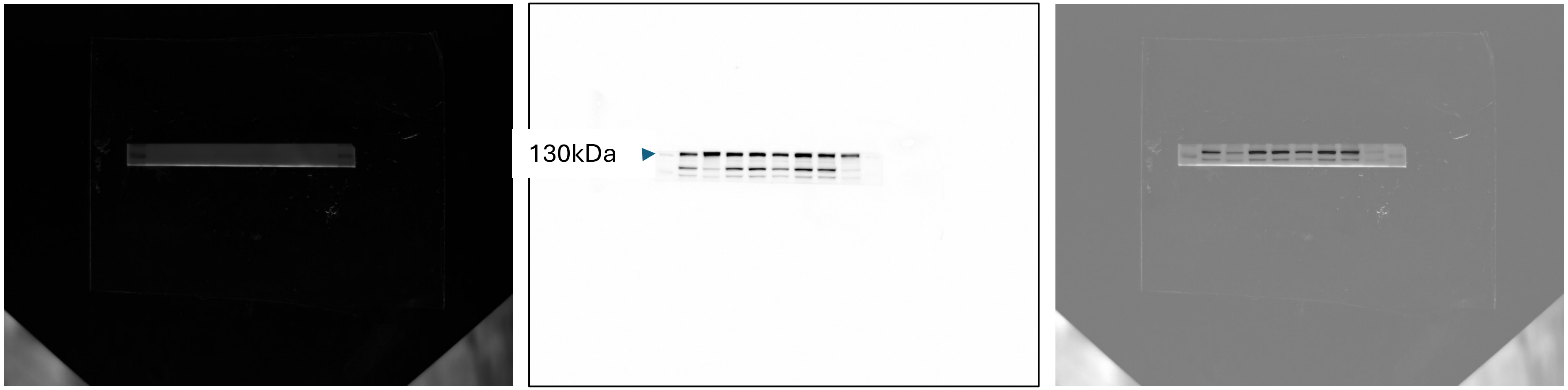

β-Actin
(45kDa , Cell signal)

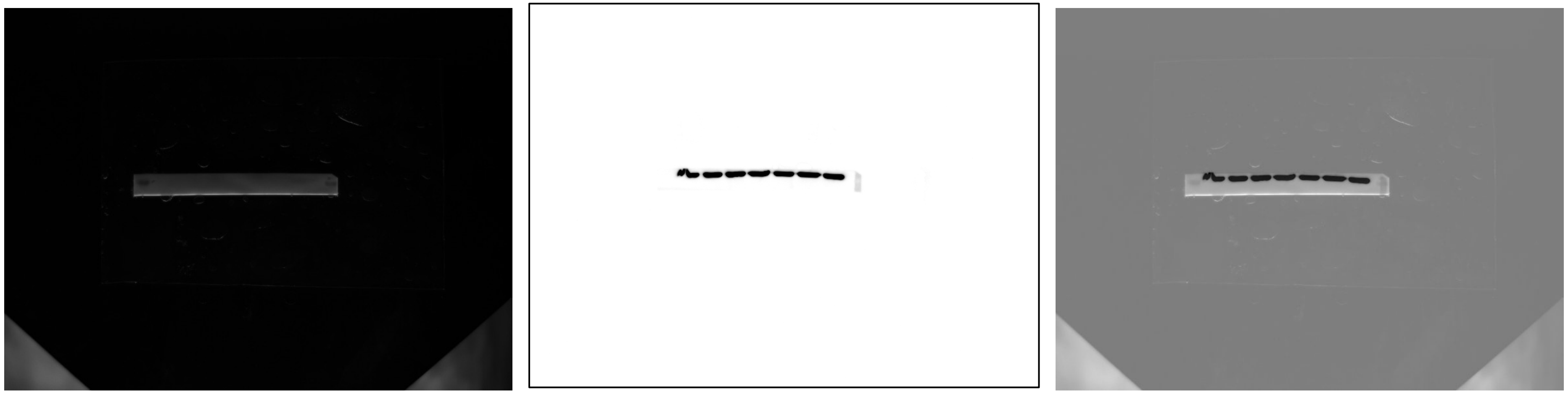

raw data for Figure 3F

Nrf2
(100kDa, ABclonal)

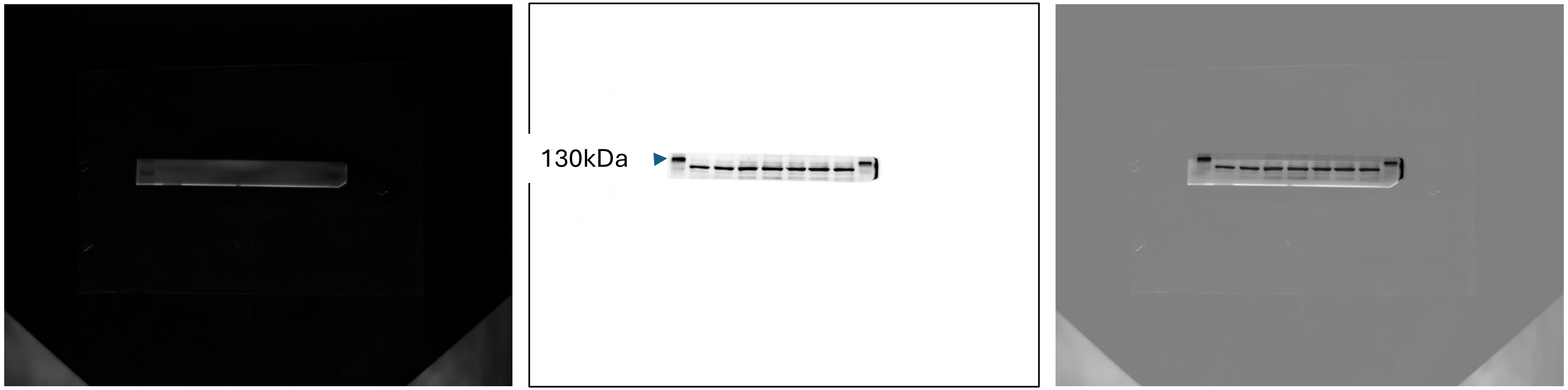

β-Actin
(45kDa , Cell signal)

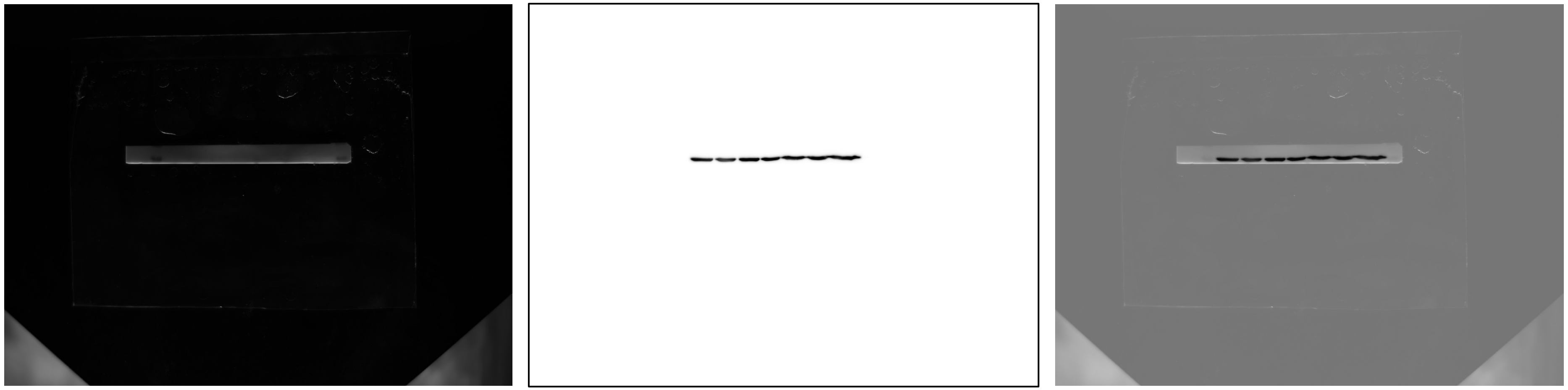

raw data for Figure 3F

Phospho-Nrf2
(100kDa, ABclonal)

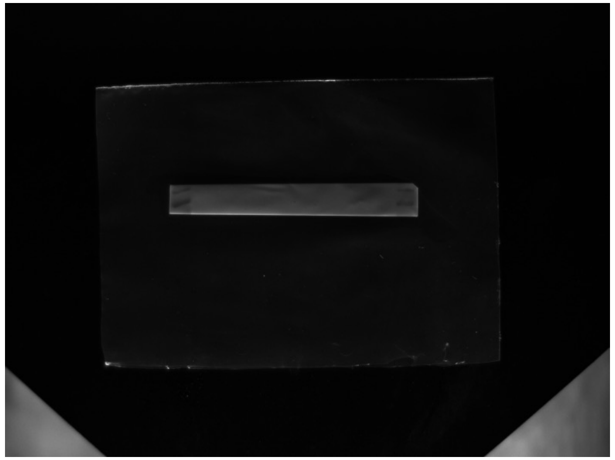

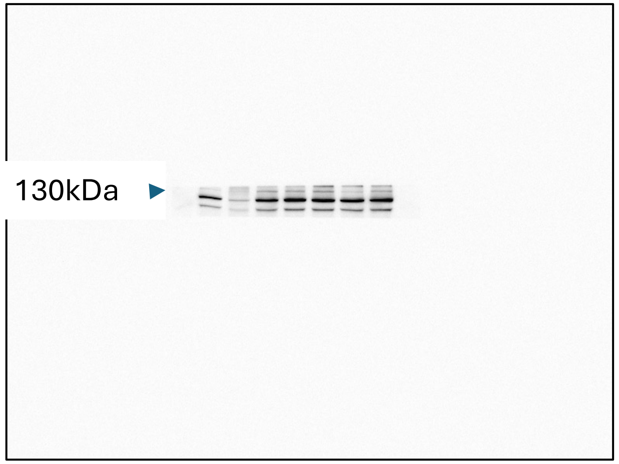

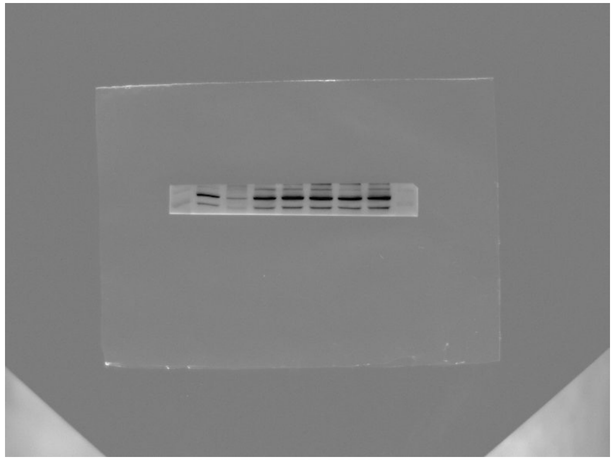

β-Actin
(45kDa , Cell signal)

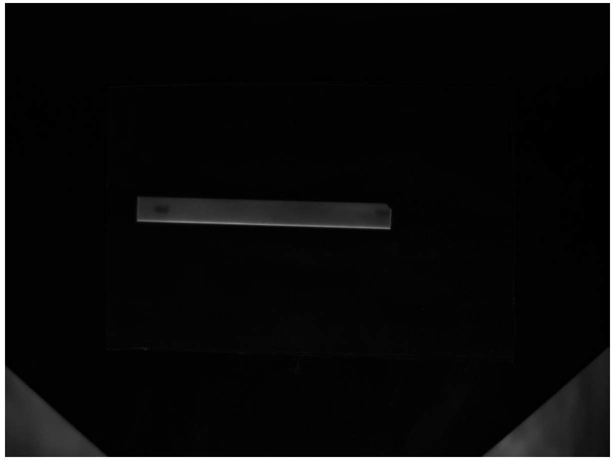

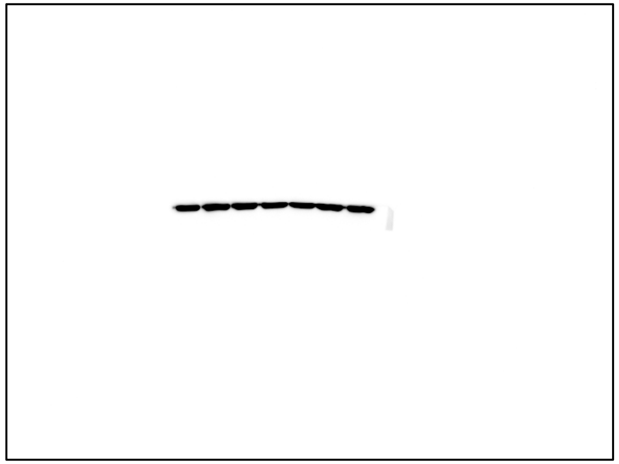

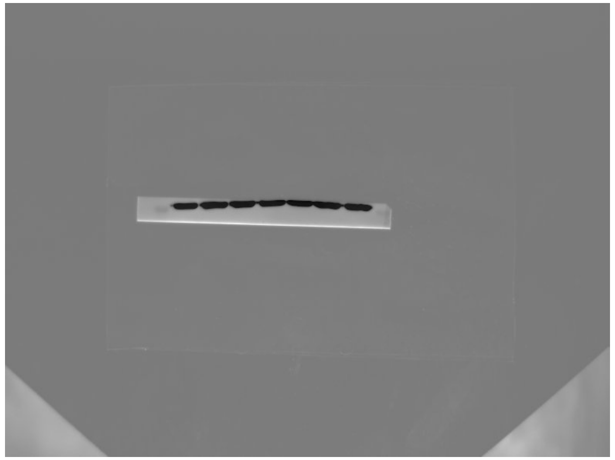

raw data for Figure 3F

Nrf2
(100kDa, ABclonal)

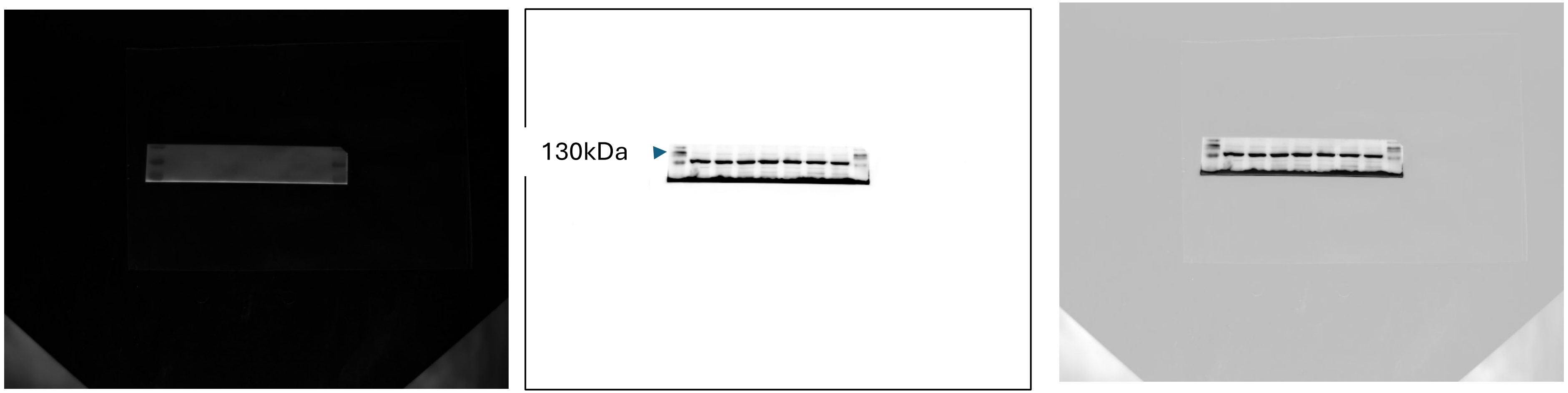

β-Actin
(45kDa , Cell signal)

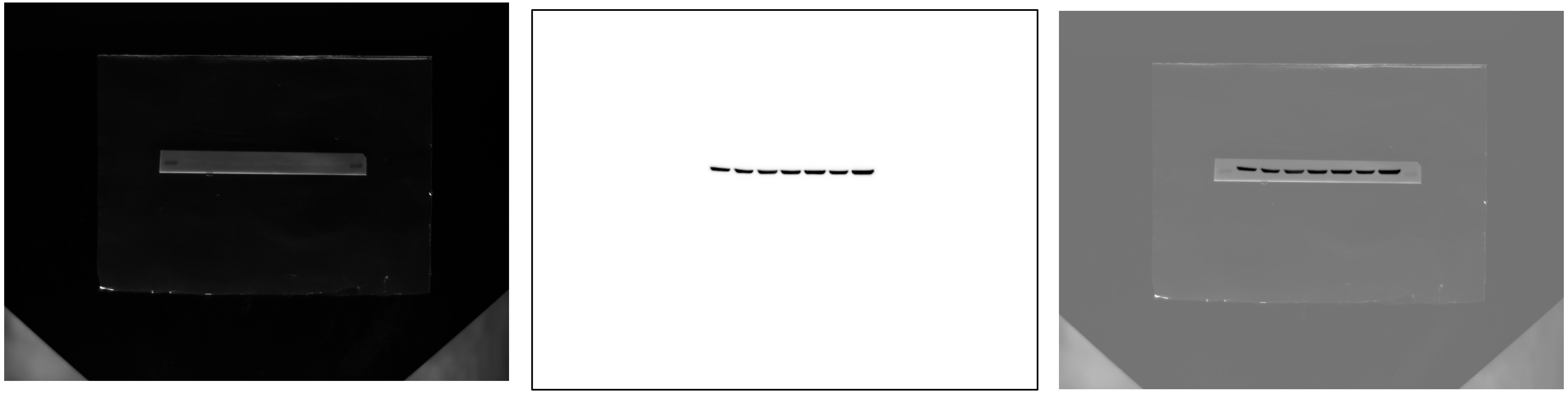

Figure 3G

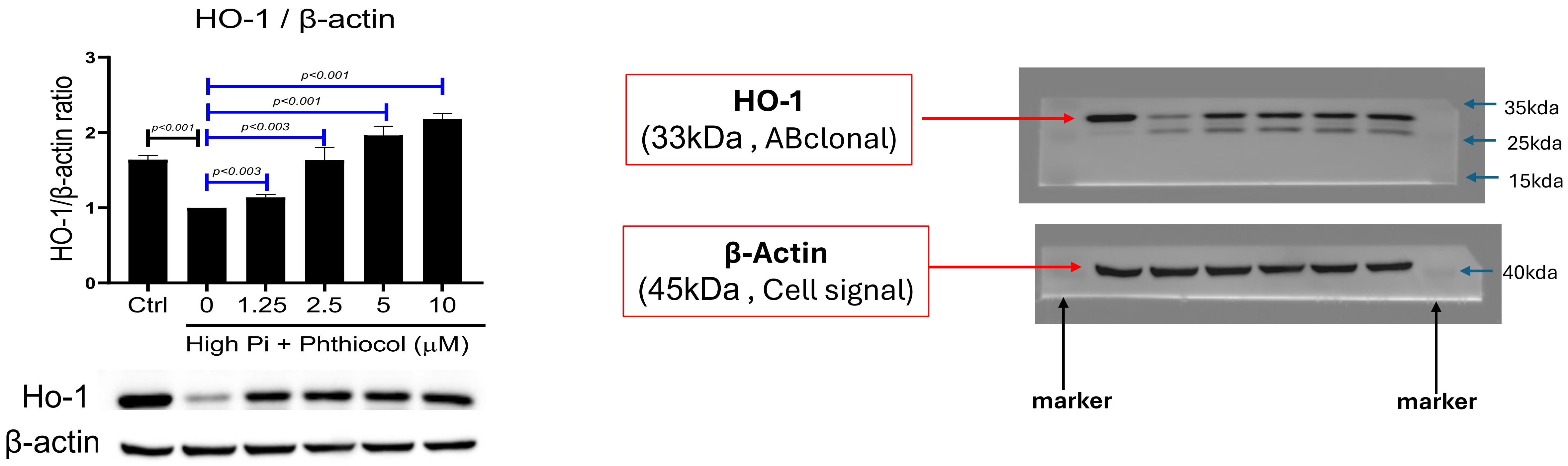
HO-1 / β-actin
HO-1/β-actin ratio
p<0.001
p<0.001
p<0.001
p<0.003
p<0.003
Ctrl
0
1.25
2.5
5
10
High Pi + Phthiocol (μM)
Ho-1
β-actin
HO-1
(33kDa , ABclonal)
β-Actin
(45kDa , Cell signal)
35kda
25kda
15kda
40kda
marker
marker

raw data for Figure 3G

HO-1
(33kDa , ABclonal)

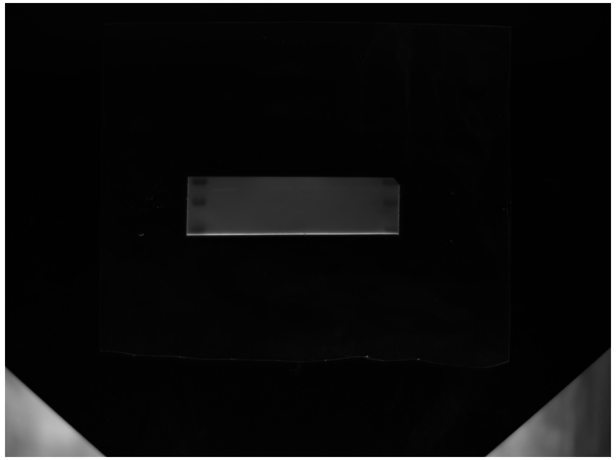

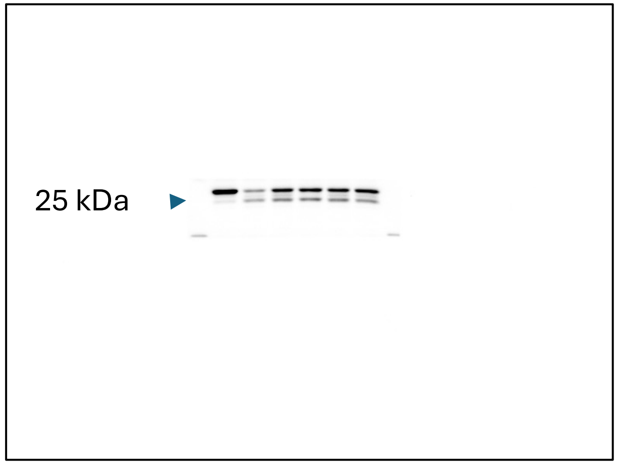

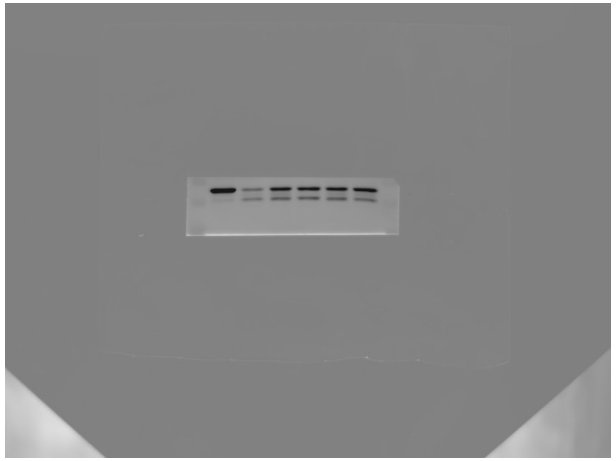

β-Actin
(45kDa , Cell signal)

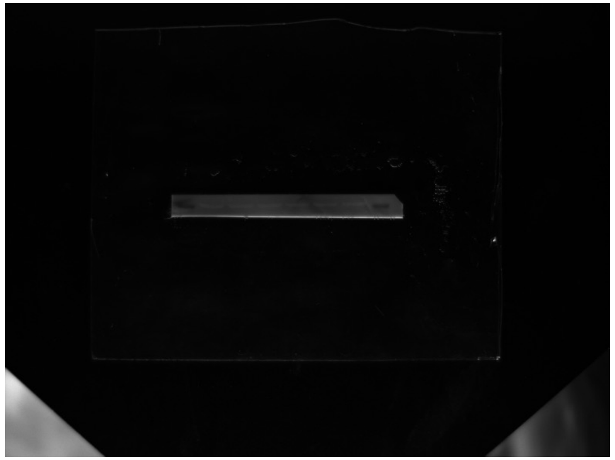

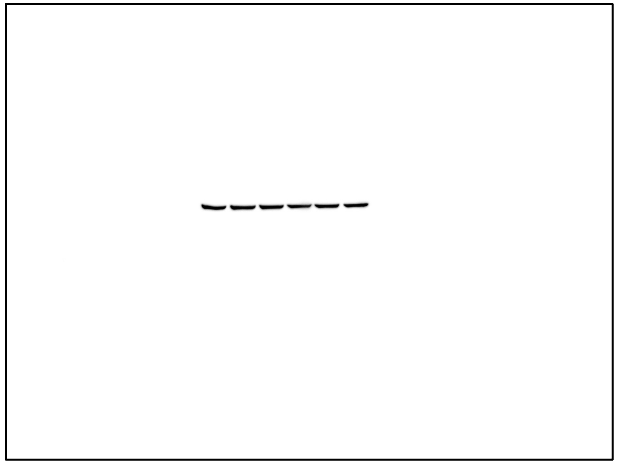

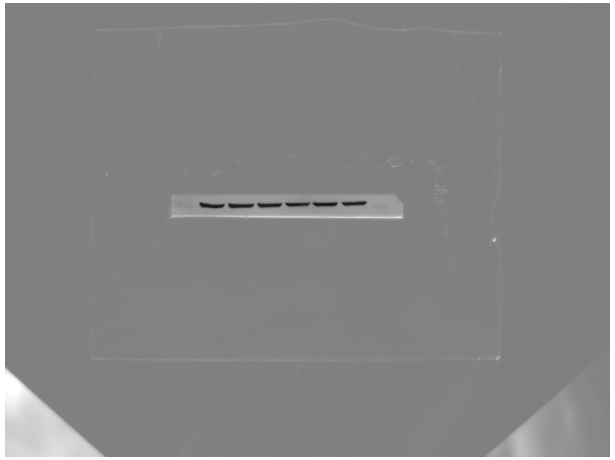

raw data for Figure 3G

HO-1
(33kDa , ABclonal)

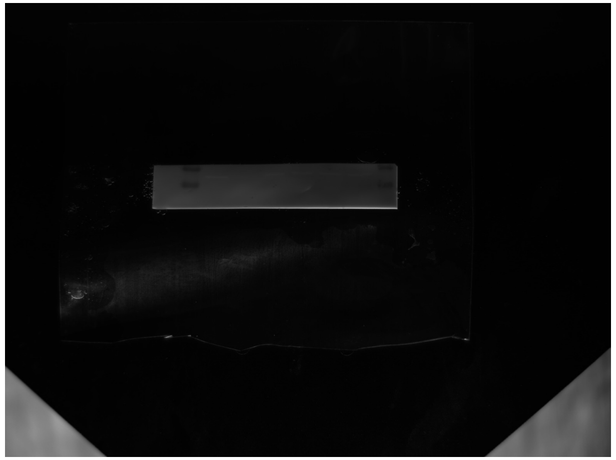

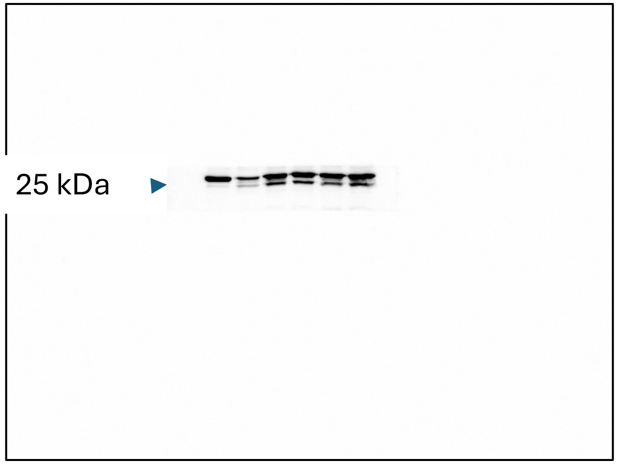

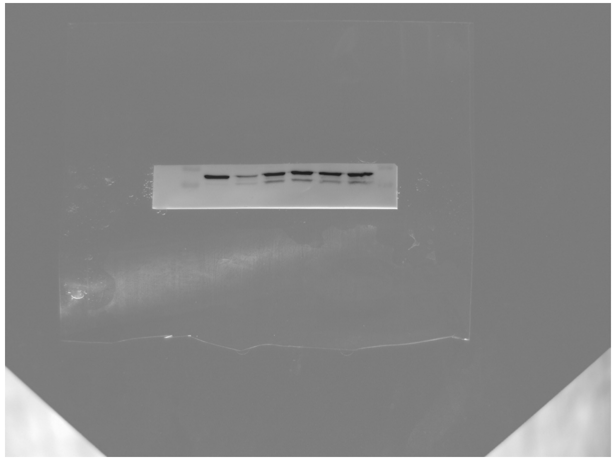

β-Actin
(45kDa , Cell signal)

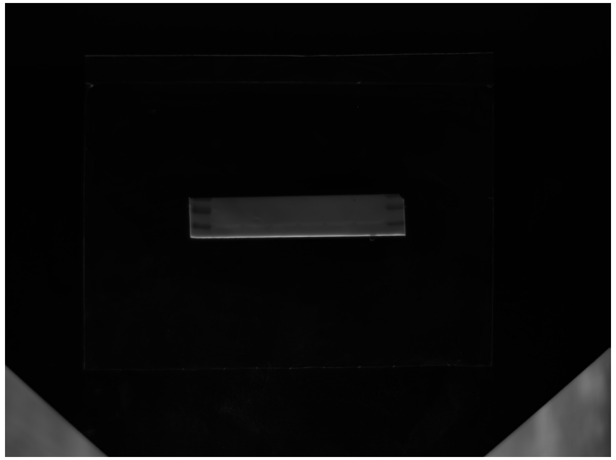

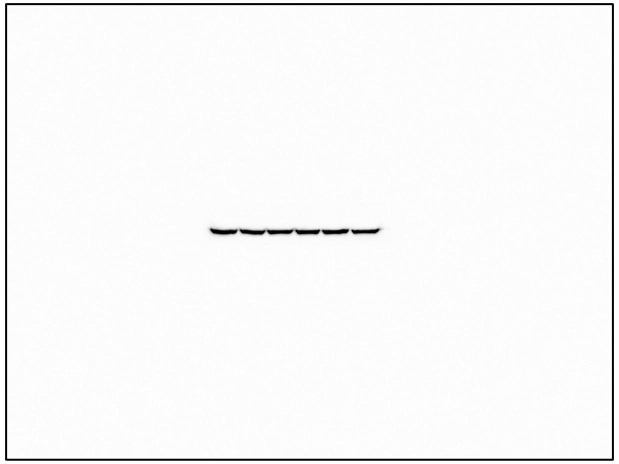

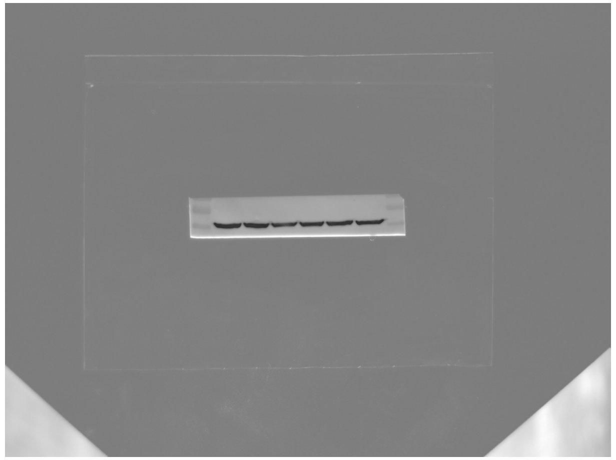

raw data for Figure 3G

HO-1
(33kDa , ABclonal)

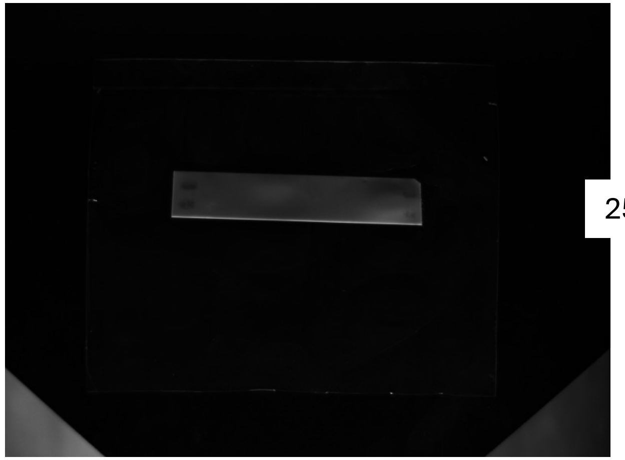

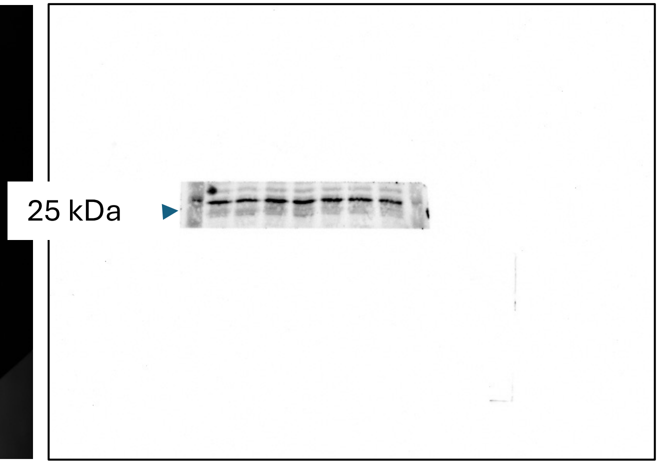

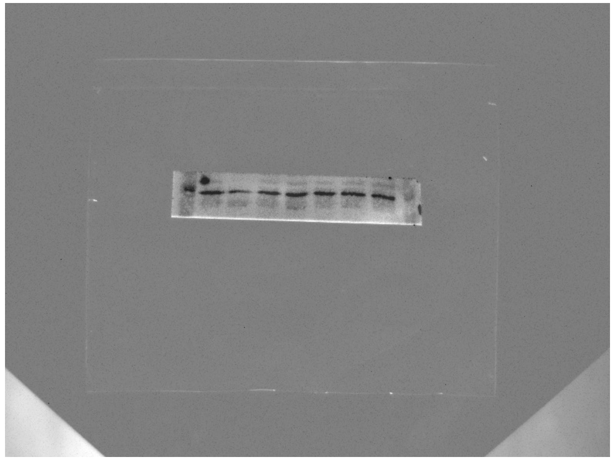

β-Actin
(45kDa , Cell signal)

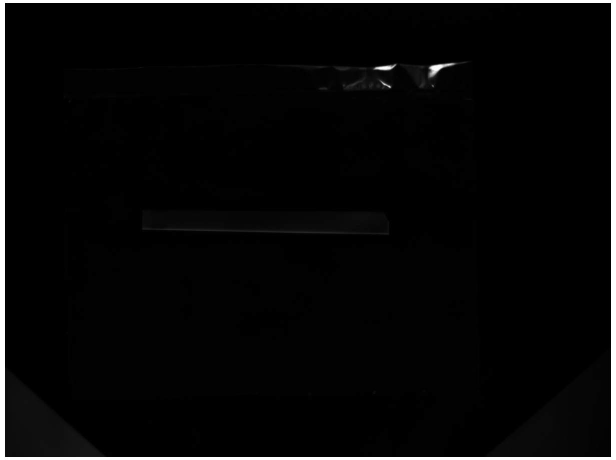

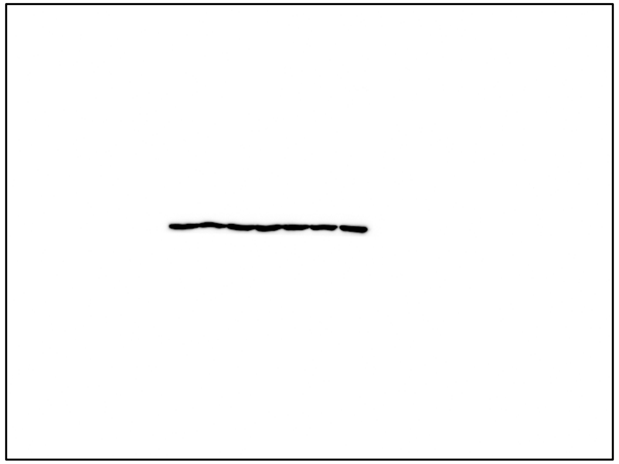

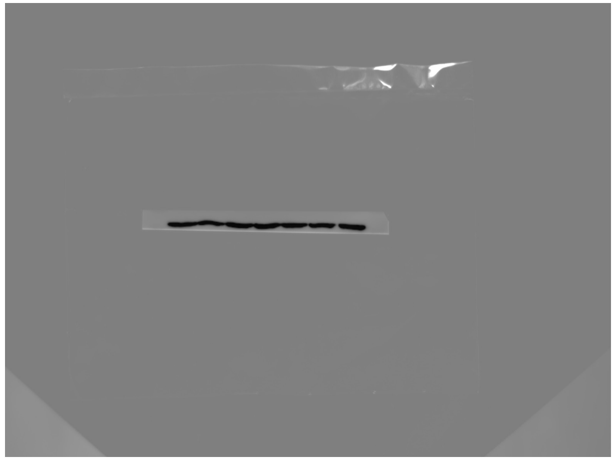

Figure 4A

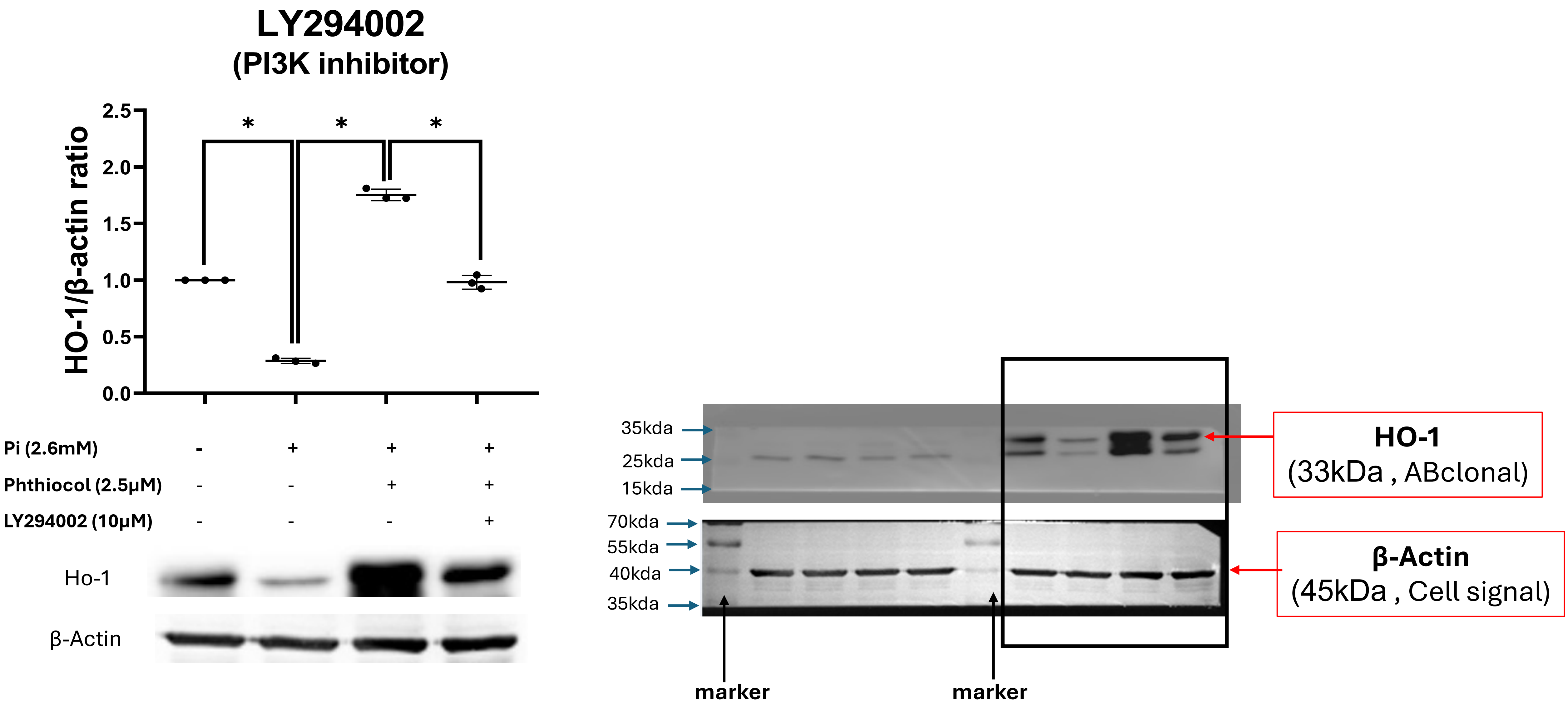
LY294002
(PI3K inhibitor)
HO-1/β-actin ratio
2.5
2.0
1.5
1.0
0.5
0.0
*
*
*
Pi (2.6mM) - + + +
Phthiocol (2.5μM) - - + +
LY294002 (10μM) - - - +
Ho-1
β-Actin
35kda
25kda
15kda
70kda
55kda
40kda
35kda
HO-1
(33kDa , ABclonal)
β-Actin
(45kDa , Cell signal)
marker
marker

raw data for Figure 4A

HO-1
(33kDa , ABclonal)

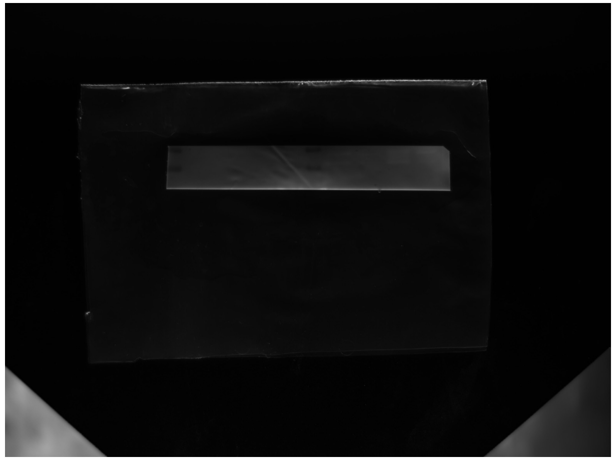

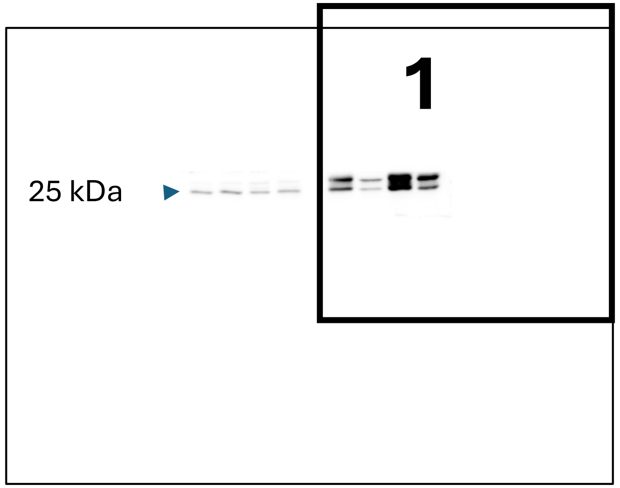

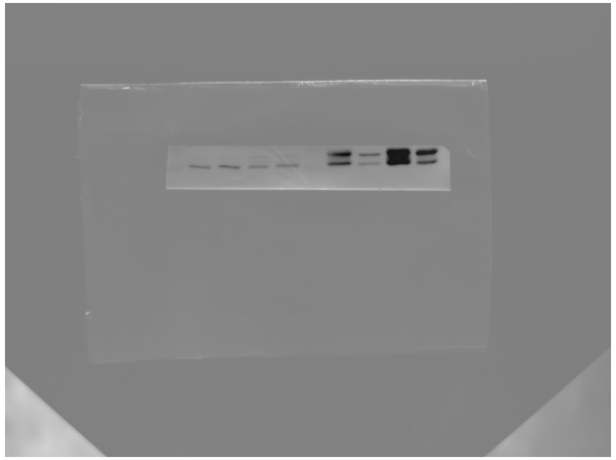

β-Actin
(45kDa , Cell signal)

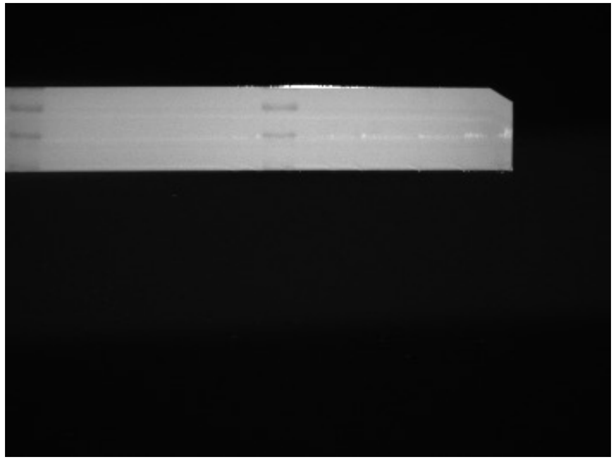

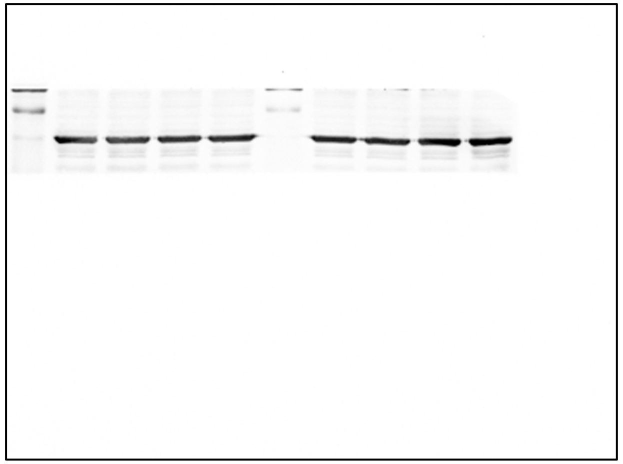

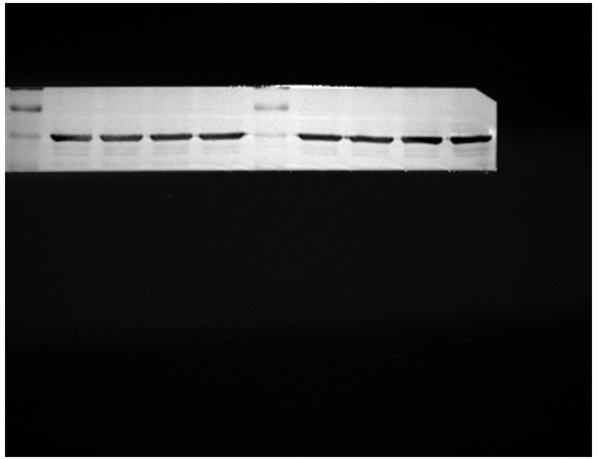

raw data for Figure 4A

HO-1
(33kDa , ABclonal)

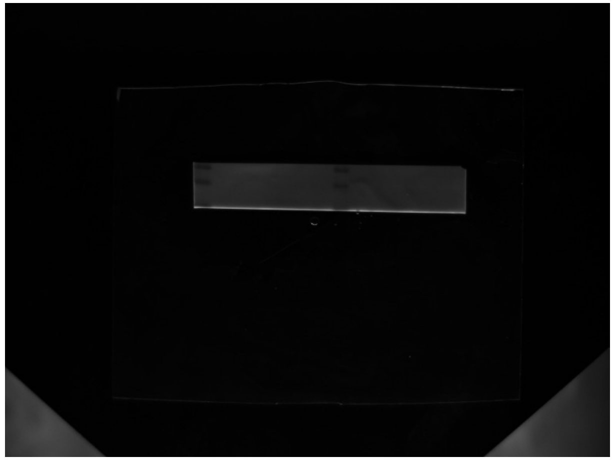

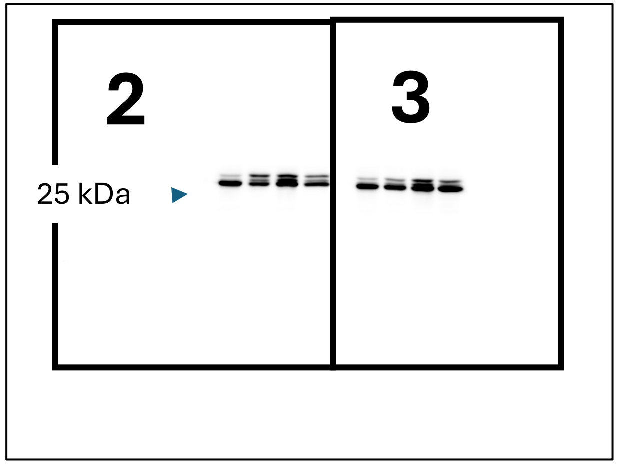

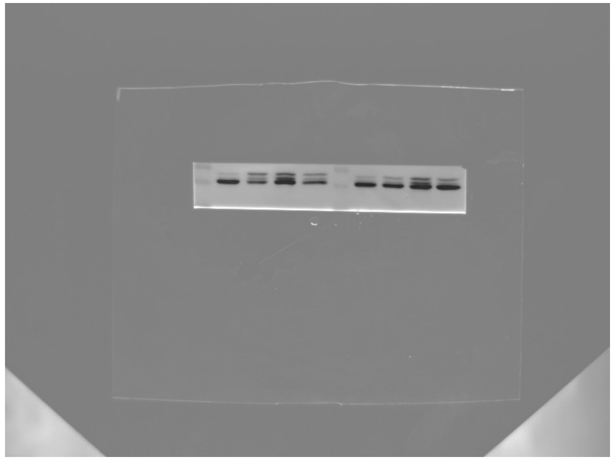

β-Actin
(45kDa , Cell signal)

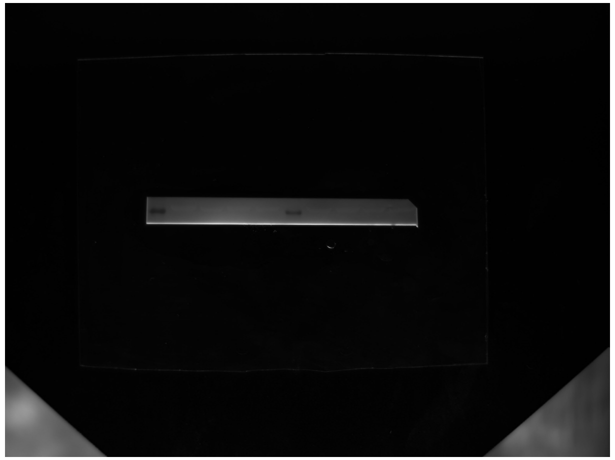

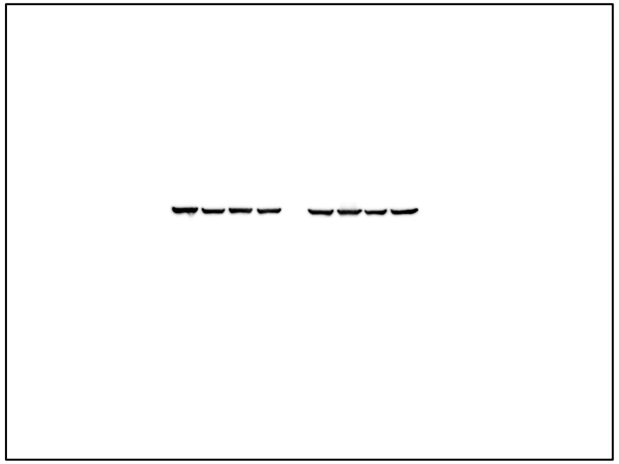

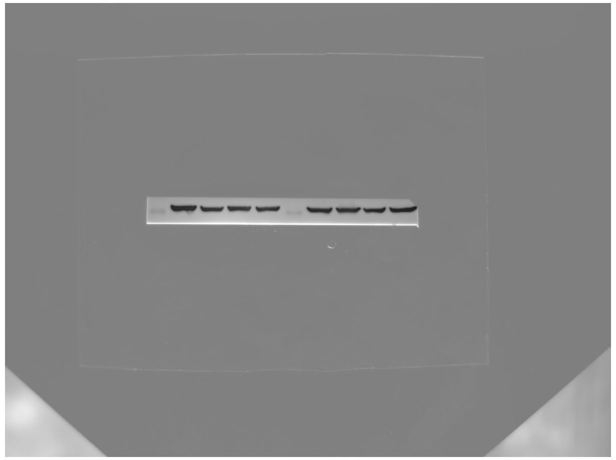

Figure 4B

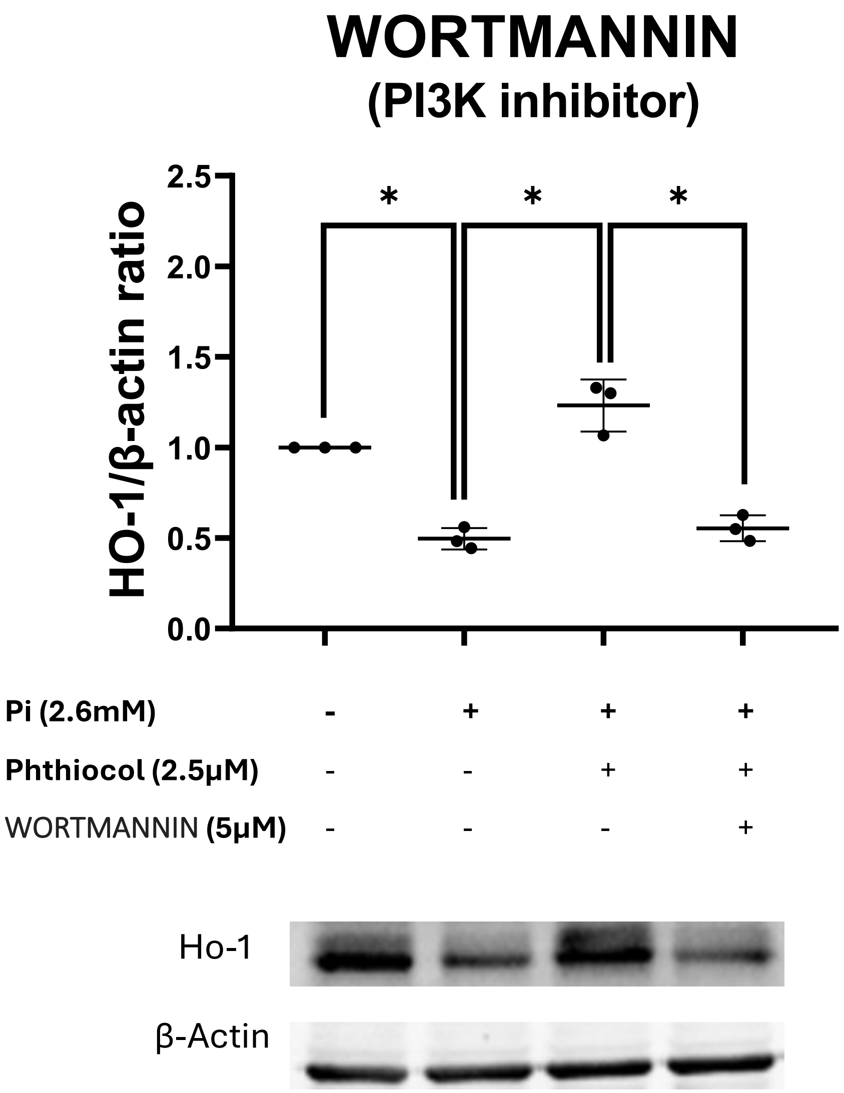
WORTMANNIN
(PI3K inhibitor)
HO-1/β-actin ratio
2.5
2.0
1.5
1.0
0.5
0.0
*
*
*
Pi (2.6mM) - + + +
Phthiocol (2.5μM) - - + +
WORTMANNIN (5μM) - - - +
Ho-1
β-Actin

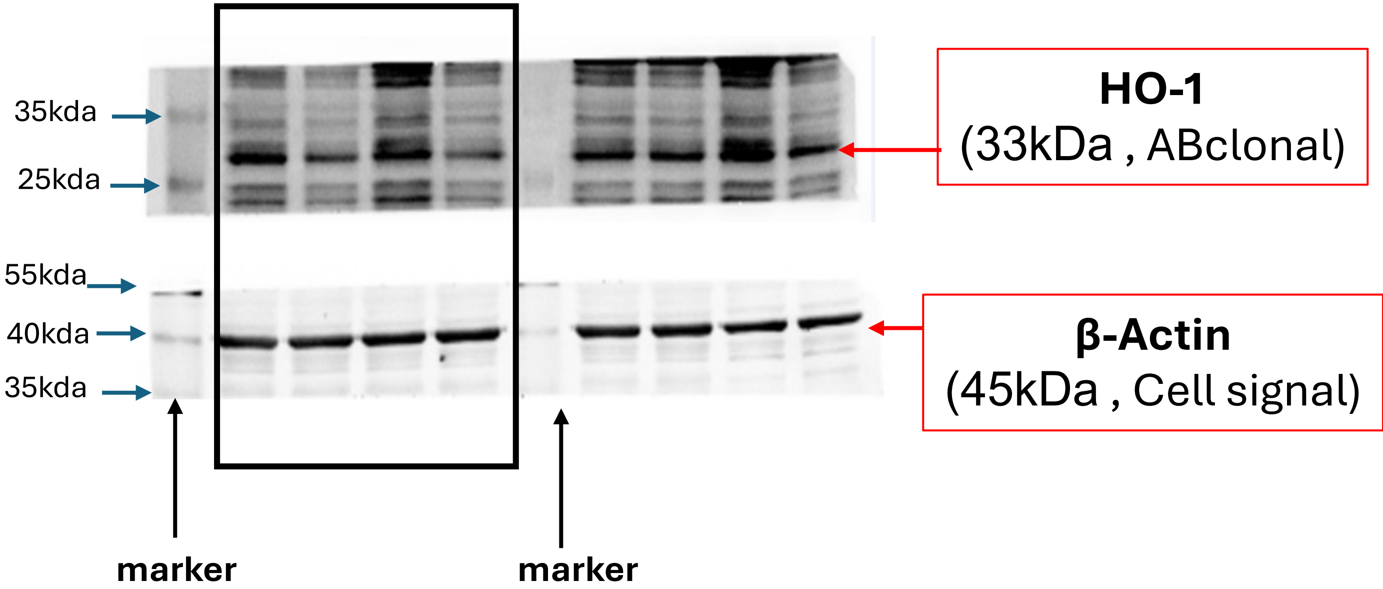
35kda
25kda
55kda
40kda
35kda
marker
marker
HO-1
(33kDa , ABclonal)
β-Actin
(45kDa , Cell signal)

raw data for Figure 4B

HO-1
(33kDa , ABclonal)

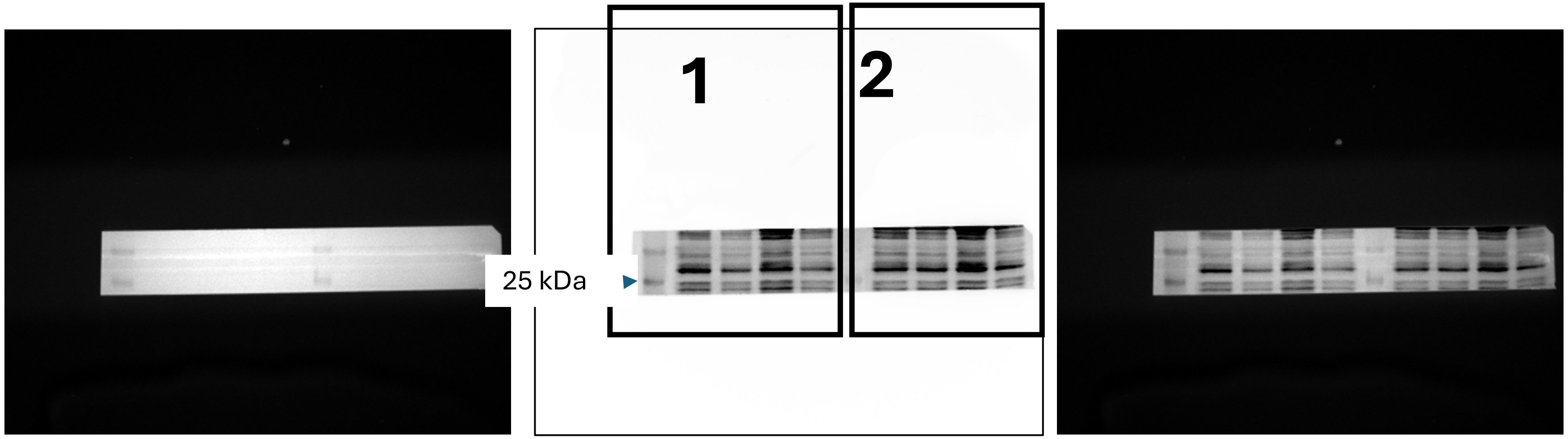

β-Actin
(45kDa , Cell signal)

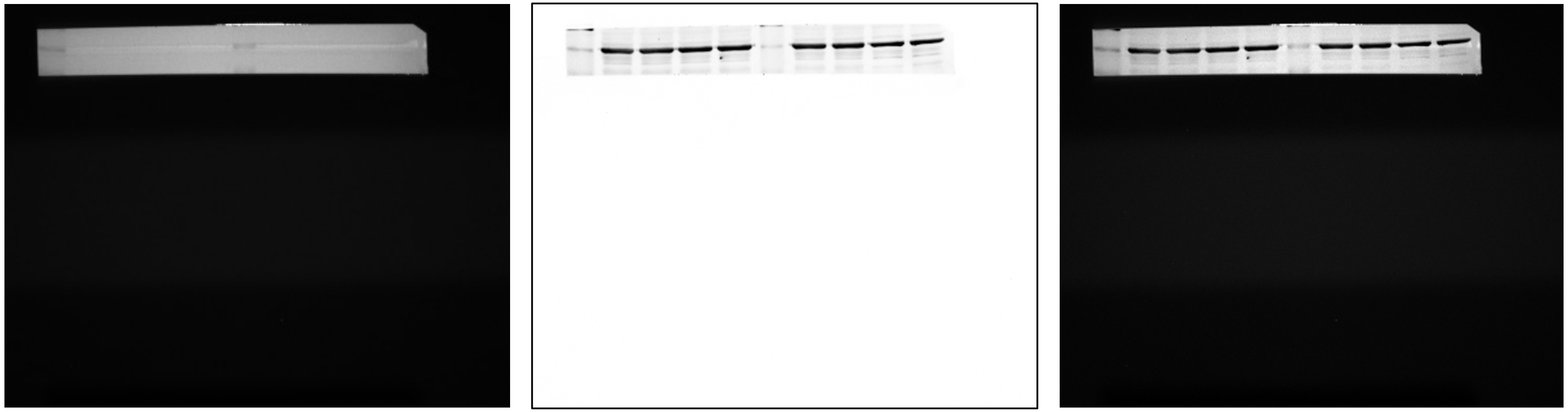

raw data for Figure 4B

HO-1
(33kDa , ABclonal)

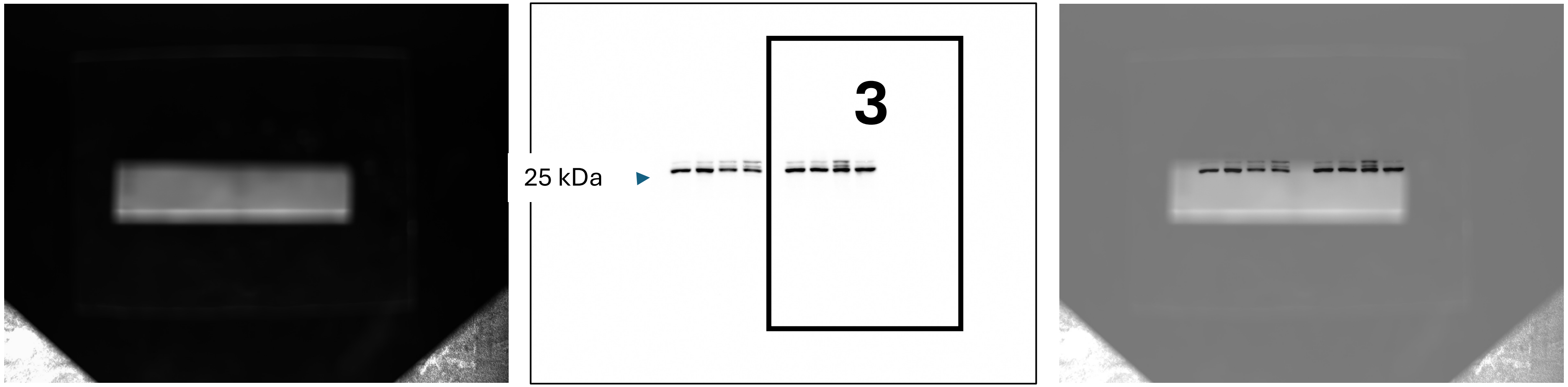

β-Actin
(45kDa , Cell signal)

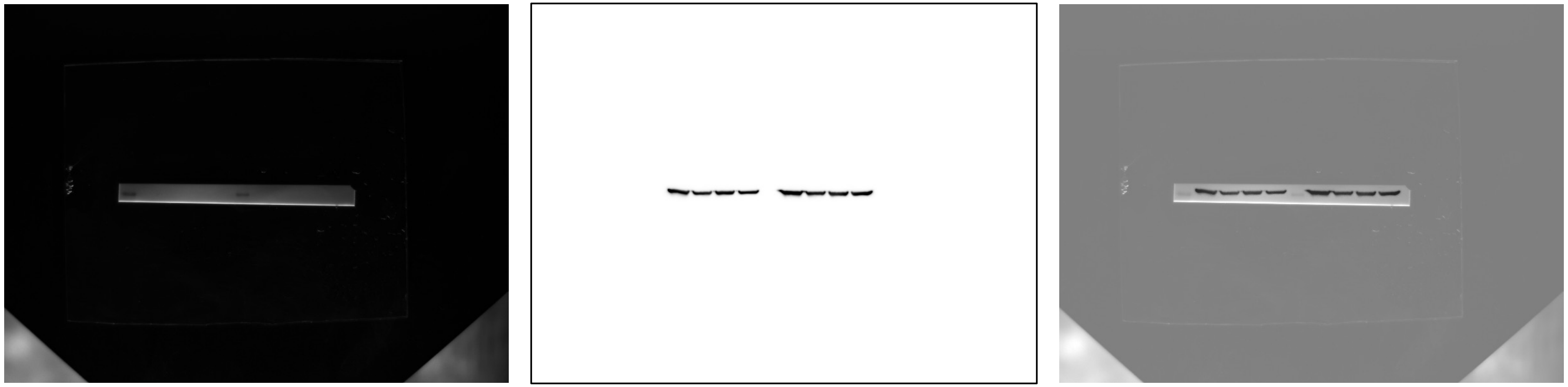

Figure 4C

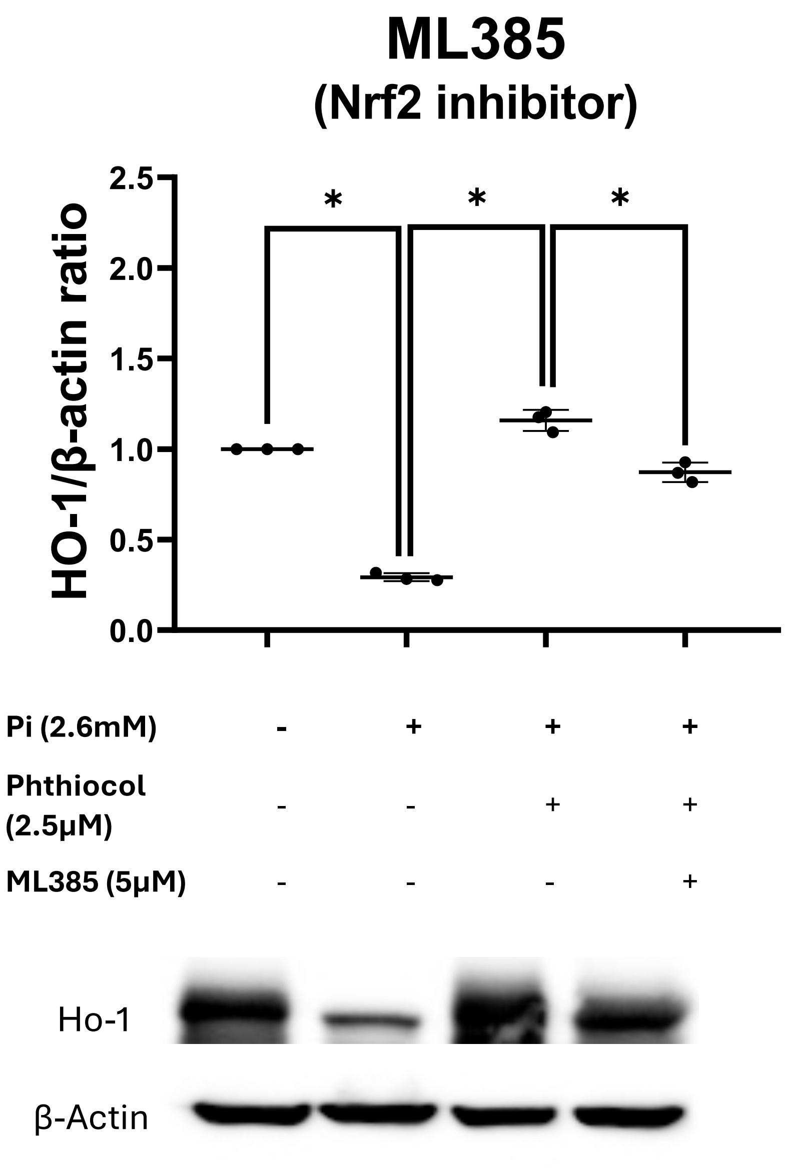

ML385
(Nrf2 inhibitor)
HO-1/β-actin ratio
2.5
2.0
1.5
1.0
0.5
0.0
*
*
*
Pi (2.6mM) - + + +
Phthiocol (2.5μM) - - + +
ML385 (5μM) - - - +
Ho-1
β-Actin

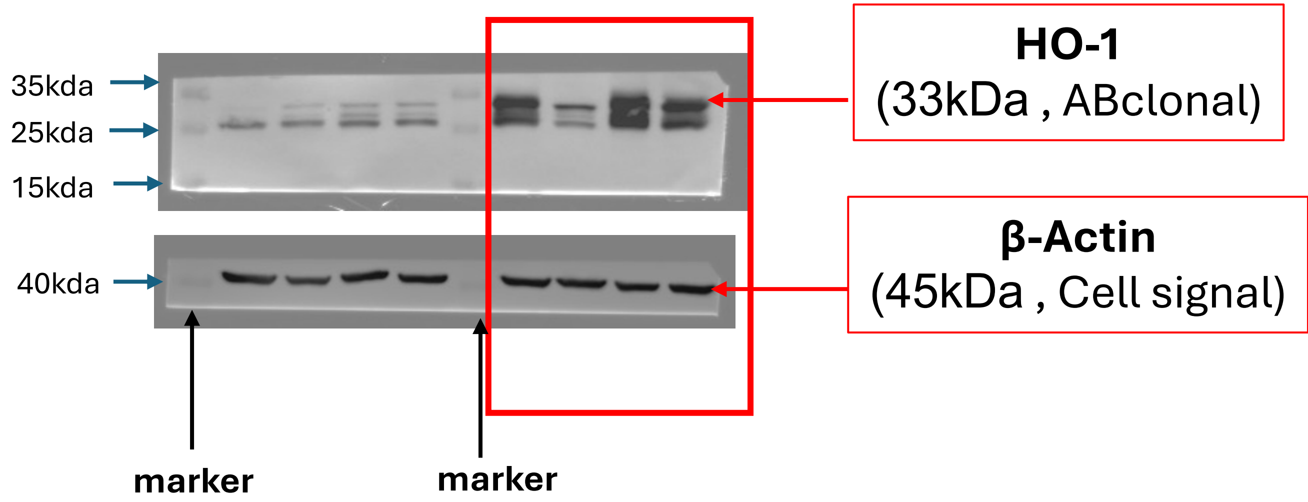

35kda
25kda
15kda
40kda
marker
marker
HO-1
(33kDa , ABclonal)
β-Actin
(45kDa , Cell signal)

raw data for Figure 4C

HO-1
(33kDa , ABclonal)

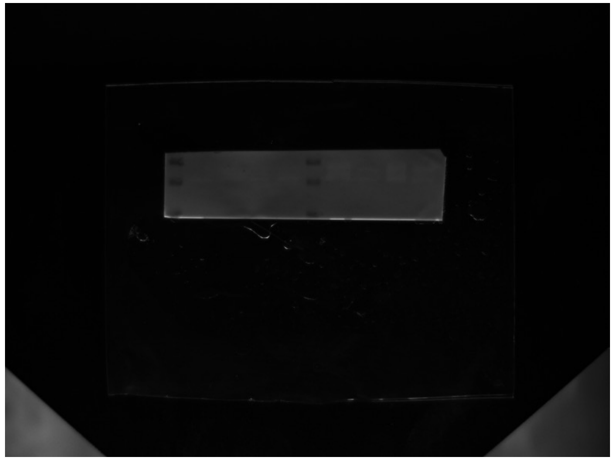

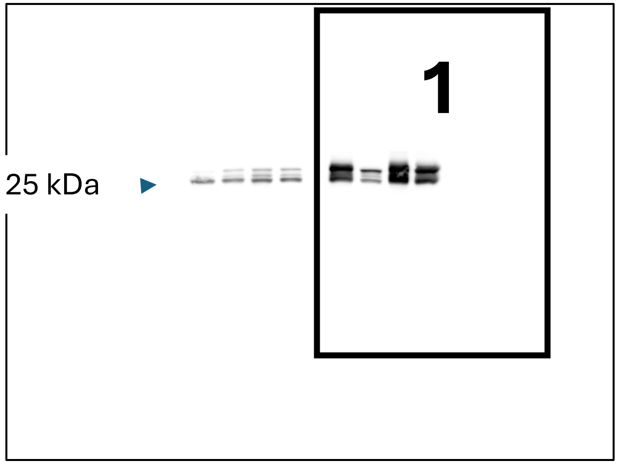

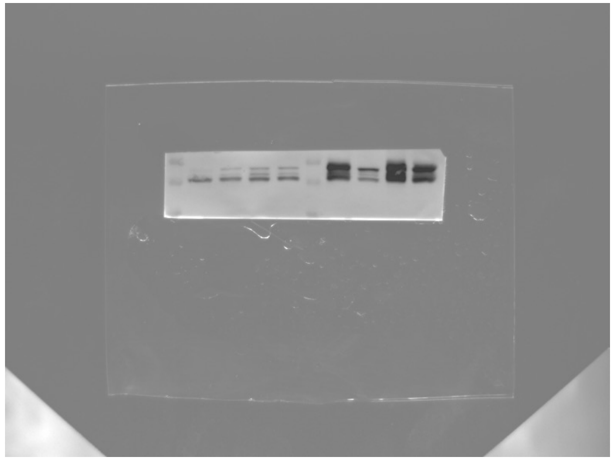

β-Actin
(45kDa , Cell signal)

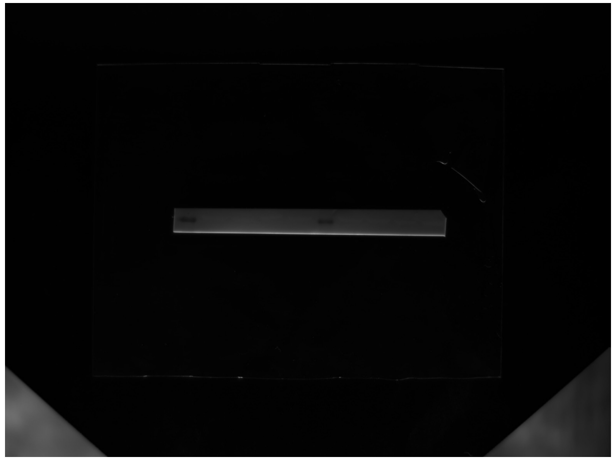

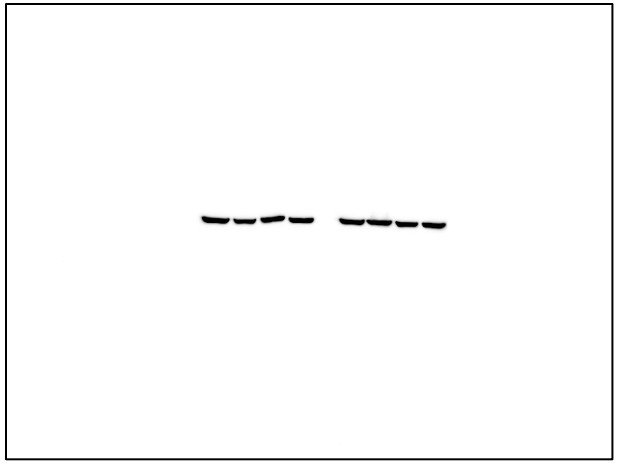

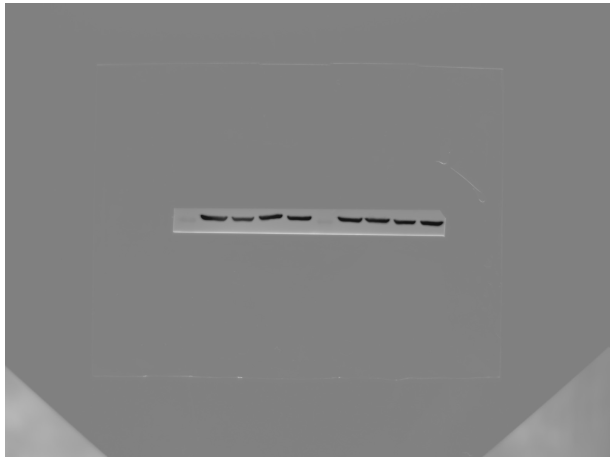

raw data for Figure 4C

HO-1
(33kDa , ABclonal)

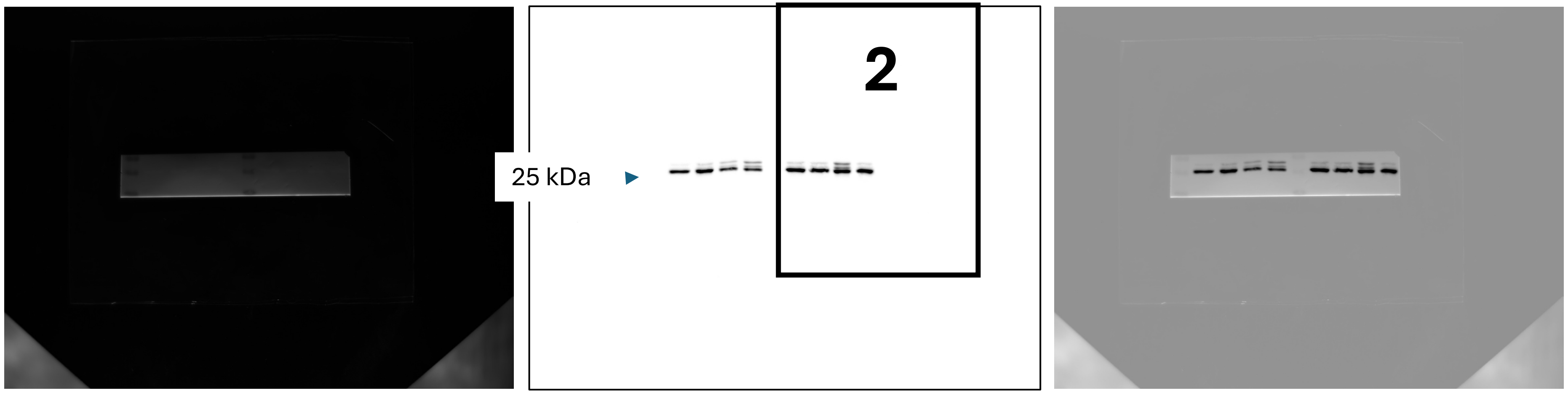

β-Actin
(45kDa , Cell signal)

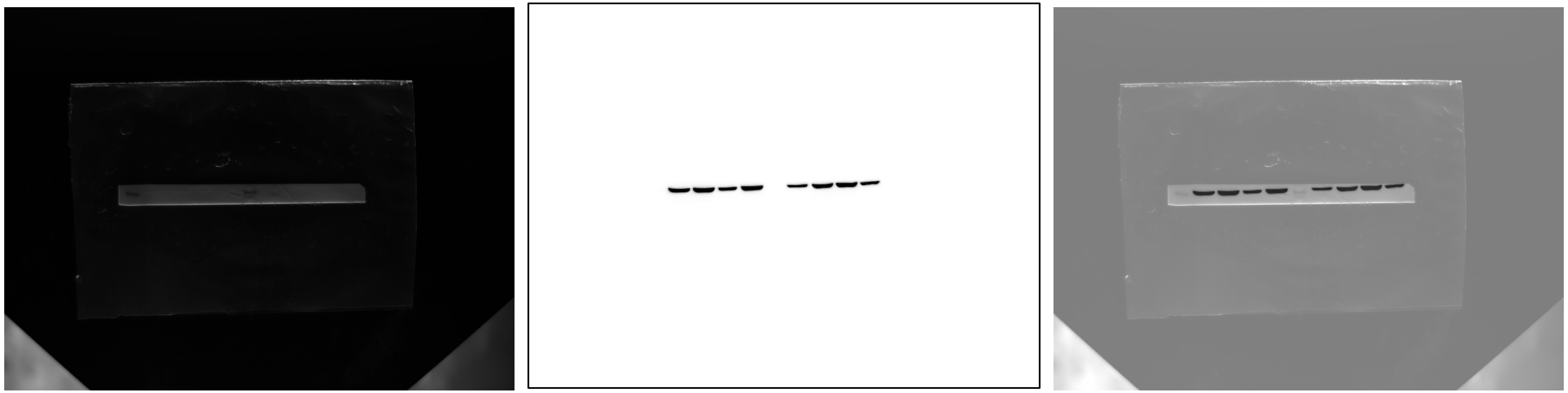

raw data for Figure 4C

HO-1
(33kDa , ABclonal)

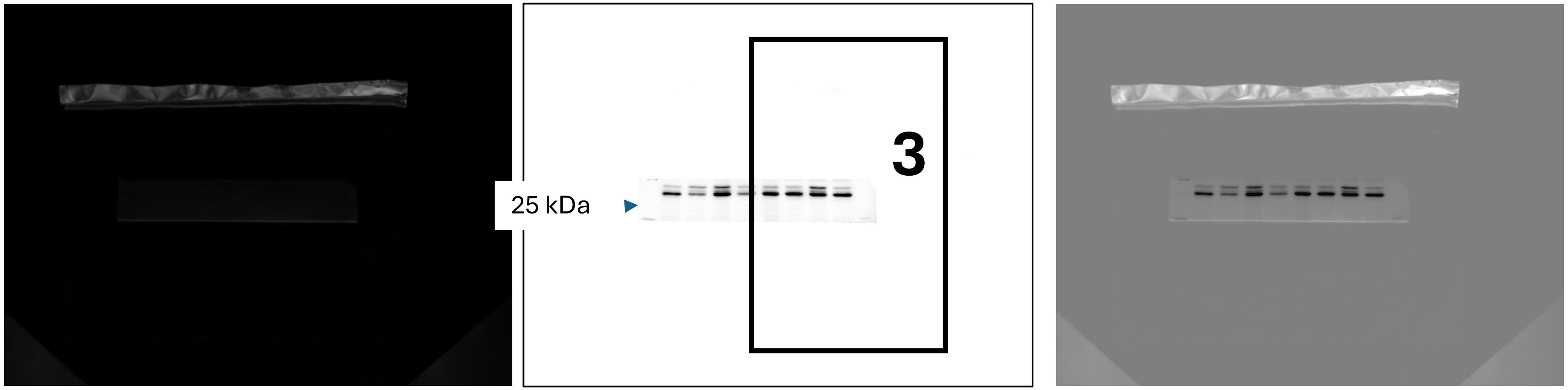

β-Actin
(45kDa , Cell signal)

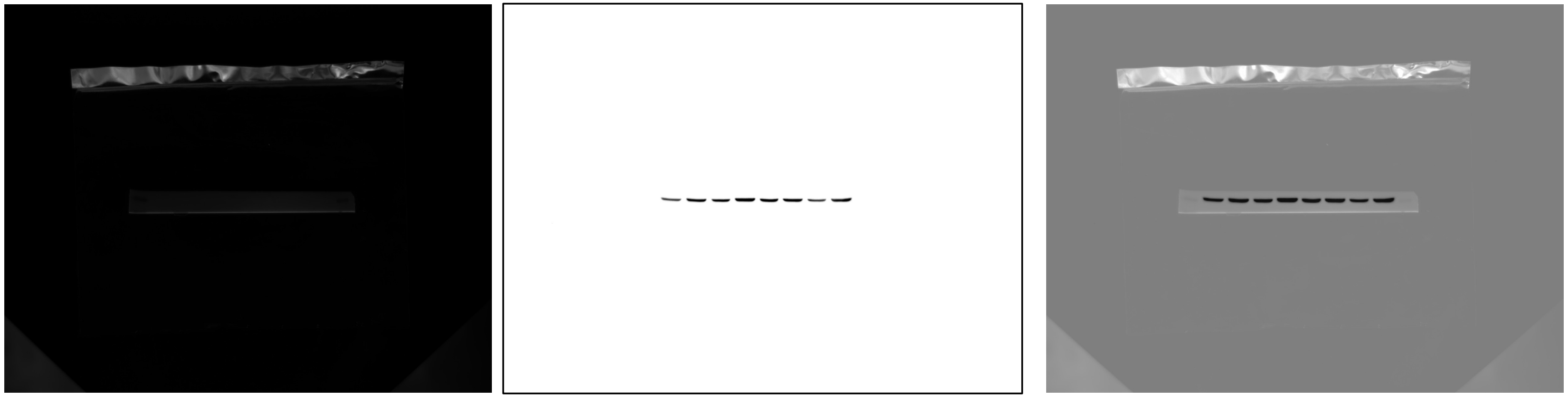

Figure 4D

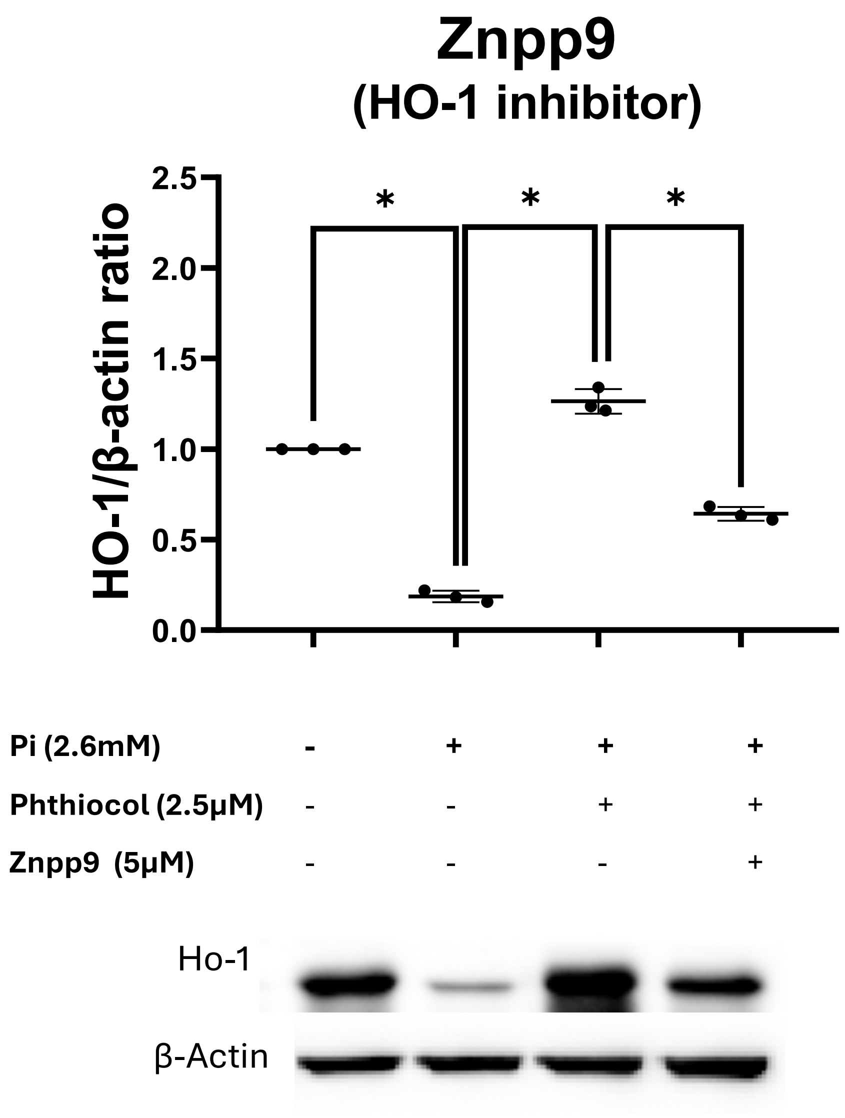
Znpp9
(HO-1 inhibitor)
HO-1/β-actin ratio
2.5
2.0
1.5
1.0
0.5
0.0
*
*
*
Pi (2.6mM) - + + +
Phthiocol (2.5μM) - - + +
Znpp9 (5μM) - - - +
Ho-1
β-Actin

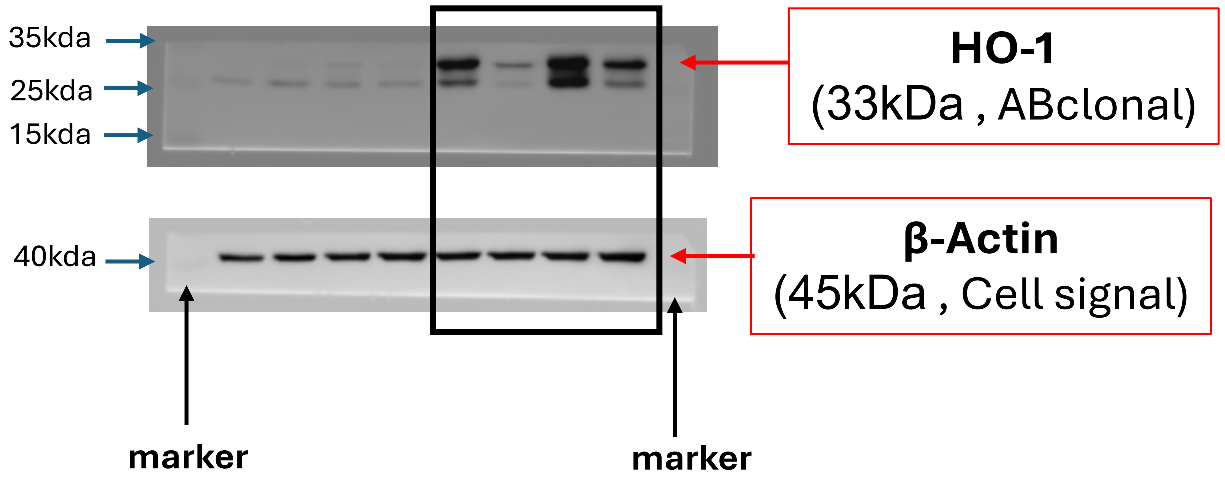
35kda
25kda
15kda
40kda
HO-1
(33kDa , ABclonal)
β-Actin
(45kDa , Cell signal)
marker
marker

raw data for Figure 4D

HO-1
(33kDa , ABclonal)

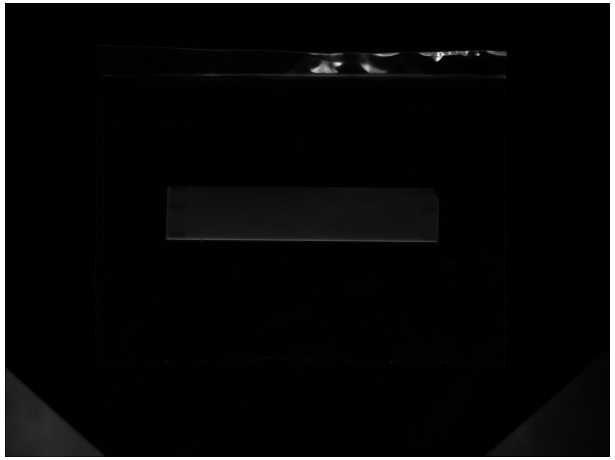

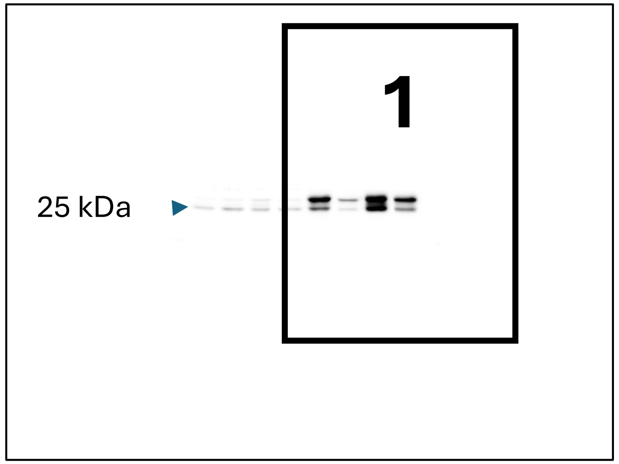

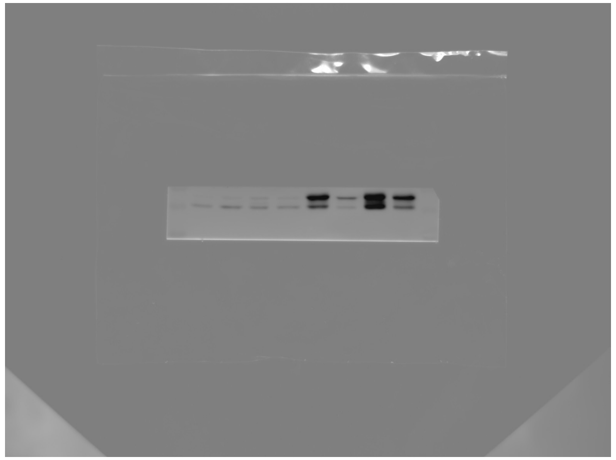

β-Actin
(45kDa , Cell signal)

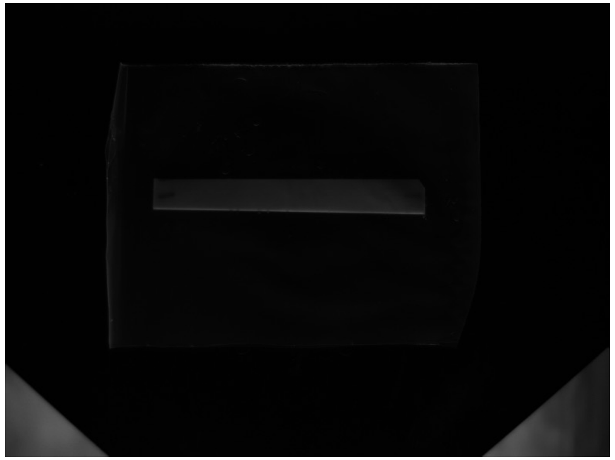

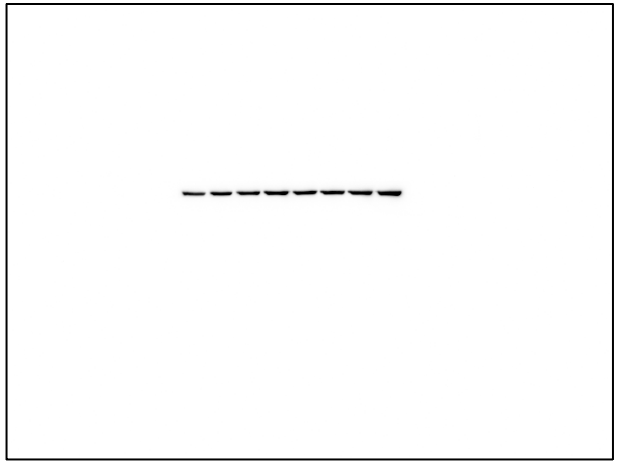

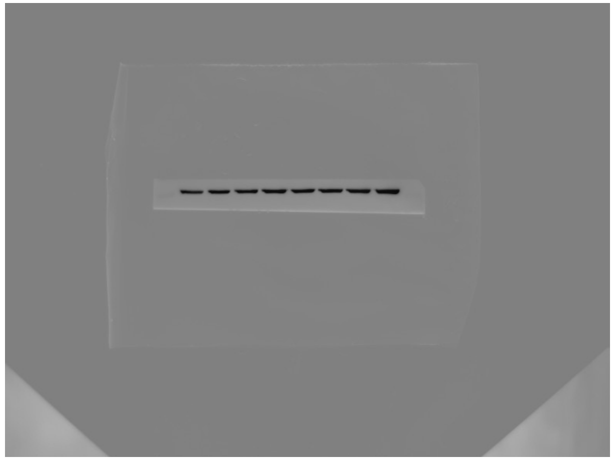

raw data for Figure 4D

HO-1
(33kDa , ABclonal)

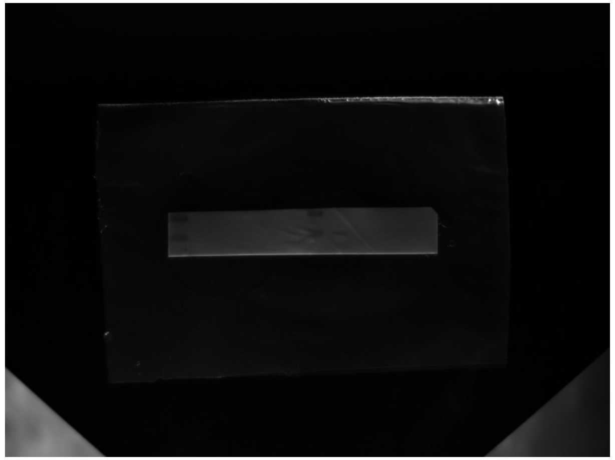

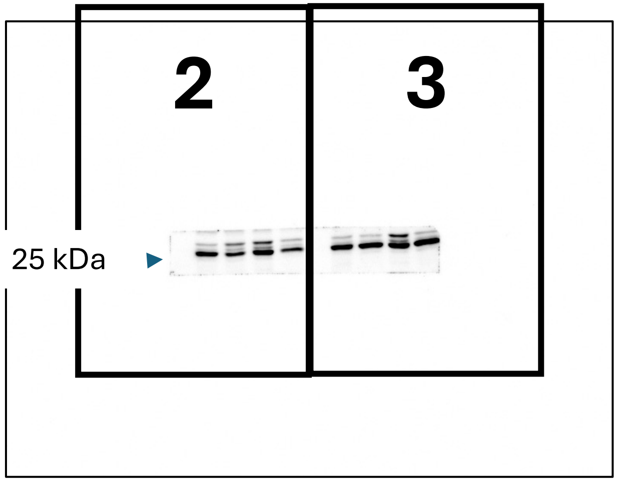

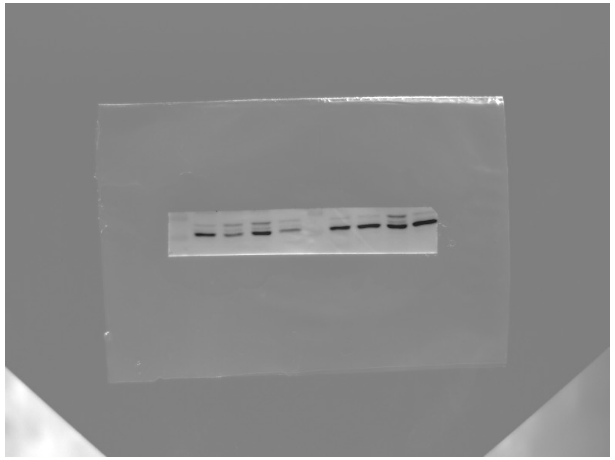

β-Actin
(45kDa , Cell signal)

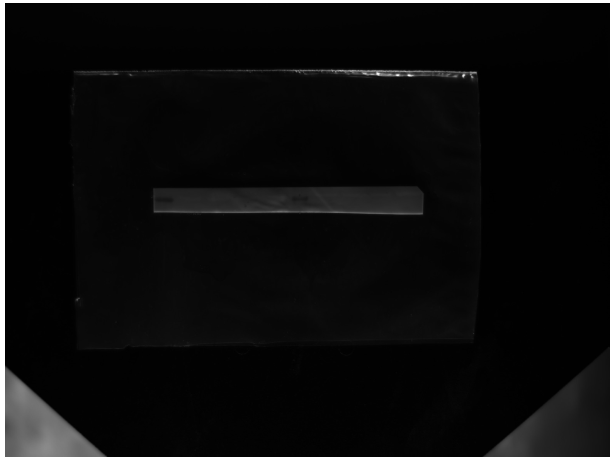

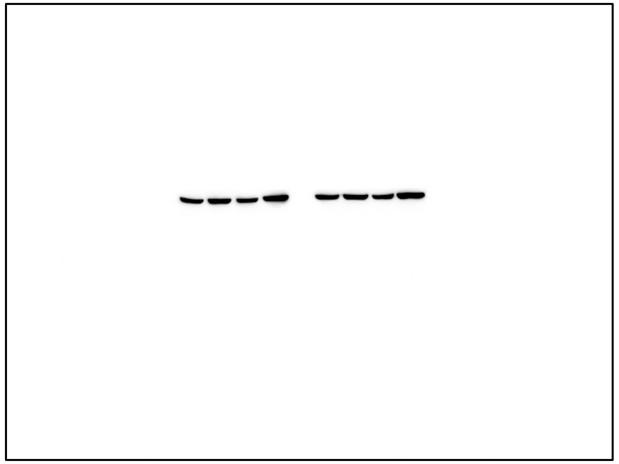

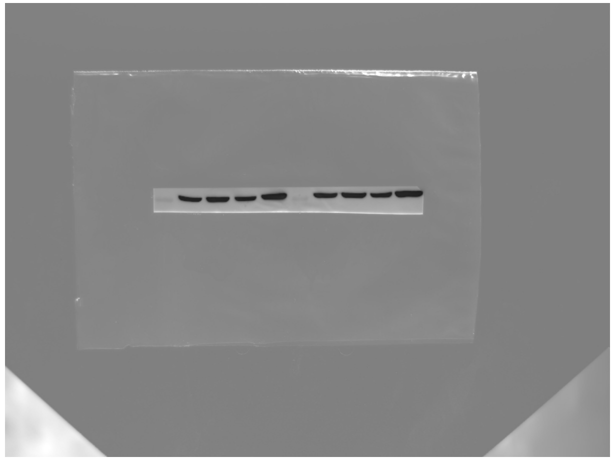

Figure 4E

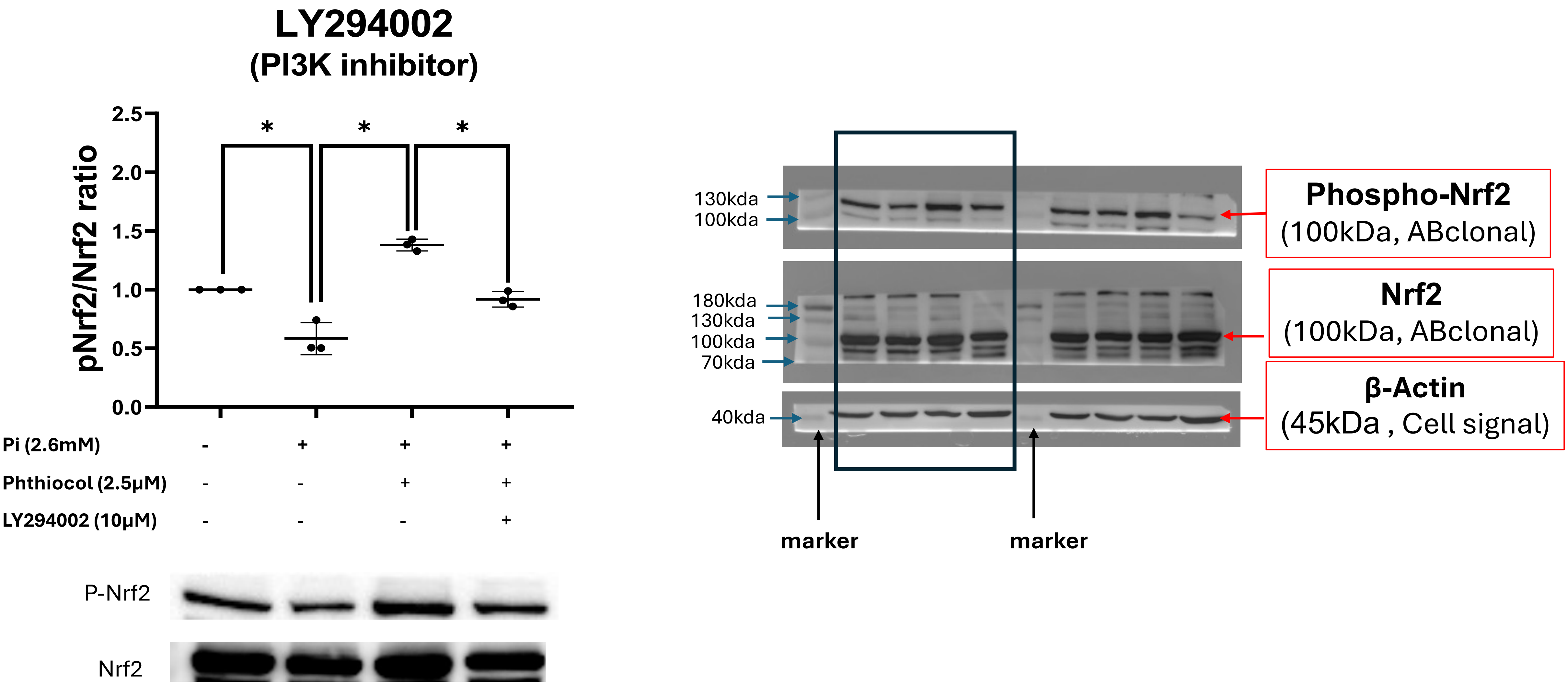

LY294002
(PI3K inhibitor)
pNrf2/Nrf2 ratio
2.5
2.0
1.5
1.0
0.5
0.0
*
*
*
Pi (2.6mM) - + + +
Phthiocol (2.5μM) - - + +
LY294002 (10μM) - - - +
P-Nrf2
Nrf2
130kda
100kda
180kda
130kda
100kda
70kda
40kda
marker
marker
Phospho-Nrf2
(100kDa, ABclonal)
Nrf2
(100kDa, ABclonal)
β-Actin
(45kDa , Cell signal)

raw data for Figure 4E

Phospho-Nrf2
(100kDa, ABclonal)

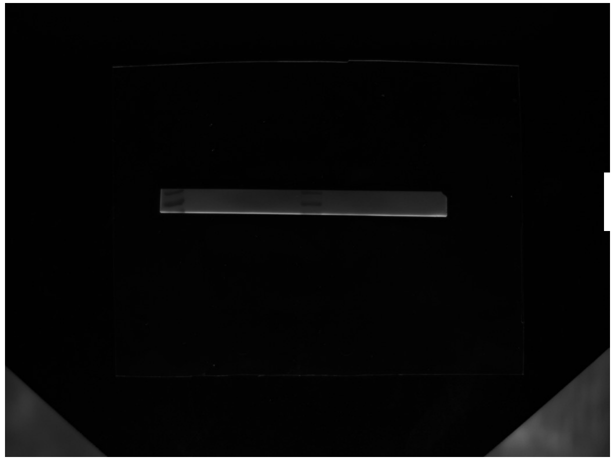

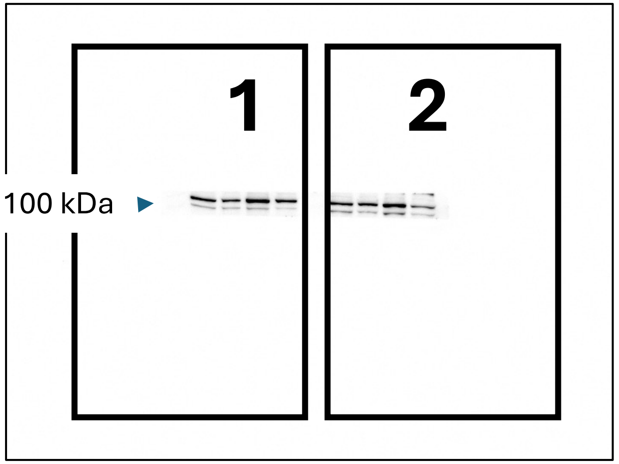

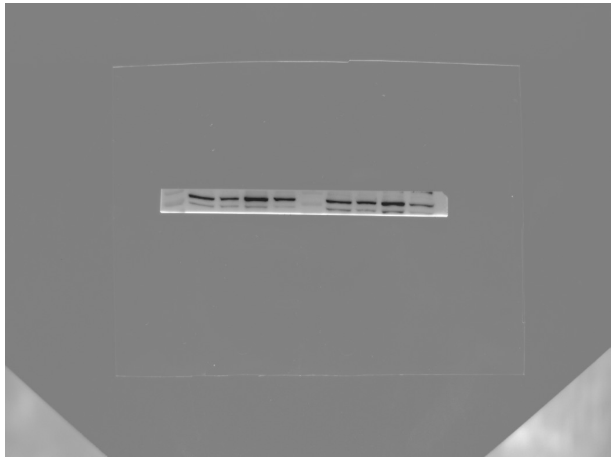

Nrf2
(100kDa, ABclonal)

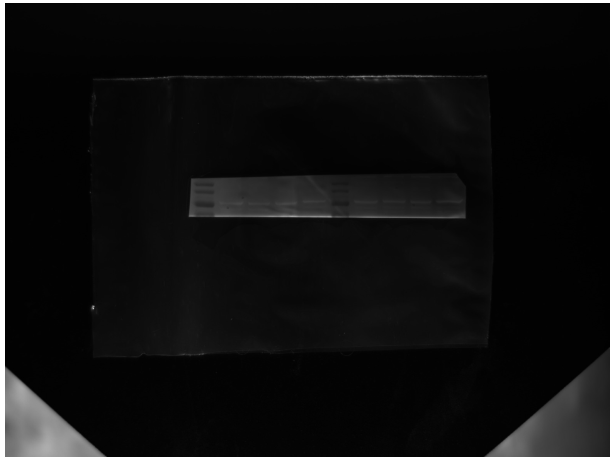

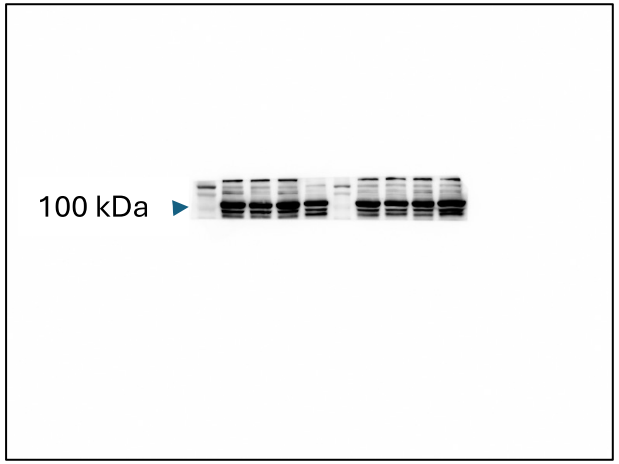

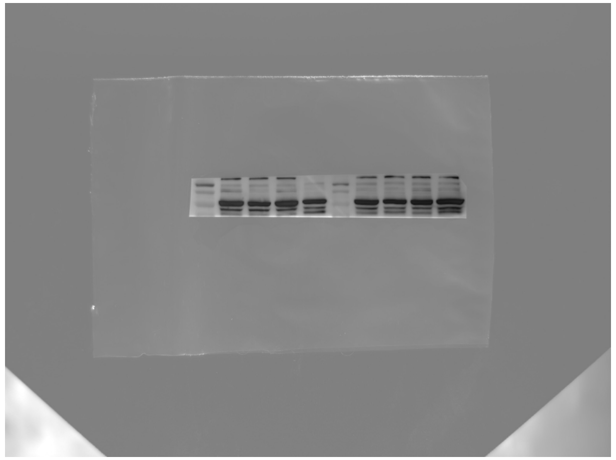

β-Actin
(45kDa , Cell signal)

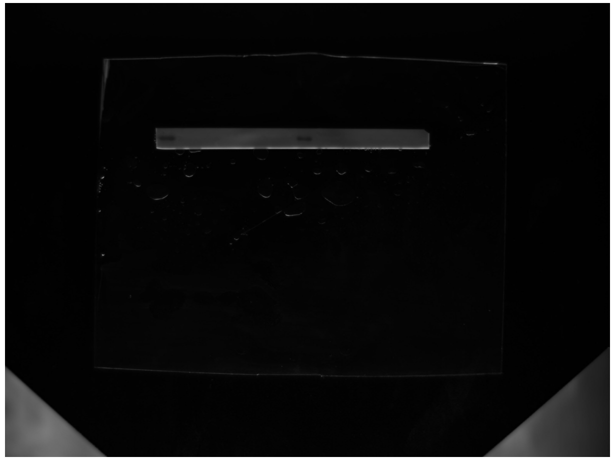

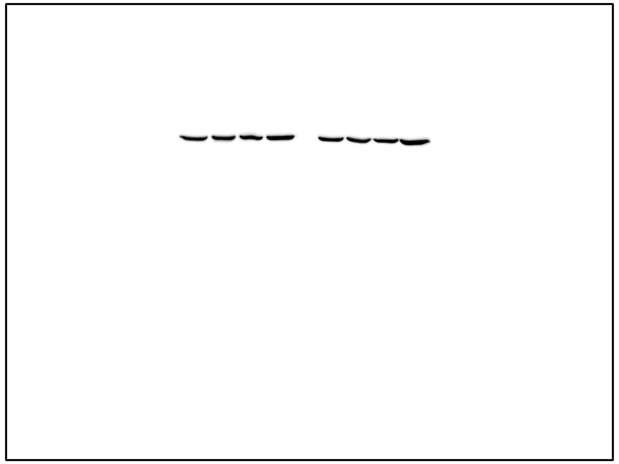

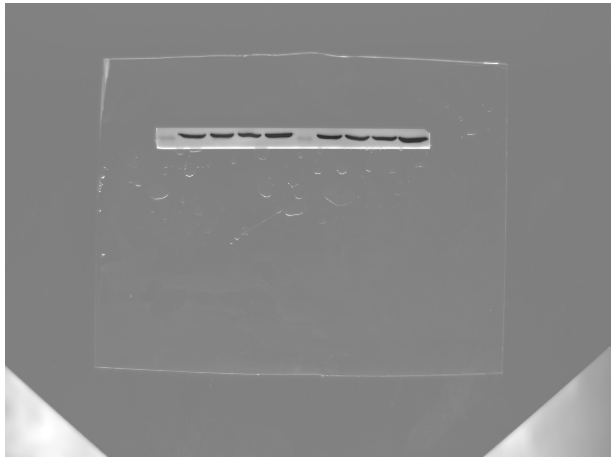

raw data for Figure 4E

Phospho-Nrf2
(100kDa, ABclonal)

Nrf2
(100kDa, ABclonal)

β-Actin
(45kDa , Cell signal)

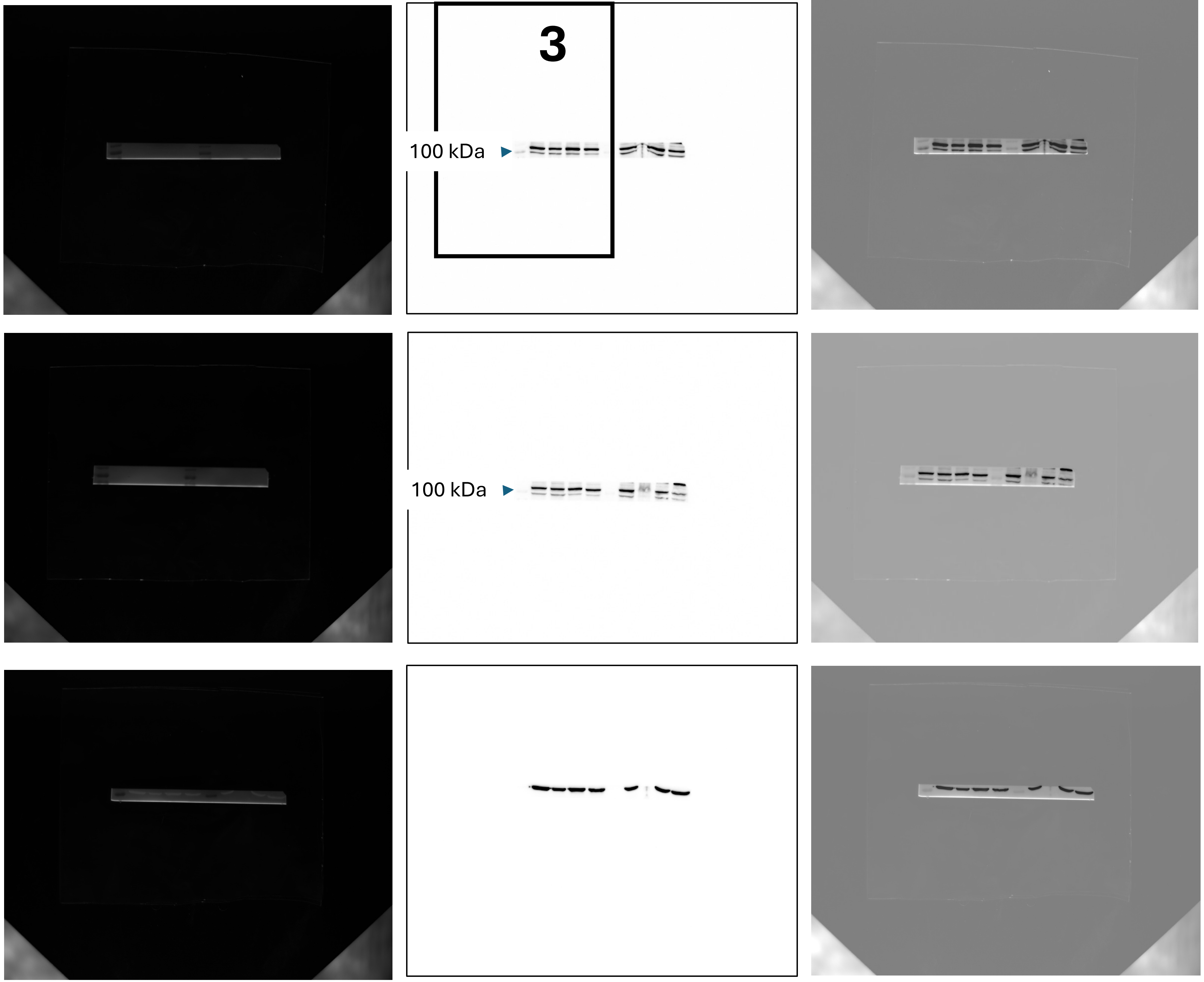

Figure 4F

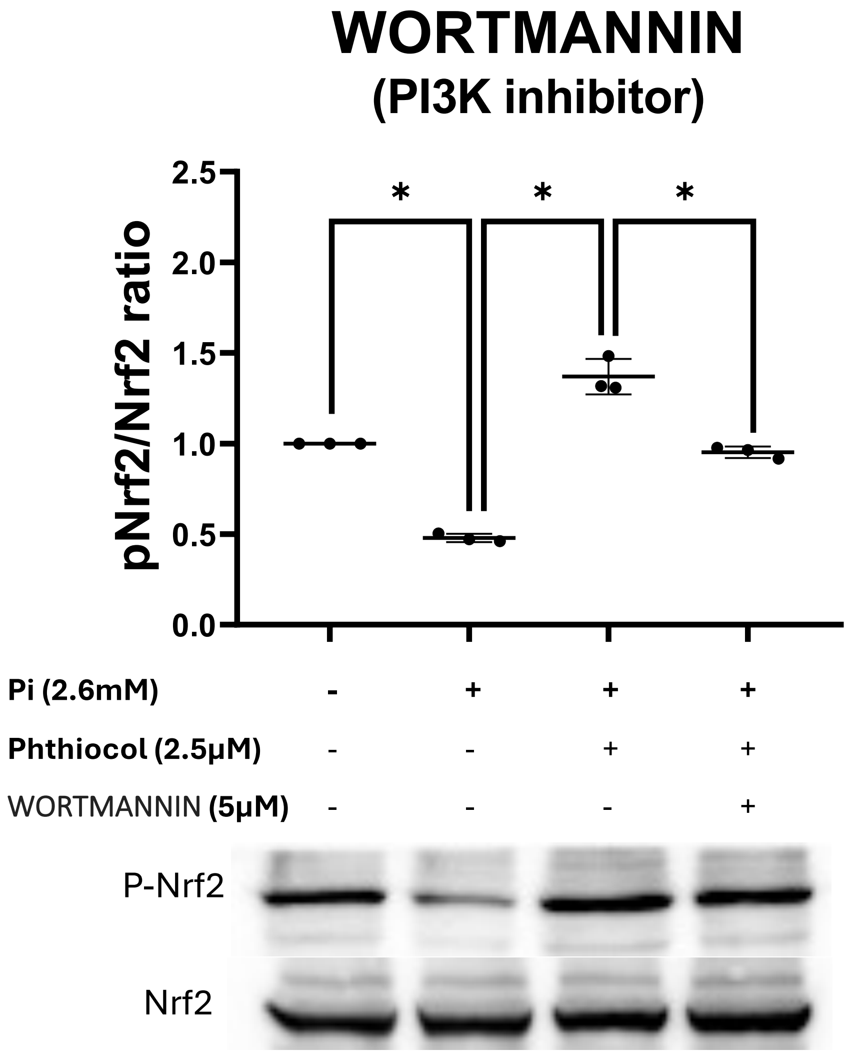
WORTMANNIN
(PI3K inhibitor)
pNrf2/Nrf2 ratio
2.5
2.0
1.5
1.0
0.5
0.0
*
*
*
Pi (2.6mM) - + + +
Phthiocol (2.5μM) - - + +
WORTMANNIN (5μM) - - - +
P-Nrf2
Nrf2

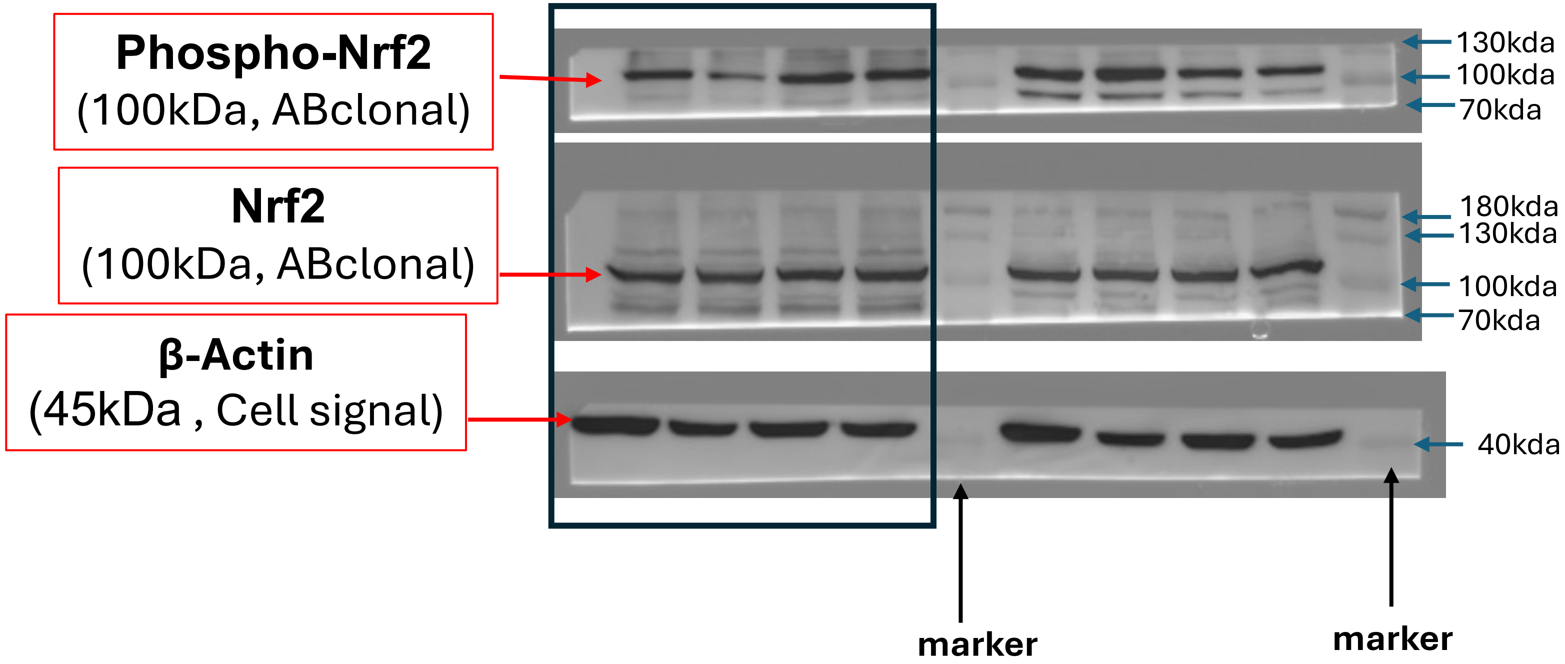
Phospho-Nrf2
(100kDa, ABclonal)
Nrf2
(100kDa, ABclonal)
β-Actin
(45kDa , Cell signal)
130kda
100kda
70kda
180kda
130kda
100kda
70kda
40kda
marker
marker

raw data for Figure 4F

Phospho-Nrf2
(100kDa, ABclonal)

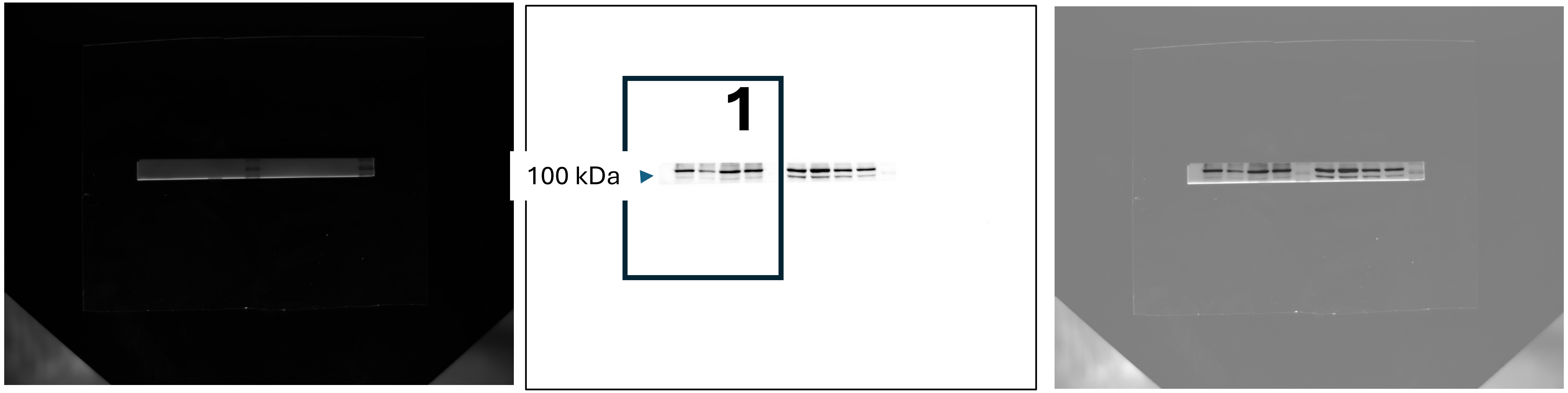

Nrf2
(100kDa, ABclonal)

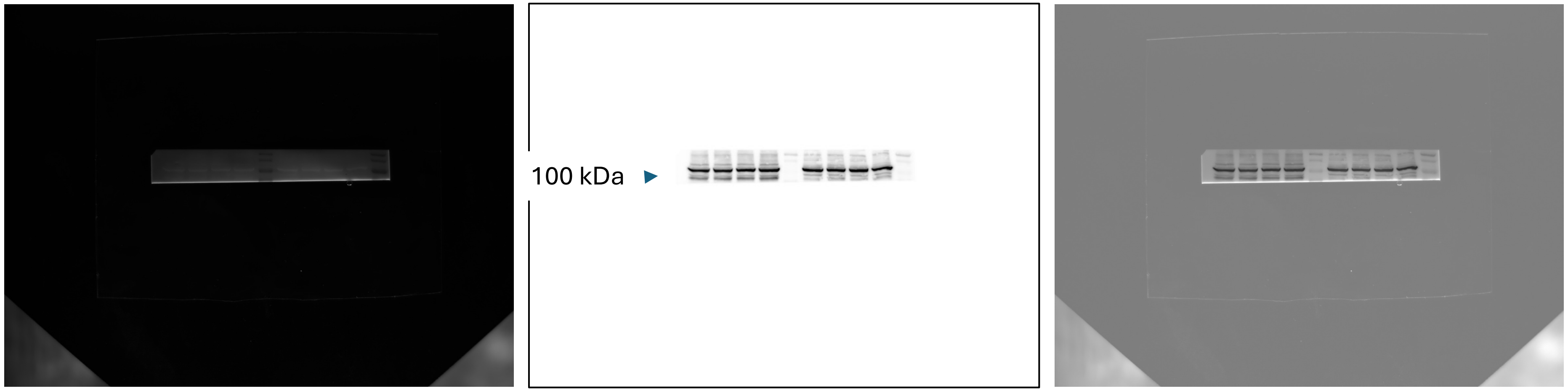

β-Actin
(45kDa , Cell signal)

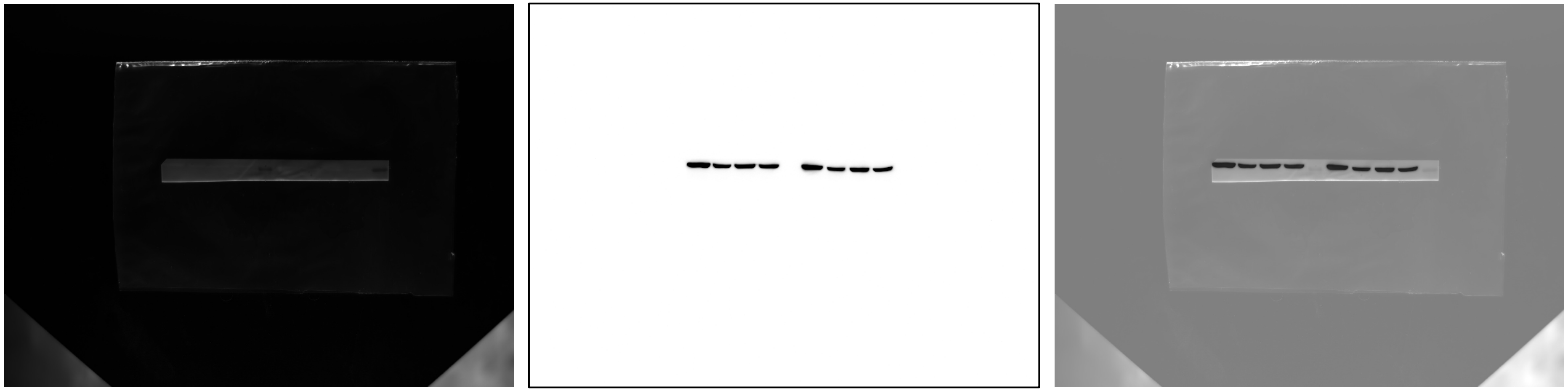

raw data for Figure 4F

Phospho-Nrf2
(100kDa, ABclonal)

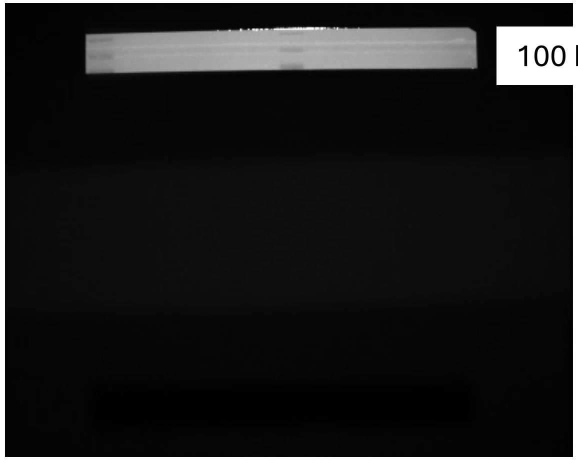

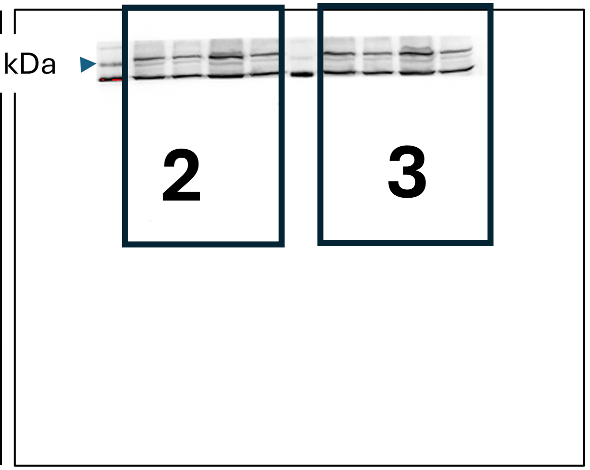

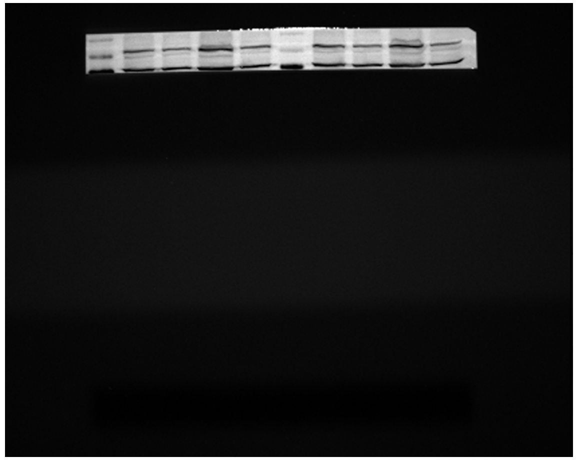

Nrf2
(100kDa, ABclonal)

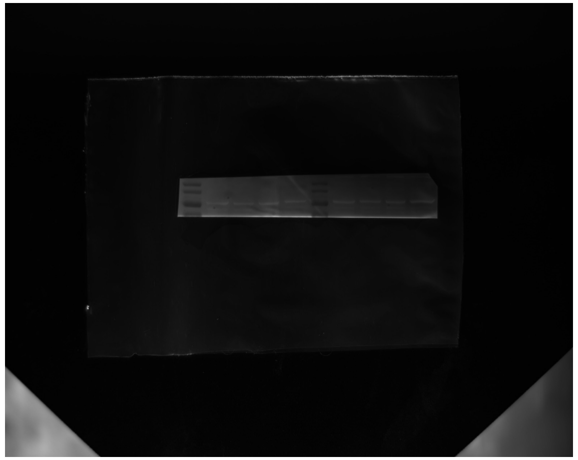

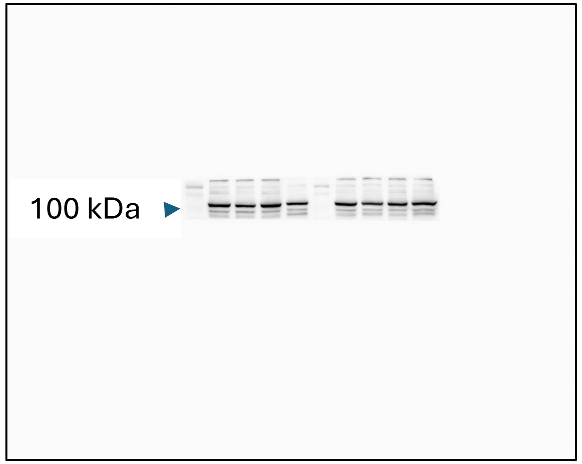

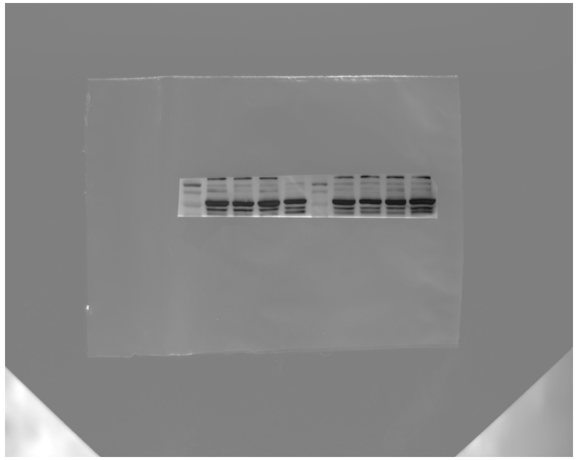

β-Actin
(45kDa , Cell signal)

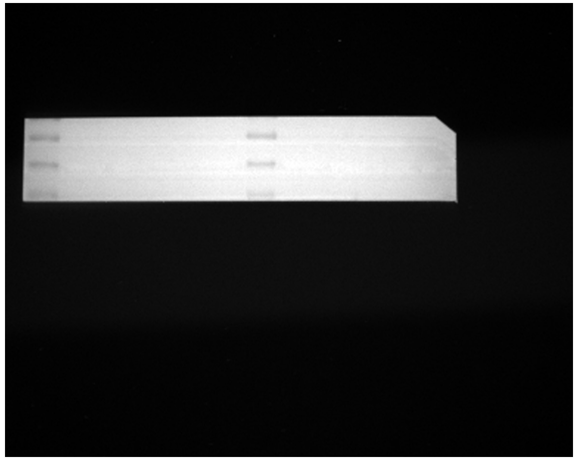

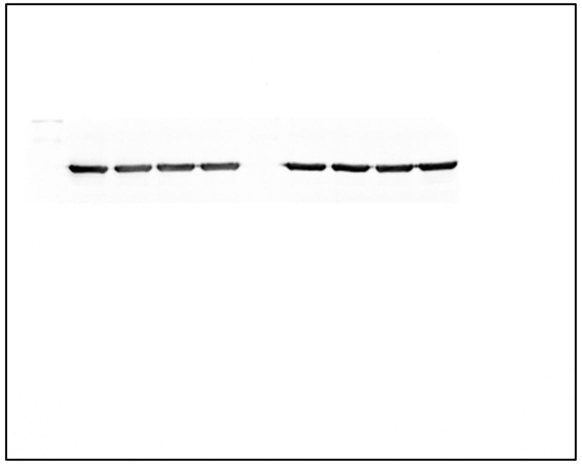

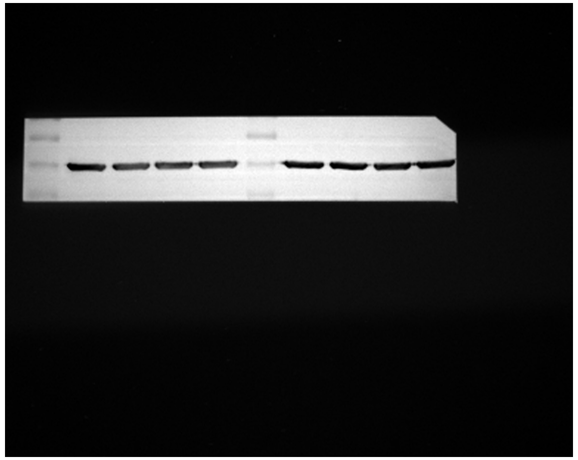

Figure 4G

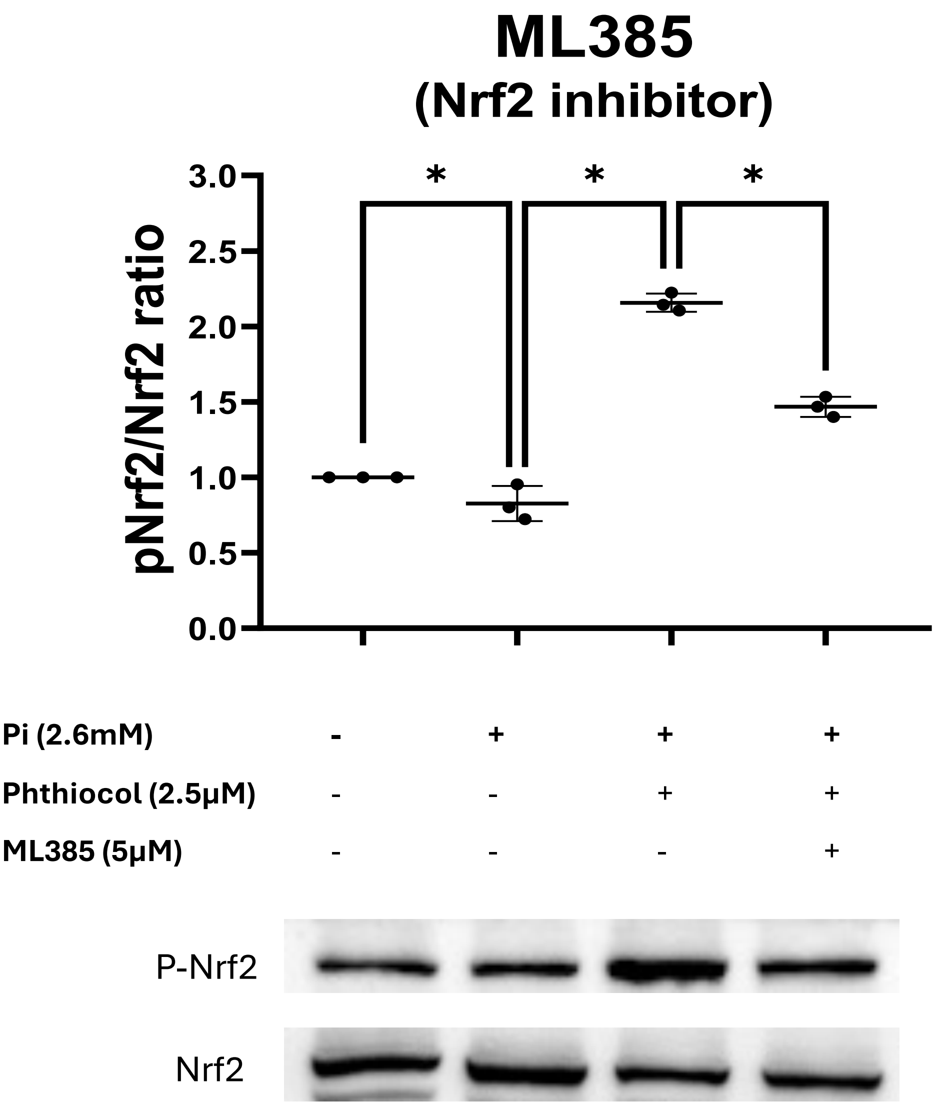

ML385
(Nrf2 inhibitor)
pNrf2/Nrf2 ratio
3.0
2.5
2.0
1.5
1.0
0.5
0.0
*
*
*
Pi (2.6mM) - + + +
Phthiocol (2.5μM) - - + +
ML385 (5μM) - - - +
P-Nrf2
Nrf2

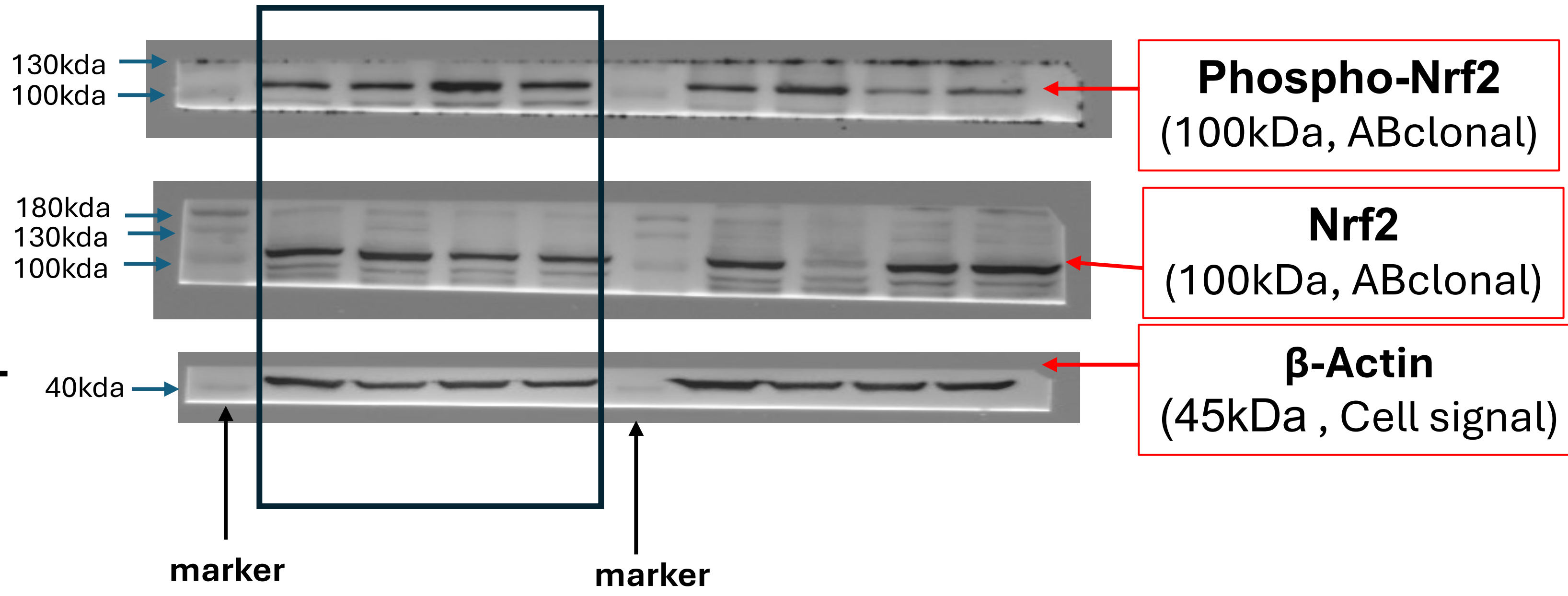

130kda
100kda
180kda
130kda
100kda
40kda
Phospho-Nrf2
(100kDa, ABclonal)
Nrf2
(100kDa, ABclonal)
β-Actin
(45kDa , Cell signal)
marker
marker

raw data for Figure 4G

Phospho-Nrf2
(100kDa, ABclonal)

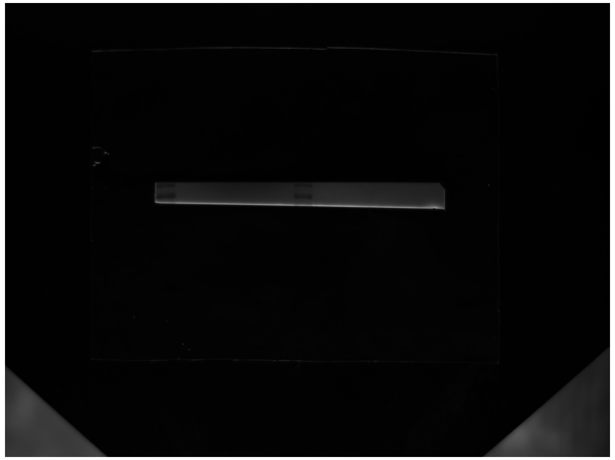
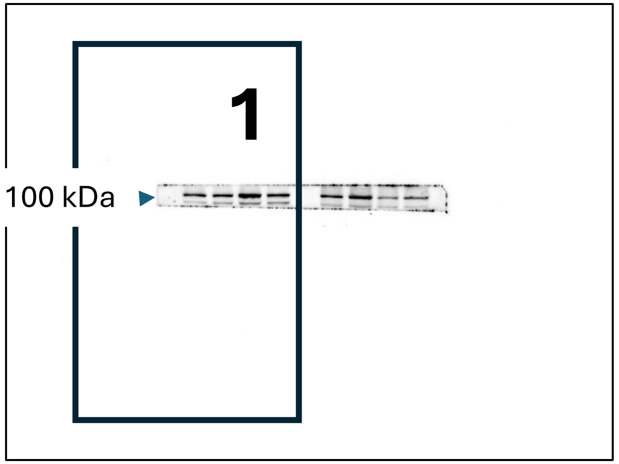

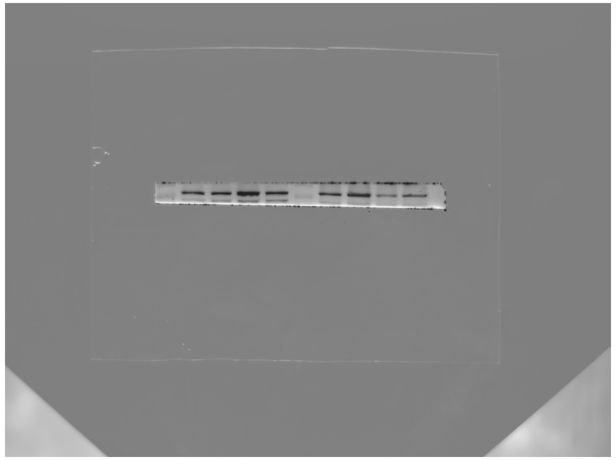

Nrf2
(100kDa, ABclonal)

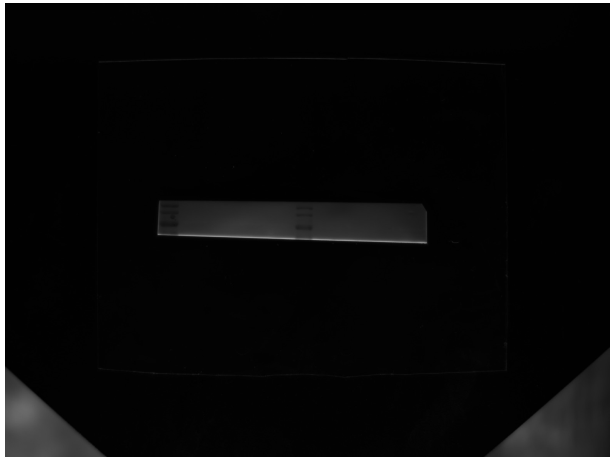
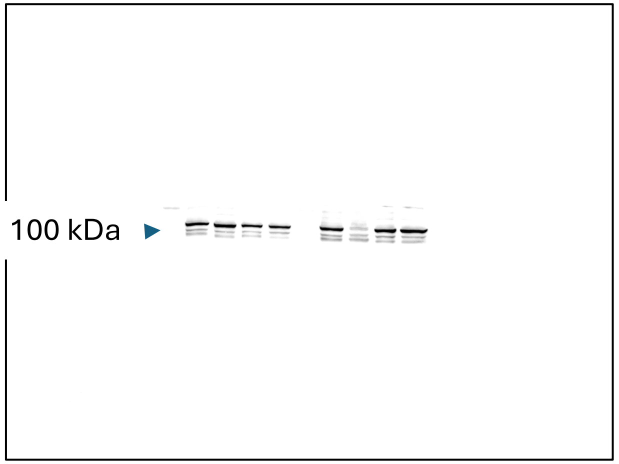

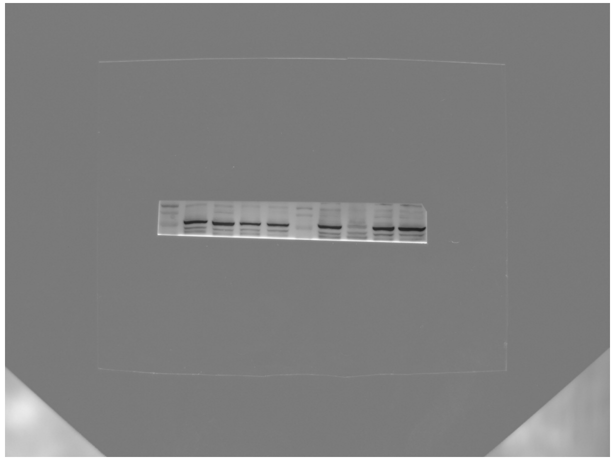

β-Actin
(45kDa , Cell signal)

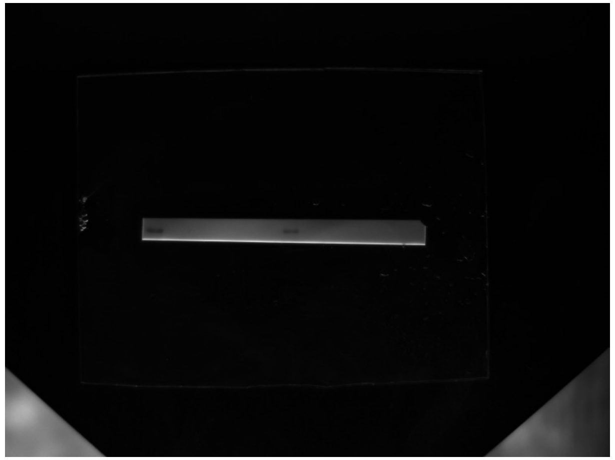
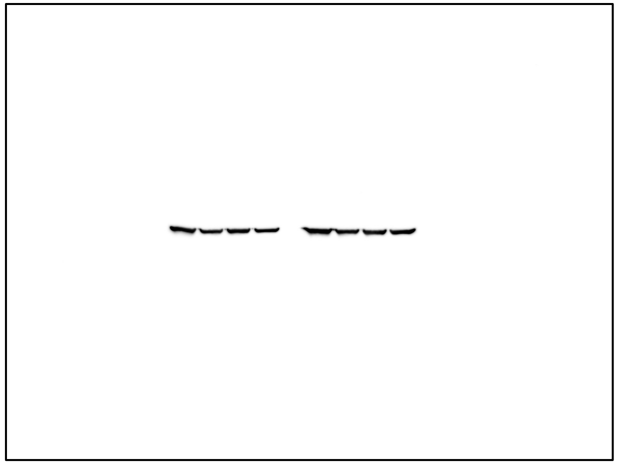
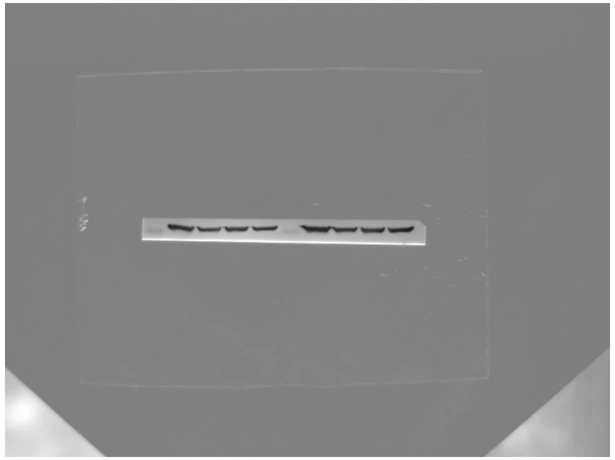

raw data for Figure 4G

Phospho-Nrf2
(100kDa, ABclonal)

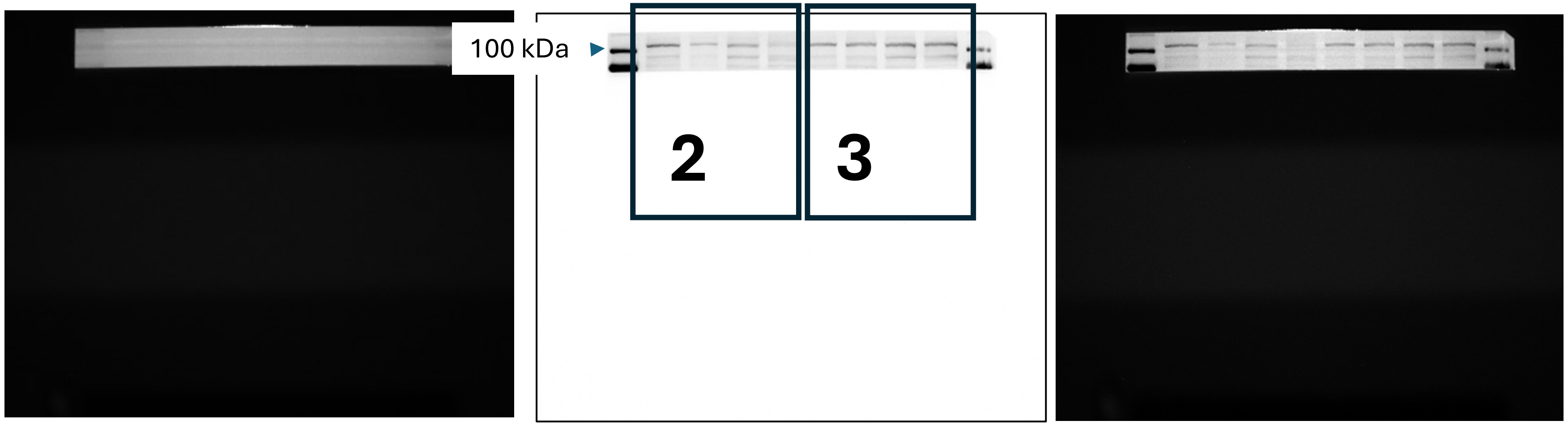

Nrf2
(100kDa, ABclonal)

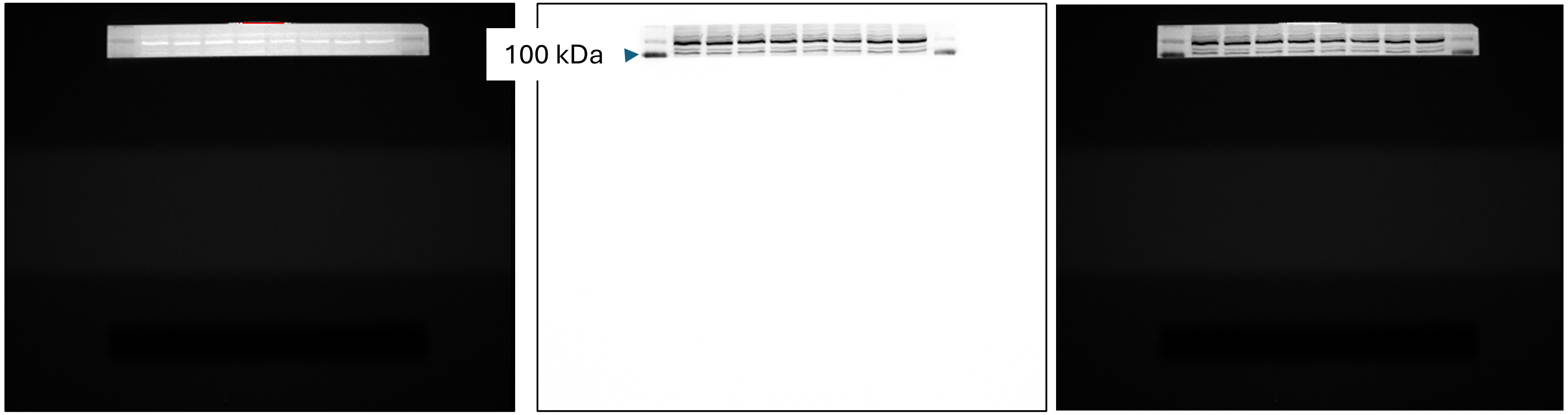

β-Actin
(45kDa , Cell signal)

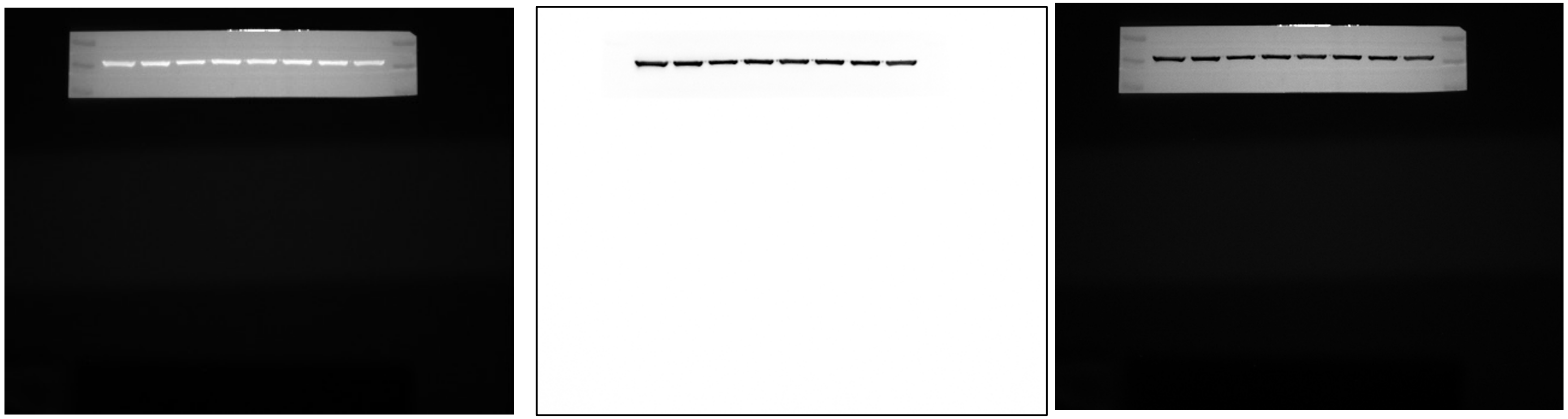

Figure 4H

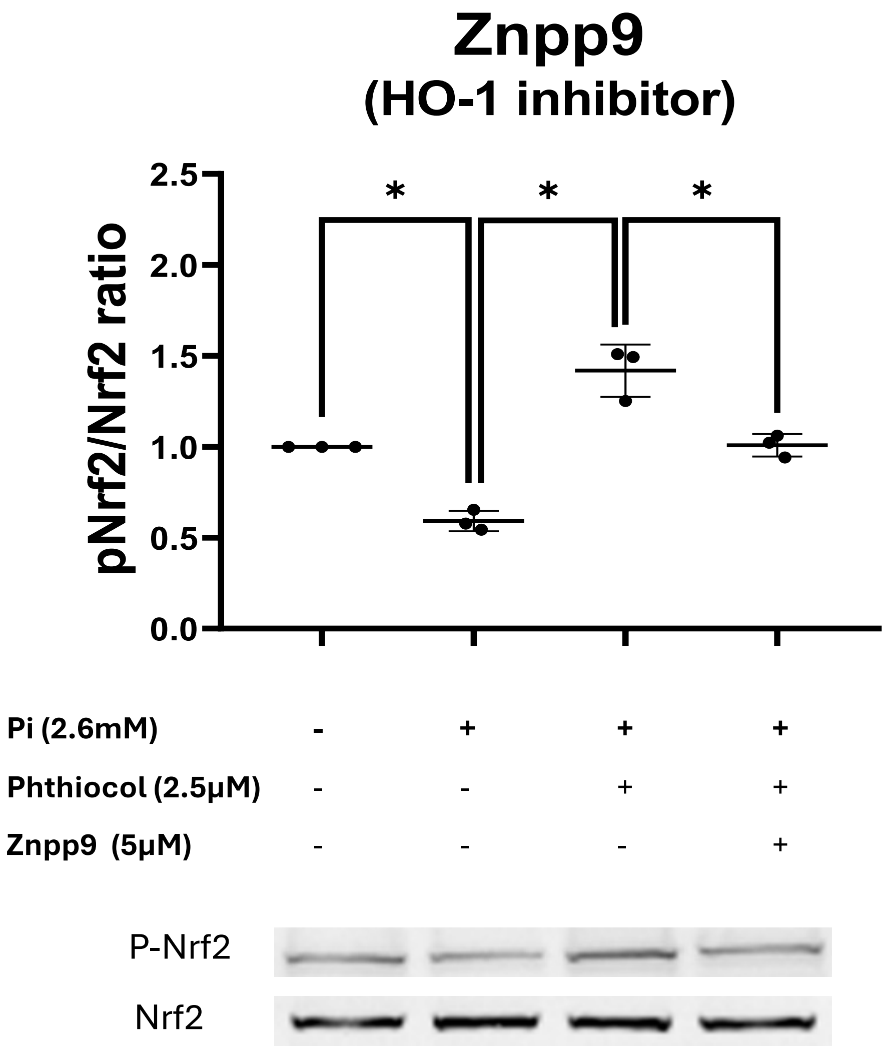
Znpp9
(HO-1 inhibitor)
pNrf2/Nrf2 ratio
2.5
2.0
1.5
1.0
0.5
0.0
*
*
*
Pi (2.6mM) - + + +
Phthiocol (2.5μM) - - + +
Znpp9 (5μM) - - - +
P-Nrf2
Nrf2

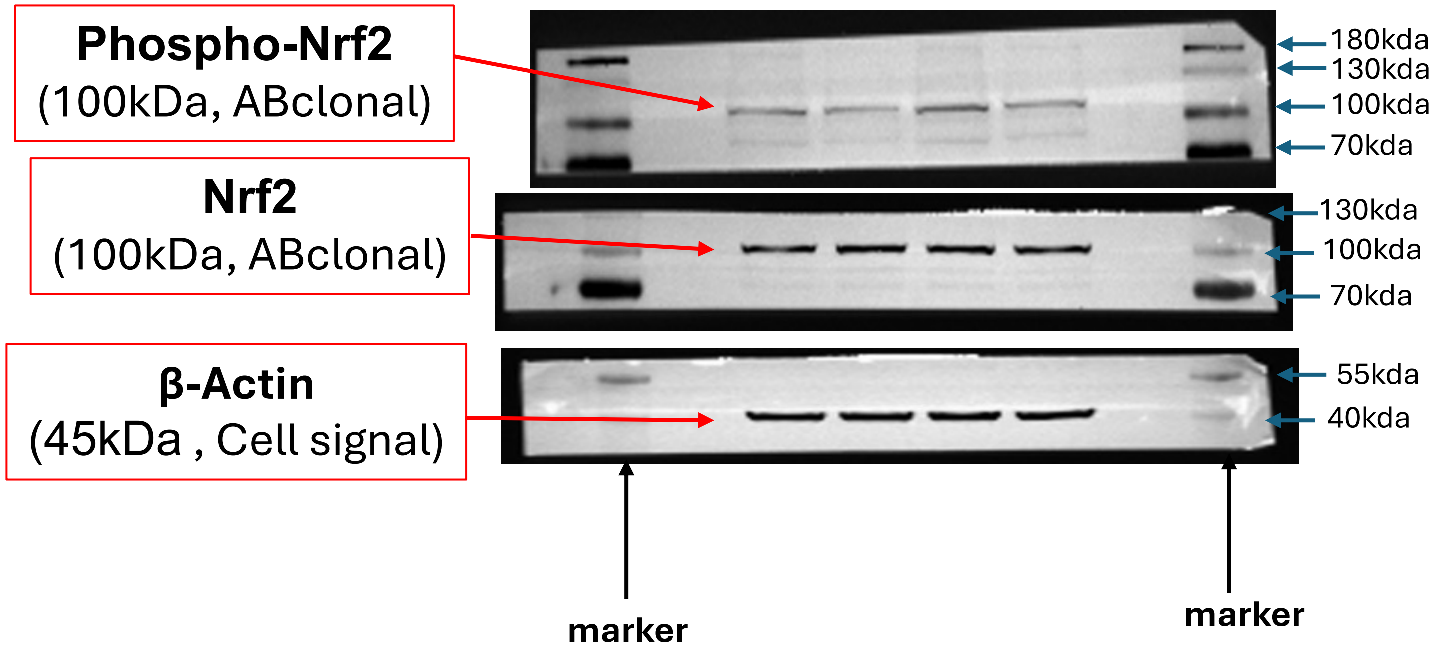
Phospho-Nrf2
(100kDa, ABclonal)
Nrf2
(100kDa, ABclonal)
β-Actin
(45kDa , Cell signal)
180kda
130kda
100kda
70kda
130kda
100kda
70kda
55kda
40kda
marker
marker

raw data for Figure 4H

Phospho-Nrf2
(100kDa, ABclonal)

Nrf2
(100kDa, ABclonal)

β-Actin
(45kDa , Cell signal)

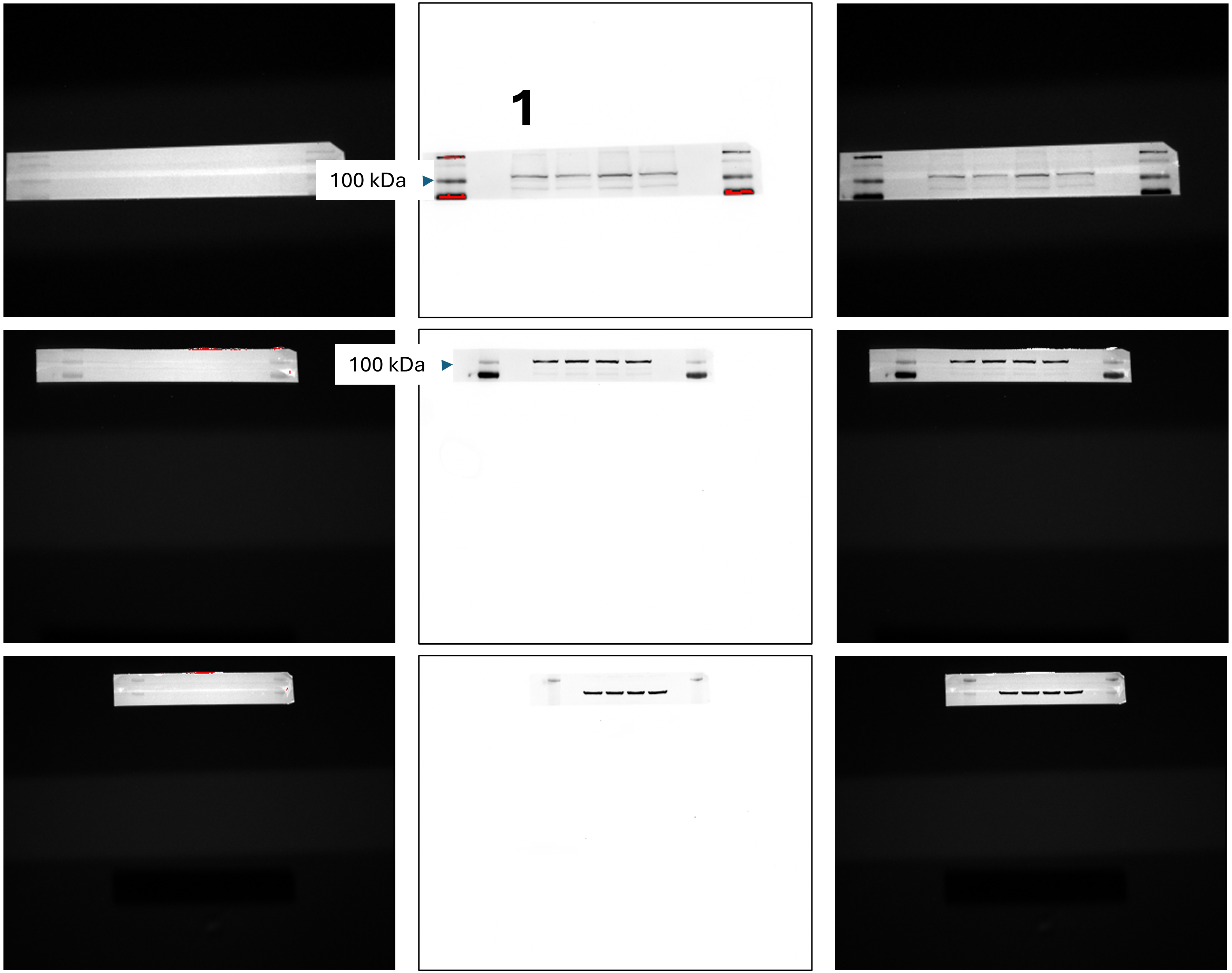

raw data for Figure 4H

Phospho-Nrf2
(100kDa, ABclonal)

Nrf2
(100kDa, ABclonal)

β-Actin
(45kDa , Cell signal)

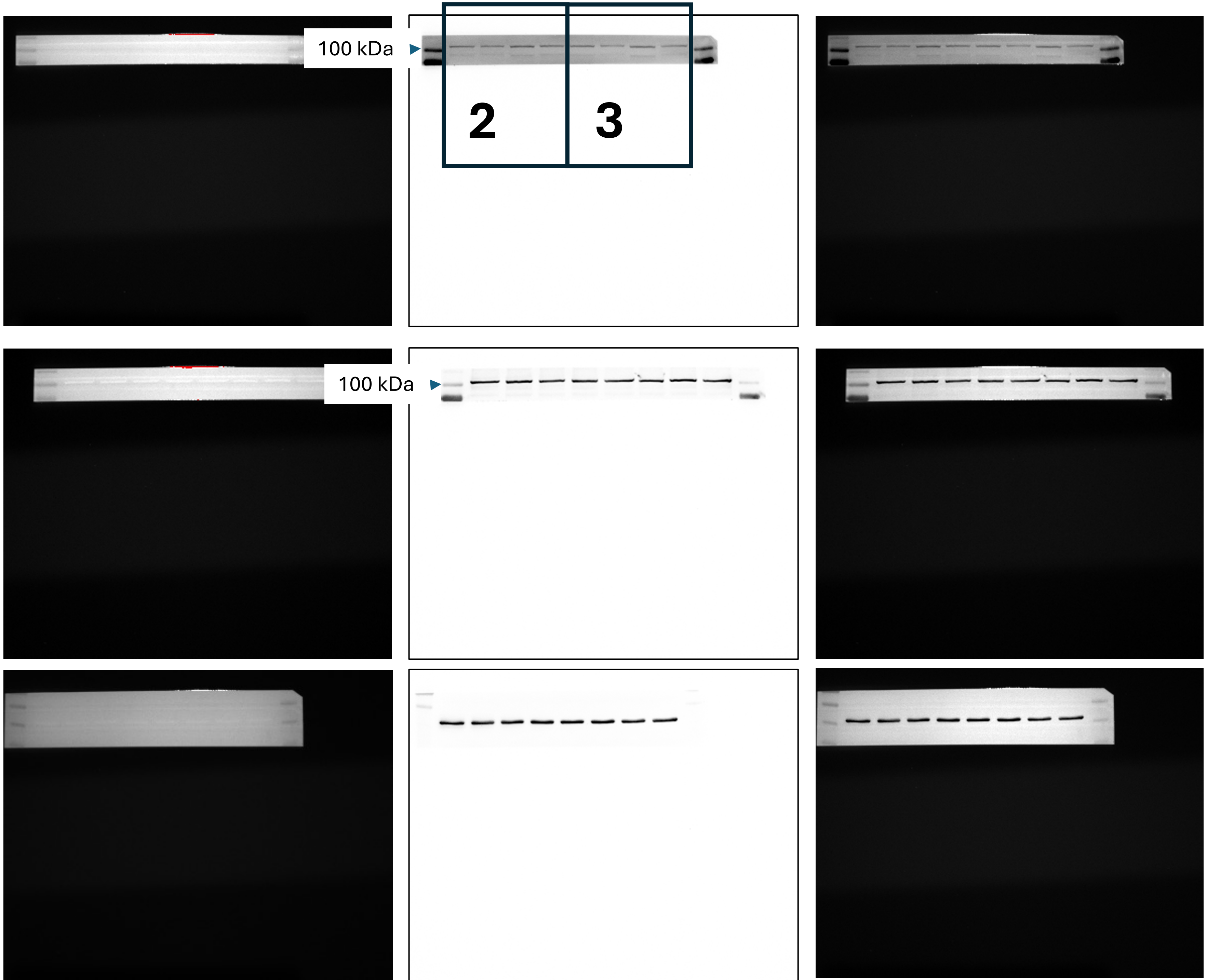

Supplement: Supplementary file 1 [file antioxidants-14-01328-s001.zip › antioxidants-3901514-supplementary.pdf]
